# Highly functionalized β-lactams and 2-ketopiperazines as TRPM8 antagonists with antiallodynic activity

**DOI:** 10.1038/s41598-020-70691-x

**Published:** 2020-08-25

**Authors:** M.Ángeles Bonache, Cristina Martín-Escura, Roberto de la Torre Martínez, Alicia Medina, Sara González-Rodríguez, Andrés Francesch, Carmen Cuevas, Ana María Roa, Gregorio Fernández-Ballester, Antonio Ferrer-Montiel, Asia Fernández-Carvajal, Rosario González-Muñiz

**Affiliations:** 1 Instituto de Química Médica (IQM-CSIC), Juan de la Cierva 3, 28006 Madrid, Spain; 2 IDiBE, Universidad Miguel Hernández, Avda. de la Universidad s/n, 03202 Elche, Spain; 3 PharmaMar S.A, Avda. de los Reyes 1, 28770 Colmenar Viejo, Spain; 4 Alodia Farmacéutica SL, Santiago Grisolia 2, Tres Cantos, 28760 Madrid, Spain

**Keywords:** Chemical biology, Ion channels, Transient receptor potential channels, Chemistry, Medicinal chemistry, Drug discovery and development

## Abstract

The cool sensor transient receptor potential melastatin channel 8 (TRPM8) is highly expressed in trigeminal and dorsal root ganglia, playing a key role in cold hypersensitivity associated to different peripheral neuropathies. Moreover, these channels are aberrantly expressed in different cancers, and seem to participate in tumor progression, survival and invasion. Accordingly, the search for potent and selective TRPM8 modulators attracted great interest in recent years. We describe new heterocyclic TRPM8 antagonist chemotypes derived from *N*-cloroalkyl phenylalaninol-Phe conjugates. The cyclization of these conjugates afforded highly substituted β-lactams and/or 2-ketopiperazine (KP) derivatives, with regioselectivity depending on the *N*-chloroalkyl group and the configuration. These derivatives behave as TRPM8 antagonists in the Ca^2+^ microfluorometry assay, and confirmed electrophysiologically for the best enantiopure β-lactams **24a** and **29a** (IC_50_, 1.4 and 0.8 µM). Two putative binding sites by the pore zone, different from those found for typical agonists and antagonists, were identified by in silico studies for both β-lactams and KPs. β-Lactams **24a** and **29a** display antitumor activity in different human tumor cell lines (micromolar potencies, A549, HT29, PSN1), but correlation with TRPM8 expression could not be established. Additionally, compound **24a** significantly reduced cold allodynia in a mice model of oxaliplatin-induced peripheral neuropathy.

## Introduction

The transient receptor potential melastatin 8 (TRPM8) receptor is a multimodal channel, activated by cold and cooling compounds, such as menthol and icilin, but also by membrane depolarization and changes in extracellular osmolarity^1^. In the periphery, these channels are highly expressed in the afferent Aδ and C fibbers of sensory neurons, where they have been implicated in the perception and transduction of pain. Thus, cumulative evidence is signaling TRPM8 channels as pivotal players in cold hypersensitivity, especially that provoked by cancer chemotherapy^2–4^. Acute and chronic oxaliplatin-induced cold hypersensitivity has been reproduced in rats and correlated with TRPM8 expression and function^5,6^. Similarly, it is known that TRPM8 channels are implicated in inflammatory pain and migraine^7^. The thesis that migraine locus lies in the peripheral nervous system^8^, and the identification of TRPM8 as a candidate susceptibility gene for migraine^9,10^, points to the modulation of TRPM8 channels as a plausible mechanism for the treatment of this pathology^11^.

TRPM8 channels are also expressed in prostate, pancreas, and vascular, bronchopulmonary and urogenital tissues^12–16^. There are also numerous experimental evidences demonstrating that TRPM8 channels play important roles in tumor development and progression, including prostate, pancreas, breast, lung, colon, bladder and melanoma malignancies, among others^12,17^. In many cases, the aberrant expression of TRPM8 channels is correlated to tumor growth, progression and invasion capacity, at least in initial stages^18–21^, while sometimes TRPM8 channels are downregulated in final phases of the disease, and their activation seems to have a protective role^22^. It is also described that TRPM8 is expressed in the central nervous system, thus opening new opportunities to study and understand its potential role within the brain^23^.

Cryo-electron microscopy has demonstrated that the TRPM8 channel is a tetrameric protein, with 6TM helical segments (S1-S6) and intracellular *N*- and *C*-terminal domains, with the pore delimitated by a region situated between S5 and S6 helices^24^. The structure of TRPM8 in complex with some agonist compounds is disclosed, indicating a cavity for agonists delimited by S1, S4 and the TRP domain^25^. This highly adaptable pocket, delineated by the lower part of the S1–S4 transmembrane and the TRP domain, has also been described as the main cleft to bind AMTB and CT-I antagonists^26^.

**Figure 1:**
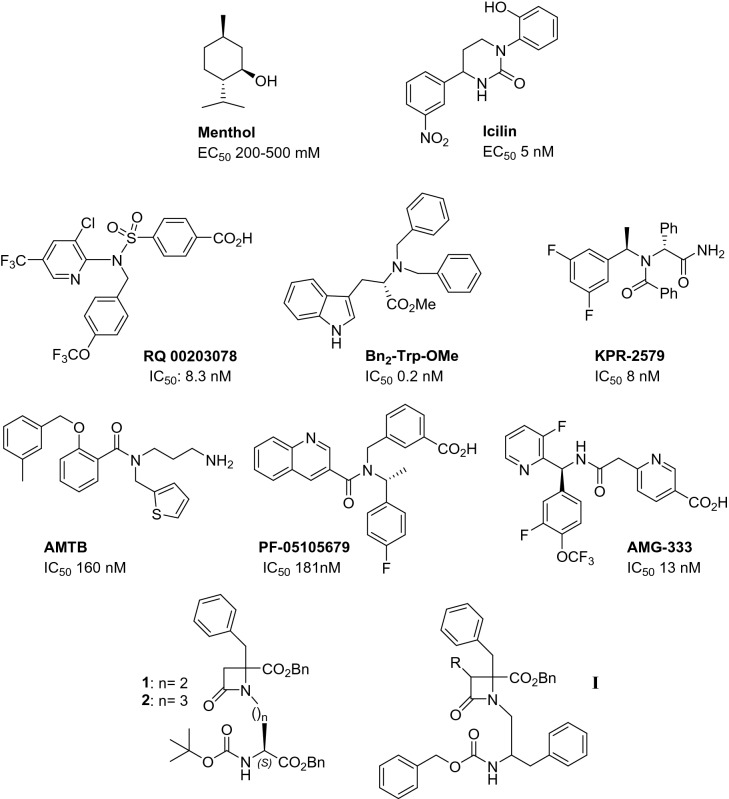
Representative TRPM8 modulators, β-lactams TRPM8 antagonists and general formula for phenylalaninol-derived analogues (**I**, this paper).

Because of the physiological and therapeutic significance of TRPM8 channels, many efforts have been devoted to the search for selective modulators, both agonists and antagonists^27–29^. Among the agonists, we can found menthol, diverse menthol derivatives, icilin, tertiary amides and different natural products^29–31^. As for the antagonists, most important chemotypes encompass different heterocyclic systems, including monocyclic central cores, like thiazole and β-lactam, and bicyclic structures, as benzothiophene, benzimidazole, and isoquinoline, among others^27,29,32^. Also different acyclic central scaffolds (amide, sulfonamide, urea, glycine, tryptophan), decorated with aromatic and heterocyclic rings are reported as TRPM8 antagonists (Fig. 1)^33–35^. Among the latter, AMG-333 and PF-05105679 reached phase I clinical trials for the oral treatment of cold induced pain and migraine, respectively^33,36^, but both studies were discontinued due to adverse secondary effects, including non-tolerated hot sensations. Therefore, there is still a need for TRPM8 antagonists with improved properties.

In this context, we have recently described a series of compounds derived from Phe and Asp/Glu amino acid conjugates and having a monocyclic β-lactam central core, which were able to potently and selectively inhibit the activation of TRPM8 by menthol, cool and voltage^37^. Among this series, the shorter Asp derivative **1** (n = 2, Fig. 1) was more potent than the longer Glu analogue **2** (n = 3, Fig. 1), while all the three benzyl and the Boc hydrophobic moieties are important for activity^37^.

Looking for shorter β-lactam derivatives, bearing four hydrophobic substituents, in this manuscript we describe the preparation of conjugates of Z-phenylalaninol with amino acid derivatives and their cyclization to heterocyclic compounds having a β-lactam or a 2-ketopiperazine central scaffold. Both series of compounds behave as TRPM8 antagonists and, among them, selected β-lactam derivatives display antitumor activity, and antiallodynic properties in a model of chemotherapy-induced cold allodynia.

## Results

### Design

The preparation of a shorter analogue of compounds **1** and **2** was initially projected starting from Boc-Ser-OBn (n = 1). However, all attempts to condense this Ser derivative with Ns-Phe-OBn, were unsuccessful due to the formation of the corresponding dehydroalanine analogue, as described in related reactions^38^. As the structure of TRPM8 channel was unknown at the moment of conceiving this work, we hypothesized that compounds of general formula **I** (Fig. 1), which maintain the β-lactam ring, its substitution at position 4, but change the Asp- or Glu-derived *N*-substituent by phenylalaninol-derived moieties, could constitute a new chemoptype for TRPM8 modulation. Although the changes are not bioisosteric, compounds **I** preserve two hydrophobic substituents on the *N*-appendage, which were demonstrated to be important for the activity of **1** and **2**.

### Chemistry

**Scheme 1:**
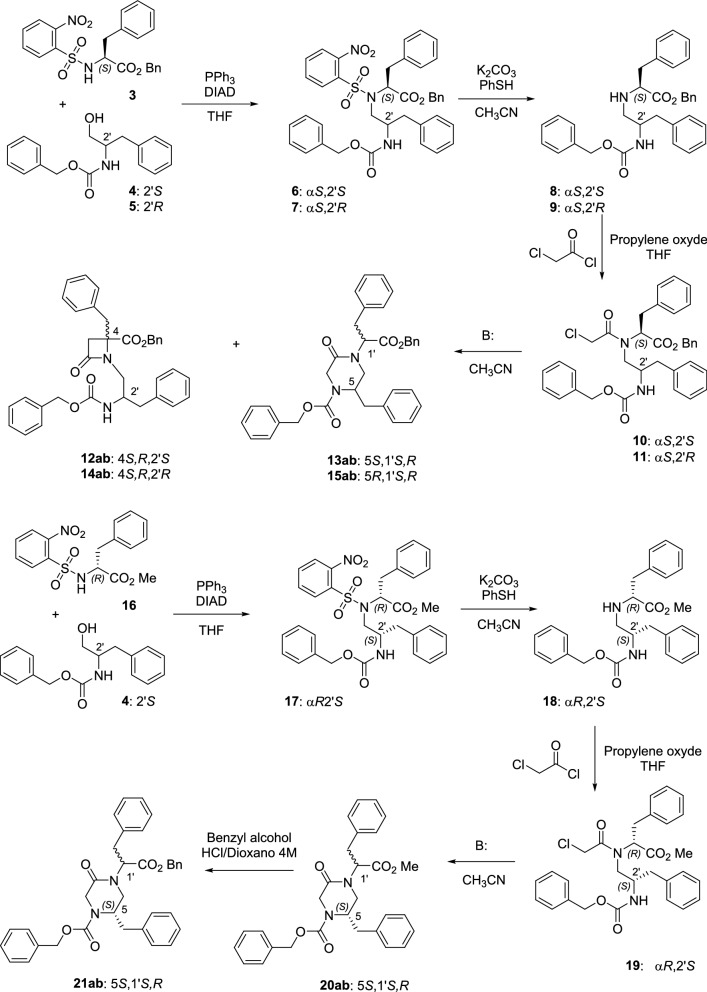
Preparation of chloroacetyl phenylalaninol-Phe conjugates and cyclization reactions.

As previously described for Phe-Asp conjugates^37^, the preparation of chloroacetyl derivatives **10** and **11** started by a Mitsunobu reaction between Ns-L-Phe-OBn (**3**) and the corresponding Z-phenylalaninol derivative (**4** or **5**) to give compounds **6** and **7** (Scheme 1). Then, the removal of the nosyl group afforded NH derivatives **8** and **9** that, finally, were reacted with chloroacetyl chloride to give key intermediates **10** and **11** (Scheme 1). The cyclization of these L-Phe-phenylalaninol conjugates in the presence of phosphazene bulky bases (BTPP, BEMP) led almost exclusively to the 2-ketopiperazine (KP) derivatives **13** and **15** (Scheme 1, Table 1), resulting from the cyclization through the NH group of the phenylalaninol-derived moiety. Under these conditions, formation of less than 3% of the expected β-lactams was observed in the HPLC chromatograms of the crude reaction mixtures. The ratio of β-lactams **12** and **14** increased slightly when the smaller Cs_2_CO_3_ base was employed in the cyclization, allowing their isolation in 8 and 11% yield, respectively (Table 1). The chloroacetyl phenylalaninol-D-Phe-OMe-derived conjugate **19**, despite bearing a smaller methyl ester that could favor the four-membered ring formation, exclusively led to the KP derivative **20** (Scheme 1, Table 1), independently of the base used. The benzyl ester analogue **21** was prepared by transesterification of **20**, in order to compare its activity with those of diastereoisomers **13** and **15**.

**Table 1:** Results of the cyclization reaction of chloroacetyl phenylalaninol-Phe conjugates.

Starting Compd	Base	Time (h)	β-L/KP ratio (HPLC)		β-Lactam# Yield (%)	Configuration (Isomer ratio)	KP # Yield (%)	Configuration (isomer ratio)
**10**	BTPP	5	3:97	NI		–	**13ab** (77)	5*S*,1′*S/*5*S*,1′*R* (80:20)
	BEMP	3	2:98	NI		–	NI	5*S*,1′*S/*5*S*,1′*R* (93:7)
	Cs_2_CO_3_	3	11:89	**12ab** (8.5)		4S,2′S/4R,2′S (83:17)	**13ab** (68)	5*S*,1′*S/*5*S*,1′*R* (95:5)
**11**	BTPP	5	2:98	NI		–	**15ab** (77)	5*R*,1′*S/*5*R*,1′*R* (81:19)
	BEMP	6	2:98	NI		–	NI	5*R*,1′*S/*5*R*,1′*R* (80:20)
	Cs_2_CO_3_	6	27:73	**14ab** (11)		4*S*,2′*R/*4*R*,2′*R* (88:12)	**15ab** (69)	5*R*,1′*S/*5*R*,1′*R* (90:10)
**19**	BTPP	5	0:100	–			NI	5*S*,1′*S/*5*S*,1′*R* (40:60)
	Cs_2_CO_3_	6	0:100	–		–	**20ab** (60)	5*S*,1′*S/*5*S*,1′*R* (4:96)
**20ab**	–	–	–	–		–	**21ab** (55)	5*S*,1′*S/*5*S*,1′*R* (10:90)
**36**	BTPP	5	0:100	–		–	**38ab** (81)	5*R*,1′*S/*5*R*,1′*R* (86:14)

*NI* not isolated.

All these KP derivatives were obtained as mixtures of two diastereoisomers at C1′ in variable proportions (Table 1). The configuration was indirectly assigned by the preparation of Ala dipeptide derivatives from **13ab** (see supplememntary information for details), and applying the known rule of differential HPLC retention times and chemical shifts of the Ala CH_3_ group between homochiral and heterochiral dipeptide derivatives^39,40^.

β-Lactam derivatives **12** and **14** were also formed as mixtures of two diastereoisomers at C4. Considering that the memory of chirality favors the formation of 4*S* isomers when starting from L-Phe^39,41,42^, the configuration of the major diastereosiomer was assigned as 4*S*.

**Scheme 2:**
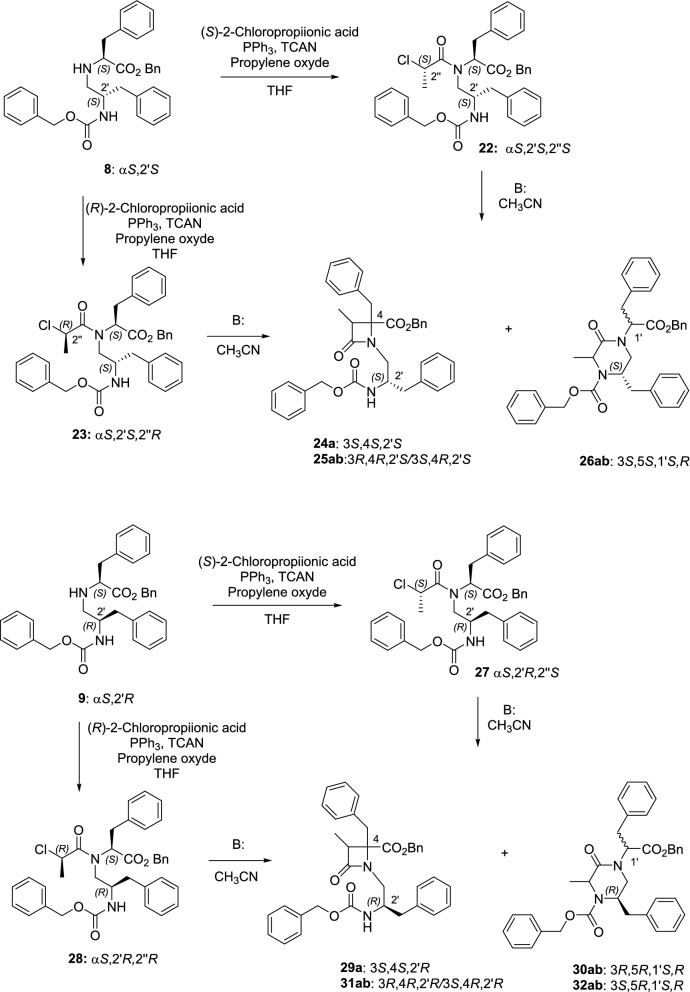
Preparation of 2-chloropropanoyl phenylalaninol-Phe conjugates and cyclization reactions.

To attempt to obtain β-lactams as single isomers, we prepare enantiopure 2-chloropropanoyl Z-phenylalaninol-Phe-OBn conjugates **22**, **23**, **27** and **28** (Scheme 2). For this, conjugates **8** and **9** were reacted with 2*S*- or 2*R*-chloropropionic acid in the presence of trichloroacetonitrile and triphenylphosphine. We have previously demonstrated that related 2-chloropropanoyl derivatives afforded pure β-lactams, with the stereochemistry at C3, C4 dictated by the configuration of the chloropropionic moiety^43^. In good agreement with our precedents, cyclization of the all *S* isomer **22** with BTPP led almost exclusively to the formation of the 3*S*,4*S*,2´*S* β-lactam **24a** (Table 2), along with less than 11% of the corresponding KP (not isolated). Again, the percentage of conversion to the four-membered ring was higher when Cs_2_CO_3_ was used as base (Table 2). Similarly, the basic treatment of the 2′*R*,2″*S*-chloropropanoyl derivative **27** with BTPP afforded enatiopure 3*S*,4*S*,2′*R* 2-azetidinone **29a**. However, in this case, the indicated β-lactam was obtained along with about 50% of the corresponding KP **30ab** (**a**:**b**, 81:18). Cyclization of **27** with Cs_2_CO_3_ afforded a mixture of β-lactam and KP in the same ratio (48:52), but in this case the 2-azetidinone derivative was obtained as a mixture of two diastereoisomers (**29ab**, 73:27, see SI for a possible explanation). The KP derivative **26ab** was the main reaction product (> 85%) during the treatment of the 2′*S*,2″*R*-chloropropanoyl precursor **23**, and the minor β-lactam was also obtained as a diastereomeric mixture (**25ab**, Table 2). Contrastingly, the 2-azetidinone derivative **31ab** was the main product (> 70%) after the cyclization of the 2′*R*,2″*R*-chloropropanoyl precursor **28**. Therefore, the configuration of the linear precursor influenced both the regioselectivity (β-lactam versus KP) and the stereoselectivity in the formation of β-lactam derivatives.

**Table 2:** Results of the cyclization reaction of 2-cloropropanoyl phenylalaninol conjugates.

Starting Compd	Base	Time (h)	β-L/KP ratio (HPLC)	β-Lactam# Yield (%)	Configuration (Isomer ratio)	KP # Yield (%)	Configuration (Isomer ratio)
**22**	BTPP	4	89:11	**24a** (65)	3*S*,4*S*,2′*S*	NI	−
	Cs_2_CO_3_	24	93:7	**24a** (70)	3*S*,4*S*,2′*S*	NI	−
**23**	BTPP	4	14:86	**25ab** (10)	3*R*,4*R*,2′*S/*3*S*,4*R*,2′*S* (85:15)	**26ab** (63)	3*S*,5*S*,1′*S*/3*S*,5*S*,1′*R* (81:19)
	Cs_2_CO_3_	336	11:89	NI	3*R*,4*R*,2′*S/*3*S*,4*R*,2′*S* (85:15)	NI	3*S*,5*S*,1′*S*/3*S*,5*S*,1′*R* (85:15)
**27**	BTPP	5	48:52	**29a** (39)	3*S*,4*S*,2′*R*	**30ab** (32)	3*R*,5*R*,1′*S*/3*R*,5*R*,1′*R* (82:18)
	Cs_2_CO_3_	168	48:52	NI	3*S*,4*S*,2′*R/*3*R*,4*S*,2′*R* (73:27)	NI	3*R*,5*R*,1′*S*/3*R*,5*R*,1′*R* (65:35)
**28**	BTPP	56	72:28	**31ab** (44)	3*R*,4*R*,2′*R/*3*S*,4*R*,2′*R* (77:23)	**32ab** (21)	3*S*,5*R*,1′*S*/3*S*,5*R*,1′*R* (85:15)
	Cs_2_CO_3_	336	82:18	**31ab** (59)	3*R*,4*R*,2′*R/*3*S*,4*R*,2′*R* (78:22)	NI	3*S*,5*R*,1′*S*/3*S*,5*R*,1′*R* (96:4)
**37**	BTPP	6	42:58	**39a** (30)	3*S*,4*S*,2′*R*	**40ab** (41)	3*R*,5*R*,1′*S*/3*R*,5*R*,1′*R* (58:42)

NI: not isolated.

A similar reactivity was observed during the cyclization of Ala derivatives (Supplementary Scheme S1). Accordingly, treatment with BTPP of the chloroacetyl derivative **36** afforded exclusively the 6-membered KP **38ab** (**a**:**b**, 86:14, Table 1), while chloropropanoyl analogue **37** led to a 42:58 mixture of the 2-azetidinone derivative **39a** (single isomer, 3*S*,4*S*,2′*R* , Table 2) and the KP **40ab** (**a**:**b**, 58:42).

### TRPM8 in vitro activity

The ability to inhibit menthol-induced Ca^2+^ intracellular influx into the cytosol on HEK293 cells heterologously expressing the rat TRPM8 channel was measured and compared to that of AMTB, a well-known TRPM8 antagonist. The results obtained for β-lactam and KP derivatives are depicted in Table 3. Representative recordings of fluorescence obtained in microfluorometry experiments for selected compounds are in Supplementary Fig. S3. No agonist activity was observed for these compounds in the absence of menthol.

**Table 3:** Activity at TRPM8 of β-lactams derived from phenylalaninol conjugates.

Compd	Isomers	Isomers ratio	% Blockade 50 µM	% Blockade 5 µM	IC_50_ (μM)
**β-Lactams**					
**12ab**	4*S*,2′*S/*4*R*,2′*S*	83:17	100.0 ± 3.1	92.3 ± 6.2	1.5 ± 1.2
**14ab**	4*S*,2′*R/*4*R*,2′*R*	88:12	103 ± 2.7	91.0 ± 4.3	1.2 ± 1.0
**24a**	3*S*,4*S*,2′*S*	−	110.7 ± 11.7	65.4 ± 6.4	2.4 ± 1.2
**25ab**	3*R*,4*R*,2′*S/*3*S*,4*R*,2′*S*	85:15	89.2 ± 9.2	68.2 ± 5.6	3.9 ± 1.9
**29a**	3*S*,4*S*,2′*R*	−	100.0 ± 5.0	97.2 ± 4.8	0.4 ± 1.5
**31ab**	3*R*,4*R*,2′*R/*3*S*,4*R*,2′*R*	77:23	105.0 ± 5.5	91.0 ± 4.3	1.6 ± 1.3
**39a**	3*S*,4*S*,2′*R*	−	100.4 ± 3.5	46.6 ± 11.1	6.2 ± 1.1
**2-Ketopiperazines**					
**13ab**	5*S*,1′*S*/5*S*,1′*R*	80:20	102.1 ± 7.7	48.9 ± 6.2	17,9 ± 1.3
**15ab**	5*R*,1′*S*/5*R*,1′*R*	81:19	106 ± 5.06	101.4 ± 6.1	1.8 ± 1.9
**20ab**	5*S*,1′*S*/5*S*,1′*R*	4:96	61.0 ± 5.6	22.3 ± 5.5	18.8 ± 1.7
**21ab**	5*S*,1′*S*/5*S*,1′*R*	10:90	92.5 ± 4.3	70.0 ± 6.8	2.0 ± 1.5
**26ab**	3*S*,5*S*,1′*S*/3*S*,5*S*,1′*R*	81:19	102.4 ± 1.5	93.5 ± 3.4	2,4 ± 1,3
**30ab**	3*R*,5*R*,1′*S*/3*R*,5*R*,1′*R*	82:18	101.2 ± 11.4	101.7 ± 5.3	0.16 ± 1.6
**32ab**	3*S*,5*R*,1′*S*/3*S*,5*R*,1′*R*	96:4	115.1 ± 4.5	47.4 ± 6.8	17.4 ± 1.6
**38ab**	5*R*,1′*S*/5*R*,1′*R*	86:14	105.5 ± 3.2	66.0 ± 3.3	2.5 ± 1.3
**40ab**	3*R*,5*R*,1′*S*/3*R*,5*R*,1′*R*	58:42	107.9 ± 2.5	79.1 ± 3.3	0.8 ± 1.2
**AMTB**					7.3 ± 1.5

As shown in Table 3, slightly better antagonist activity was observed for β-lactam with an *N*-2′*R*-appendage (compare 2′*R*-derivatives **14ab**, **29a** and **31ab** to 2′*S*-analogues **12ab**, **24a** and **25ab**, respectively). However, in the case of distereoisomeric mixtures (**ab**), the exact contribution to the activity of each individual isomer cannot be assessed. As previously described for the first generation of β-lactam TRPM8 antagonists^37^, the phenyl group at position 4 is important for Ca^2+^ entrance inhibition, since the 4-CH_3_ derivative **39a** was one order of magnitude less active than the 4-Bn analogue **29a**.

All KP derivatives were assayed as mixtures of two diastereoisomers, therefore the structure–activity relationships should be considered as tendencies, not as absolute statements. As for the β-lactam derivatives, the configuration of the stereocenter coming from the phenylalaninol moiety seems to dictate the antagonist activity, with 5*R*-KPs more potent than 5*S*-isomers (compare **15ab** to **13ab**) (Table 3). The 1′-configuration appears also to play a role for the inhibition of menthol-induced TRPM8 activation, with a preference for the 1′*R*- (**21ab**, 10:90) over the 1′*S*-isomer (**13ab**, 80:20). The OBn group in **21ab** could participate in the direct interaction with the TRPM8 channel, as the corresponding OMe analogue **20ab** shows an important drop in activity. In the 3-methyl derivatives, a 3*R*,5*R* configuration (in **30ab**) is preferred over the 3*S*,5*S* combination (in **26ab**), while the 3*S*,5*R*-configured diastereoiomers (**32ab**) showed the lowest activity in this series. In this case, a 4-CH_3_ group led to slightly less active derivatives (**38ab**, **40ab**) compared to the corresponding 4-Bn analogues (**15ab** and **30ab**, respectively), although the fall in activity due to this modification is less acute than in the β-lactam series.

β-Lactam and 2-ketopiperazine derivatives were also assayed for their activity in cell expressing hTRPV1 channels. No significant antagonist activity was measured for any derivatives within both chemotypes (Supplementary Tables S1, S2, and Fig. S3), indicating their selectivity for TRPM8 channels.

The TRPM8 antagonist activity of the enantiopure β-lactams **24a** and **29a** was further confirmed electrophysiologically by Patch-clamp experiments, using the whole cell configuration in HEK293 cells expressing TRPM8 channels.

**Figure 2:**
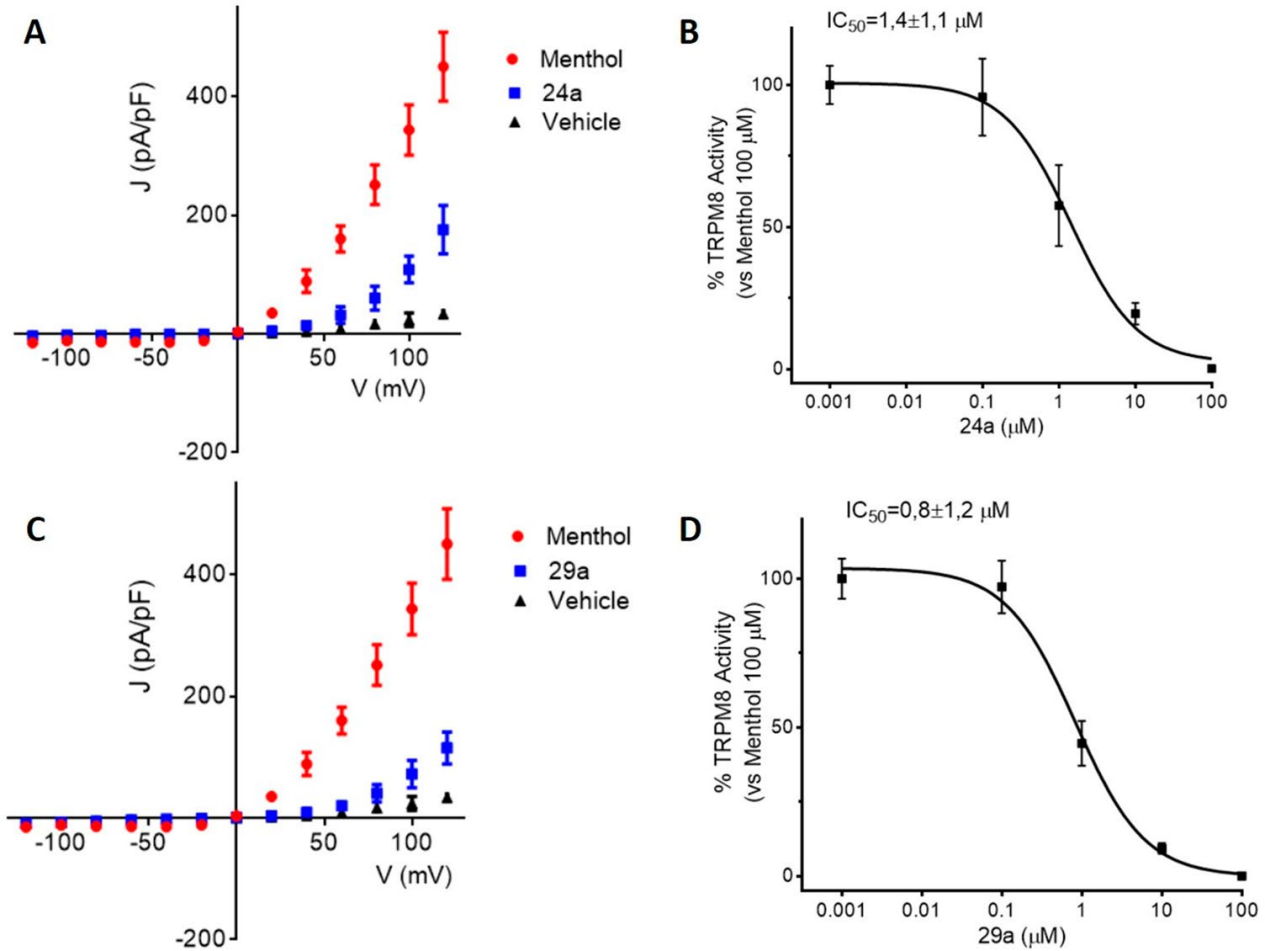
Compounds **24a** and **29a** block TRPM8-mediated responses evoked by menthol in rTRPM8-expressing HEK293 cells. (**A**, **C**). *I–V* curves obtained in HEK293 cells expressing TRPM8 and exposed to vehicle solution (Vehicle; black trace; **A**,**C**), 100 µM menthol (red trace; **A**,**C**), 100 µM menthol + 10 µM **24a** (blue trace; **A**) or to 100 µM menthol + 10 µM compound **29a** (blue trace; **C**) (**B**,**D**), Concentration − response curves for TRPM8 current blockade by compound **24a** (B) or compound **29a** (D) at a holding voltage of -60 mV. Peak current data were expressed as pA/pF (to facilitate comparison among cells of different size) and expressed as a function of antagonist concentrations. The solid lines represent fits of the experimental data to the following binding isotherm: y = max/(1 + x/EC_50_)n, where x is the drug concentration and n the Hill coefficient. The fitted values for n were 0.97 ± 0.05 or 0.98 ± 0.6 for compound **24a** or **29a**, respectively. Each point is the mean ± SD of 8 (for compound **24a**) or 9 (for compound **29a**) determinations, each obtained in different cells.

As shown in Fig. 2, perfusion with 100 µM menthol gives rise to a strongly outward rectifying ionic current characterized by the presence of negligible current at negative potential and the presence of a linear current increase (ohmic) at positive voltages ≥ 40 mV (I-50 mV/I + 120 mV = 0.07). When 10 µM of **24a** was applied (Fig. 2A, blue), an important reduction on the menthol-mediated TRPM8 activity at depolarizing voltages (+ 120 mV) was observed. A similar behaviour was detected for diastereomeric β-lactam **29a** (Fig. 2C, blue). The dose–response curve for both compounds was obtained at a holding potential of -60 mV (Supplementary Fig. S4). The IC_50_ values were 1.4 ± 1.1 µM for **24a** (Fig. 2B) and 0.8 ± 1.1 µM for **29a** (Fig. 2D).

### Docking studies

In order to investigate possible binding pockets within the TRPM8 channel for these families of KP and β-lactam TRPM8 antagonists, we performed computational studies with compounds **13a**, **24a**, and **29a**. A model of the rat TRPM8 channel, created from the cryo-electron microscopy structure of the *Ficedula albicollis* (PDB code 6BPQ)^24^, was used, and docking simulations were performed with the software implemented in Yasara^44–46^.

These docking studies predicted that the three compounds most likely (> 80% solutions) interact with the TRPM8 by the pore zone, with two major solutions having the best binding energies (Supplementary Fig. S5, Table S3). Site 1 was identified in the middle of the transmembrane region, mainly involving TM5 (S5) and TM6 (S6) of one monomer and segments of an adjacent subunit (S5 or S6 and/or the S5-S6 segment forming the pore). The second binding compartment, Site 2, correspond to the cytosolic mouth of the pore, involving the loops connecting TM6 and TRP domains of the 4 protein subunits forming the channel. As for the compounds, mainly hydrophobic interactions can be distinguished at both binding pockets, with some π–π stacking and a few H-bonds identified.

Ketopiperazine **13a** binds TRPM8 channel at Site 1 through two π–π stacking connections, a face-to-face stacked interaction between the phenyl group of the 1′-Bzl moiety and Y963 at subunit 1 (S6), and secondly a T-shaped (edge-to-face) contact encompassing the phenyl group of the α-CO_2_Bzl moiety and F874 residue of TRPM8 subunit 3 (S5) (Supplementary Fig. S6). Among hydrophobic interactions, the Ph group of the Cbz moiety occupies a hydrophobic pocket delineated by residues located at subunit 3 (F870, L873, F874, I962, L965, and I969) and subunit 2 (I844). In addition, the 1′-Bn moiety is also involved in hydrophobic interactions through L871 and F874 (Subunit 3), while the phenyl group of the 5-Bn is in touch with I962 and Y963, also at subunit 3. At Site 2, a face-to-face π–π stacking involves the Ph group of the Cbz and the aromatic ring of the Y981 residue (subunit 1). Moreover, the hydrophobic interactions comprise the four Ph rings of **13a** and residues from the four channel subunits (see Supplementary Fig. S7).

**Figure 3:**
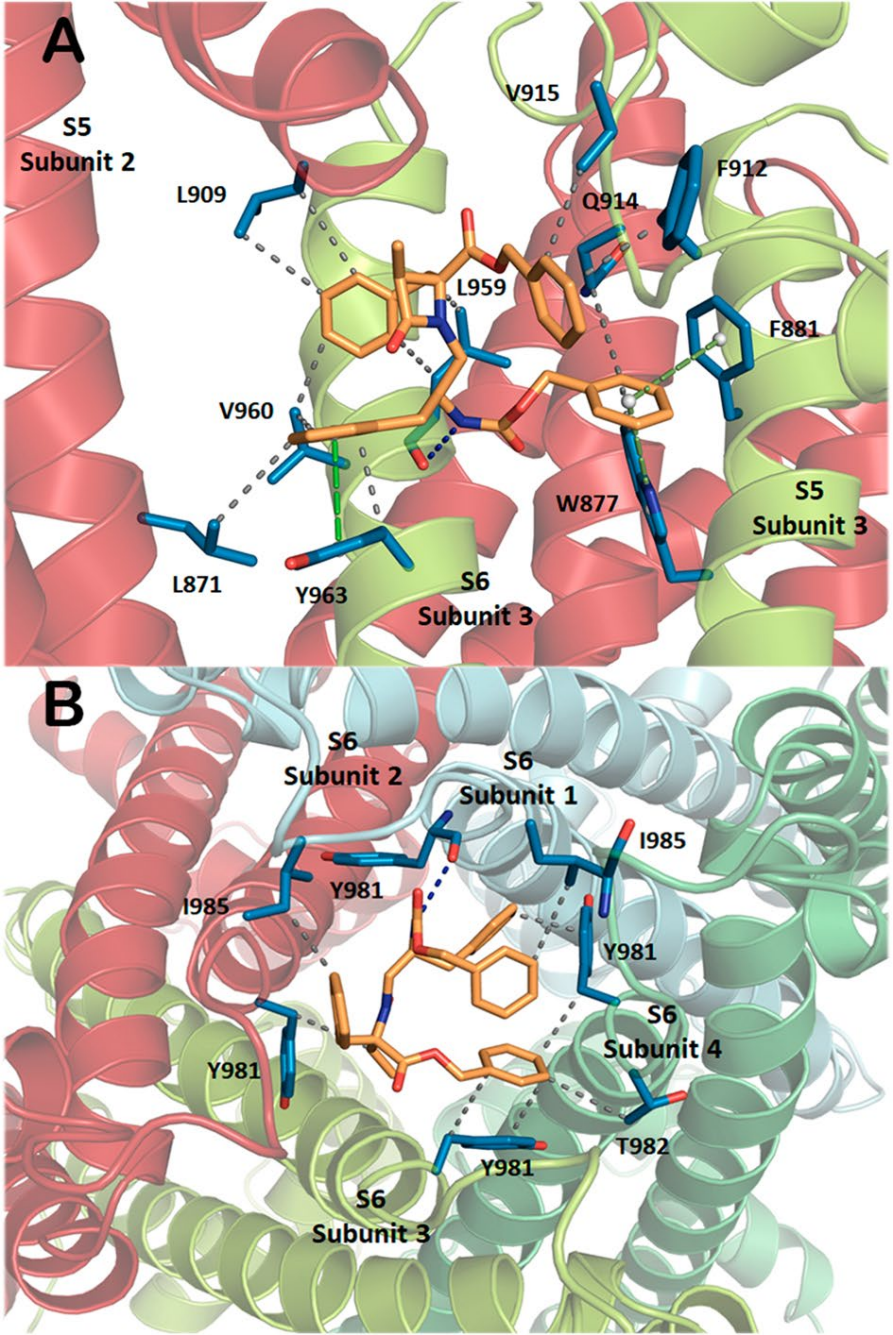
Low energy binding sites for β-lactam derivative **24a**, Site 1 (**A**), Site 2 (**B**). Compound **24a** is in pale orange, while side-chains of TRPM8 involved in the interaction are depicted in blue and labelled. Heteroatoms are indicated in red (O) and dark blue (N). H atoms have been removed.

**Figure 4:**
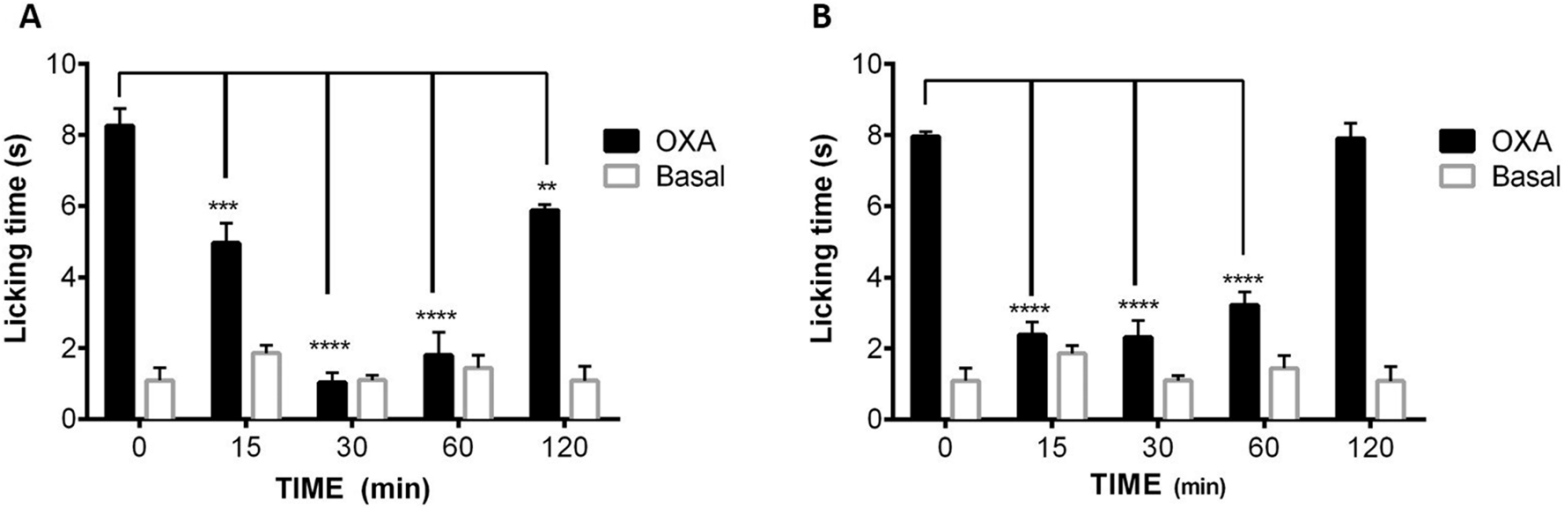
Effects of compound **24a** on the oxaliplatin-induced cold allodynia (acetone test). Male mice were treated with oxaliplatin (6 mg/Kg, ip) or vehicle on days 1, 3 and 5. Then, compound **24a** (1 µg/ipl, **A** or 3 µg/ipl, **B**) was administered to the oxaliplatin treated animals, and the time-course of cold allodynia was measured. Data are given ± SME (n = 5). **P < 0.05; ***P < 0.001; ****P < 0.0001.

At Site 1, β-Lactams **24a** and **29a** occupy similar areas of the transmembrane region, involving two neighboring channel subunits, but their poses are clearly different, with the β-lactam ring pointing to the upper and lower part of the binding pocket, respectively (Fig. 3 and Supplementary Figs. S8–S11). The 3*S*,4*S*,2′S isomer **24a** binds the channel through a H-bond involving its NH and the backbone CO of L959 (S5, subunit 3) (Fig. 4 and Supplementary Fig. S8). Additionally, three π-π interactions contribute to the complex stabilization, two T-shaped contacts comprising the Ph group of the Cbz moiety and W877 and F881 side-chains (both at S5), and a face-to-face sandwich between the aromatic groups of 2′-Bn and Y963 residue (S6), all at channel monomer 3. This monomer contributes also to the complex with nine hydrophobic interactions among a series of residue side-chains of the channel and the four Ph groups of the antagonist, while subunit 2 (S5 and S5-S6 loop) add three additional hydrophobic contacts (specified in Supplementary Fig. S8). The diastereoisomeric 3*S*,4*S*,2′*R* compounds **29a** is fixed to the channel through a H-bond (NH → CO of G913, S5-S6 segment, subunit 1) and a parallel displaced π–π connection (OBn-Y963 of S6, subunit 1, Supplementary Fig. S10). Also, a number of residues at subunit 1 provide hydrophobic interactions with the four Ph groups of **29a**. As previously, the contiguous monomer (subunit 4) also supplies additional stabilizing interactions, in this case involving three residues at the pore-forming S5-S6 segment (Supplementary Fig. S10).

Main residues involved in the hydrophobic interactions of **24a** and **29a** at Site 2 are Y981 (from three out of four subunits), T982 of monomer 4, and I985 of two subunits. In addition, a H-bond between the CO group of Y981 (subunit 1, S6) and the NH group of **24a**, and a π–π displaced stacking interaction involving the phenyl groups of the 2′-Bn moiety in **29a** and Y981 side-chain (Subunit 1, S6), contribute to the respective stabilization of the complexes at Site 2 (Fig. 4, and Supplementary Figs. S9 and S11). Interestingly, most residues of rTRPM8 suggested as important for the interaction with β-lactams and KPs at both sites 1 and 2 are highly conserved in hTRPM8 (Supplementary Fig. S12).

### Growth inhibitory activity in tumor cells

A number of recent experimental evidences position TRPM channels as important players in cancer growth and progression^47^. Among these channels, the aberrant expression of the TRPM8 subtype has been described in different human malignant tumors, including those of prostate, pancreas, breast, colon, and skin, among others^3^. More importantly, sometimes the TRPM8 overexpression was associated to poor prognosis of cancer patients. In good agreement, several, structurally different TRPM8 antagonists demonstrated good antitumor activity in prostate^32,48,49^, and others human tumor cell lines^50^. A few years ago we have described that Phe-derived simpler β-lactams showed antitumor activity against three human cancer cell lines^51^. Based on these precedents, in this work we first aimed at evaluating the antitumor activity of enantiopure compounds **24a** and **29a**. Initially, we assessed the growth inhibition percentage by a 10 µM concentration of these compounds on a 60 tumor cell screen at the National Cancer Institute (USA). In general, both β-lactam derivatives showed non-selective, modest cytotoxic effects in all cell assays, with the best data for leukemia (MOLT-4 cell line), melanoma (SK-MEL-5), lung (A549), colon (COLO-5), ovarian (OVCAR-4), renal (A498) and prostate (PC-3) cancer lines.

We then measured the in vitro cytotoxic activity of **24a** and **29a** in four human tumor cell lines, namely A459 (lung), HT29 (colon), MDA-MB-231 (breast) and PSN1 (pancreas). The results are recorded in Tables 4 and S4, and compared to those of the well-known chemotherapeutic agent doxorubicin. As shown in Table 4, compound **24a** displays in vitro cytotoxic activity in the micromolar range in three out of four assayed tumor cell lines, with no activity against the MDA-MB-231 breast cell line (at 10 µM/mL). Compared to **24a**, slightly lower potencies were measured for β-lactam **29a** in lung, colon and pancreas tumor cell lines, but contrastingly it displays better, although moderate, in vitro cytotoxic activity in the breast cell line. Hence, no significant influence neither of the configuration of the β-lactam derivative nor of the TRPM8 antagonist potency was observed on the antiproliferative activity of these compounds. In general, these activities were one order of magnitude less potent than the control doxorubicin.

**Table 4:** In vitro cytotoxicity (GI_50_, μM) of compounds **24a** and **29a** on four human cell cancer lines.

Compd.	Family	TRPM8 IC_50_ (μM)	Lung-NSCLC	Colon	Breast	Pancreas
			A549	HT29	MDA-MB-231	PSN1
**24a**	β-Lactam	2.4 ± 1.2	3.29	4.16	> 17.3	5.55
**29a**	β-Lactam	0.4 ± 1.5	5.90	7.11	12.7	6.42
**13ab**	2-KP	17,9 ± 1.3	> 17.8	> 17.8	> 17.8	> 17.8
**15ab**	2-KP	1.8 ± 1.9	> 17.8	> 17.8	> 17.8	6.4
**30ab**	2-KP	0.16 ± 1.6	12.7	> 17.3	> 17.3	8.3
**Doxorubicin.HCl**	−		0.24	0.19	0.17	0.17

Three steroisomeric 2-KP derivatives, having different TRPM8 antagonist capacity, were also assayed for their antitumoral activity (Table 4). In this case, compound **30ab**, showing submicromolar TRPM8 antagonist activity, is only moderately active in A549 and PNS1 cell lines, with no significant cytotoxicity in the colon and breast cell lines. Compound **15ab**, with micromolar potency as TRPM8 antagonist, is only cytotoxic in the pancreas cell line, while the less potent analogue **13ab** did not show any significant antiproliferative activity. It seems that for the 2-KP series the in vitro cytotoxicity follows the same order than TRPM8 antagonist potency. The lower antitumor potential, compared to β-lactams, could be due either to the evaluation of diasteroisomeric mixtures in 2-KPs versus enantiopure β-lactams, or to the fact that the cytotoxicity of β-lactam derivatives is independent of the TRPM8 activity or both.

The expression of TRPM8 channels in different cancer cell lines has been scarcely studied^52^. To the best of our knowledge, there are no data on expression levels in A549, HT29 and PSN1 tumor cell lines, while for MDA-MB-231 the results described so far are contradictory. Thus, while the TRPM8 antagonist AMTB decreases viable cells in MDA-MB-231 breast cancer cells and TRPM8 levels are high in basal breast cancers, the TRPM8 expression does not seem very high in this and other breast cancer cell lines^53^. On the contrary, a work by Liu and coworkers identified high levels of channel expression in different breast cancer cell lines, including MDA-MB-231^54^. Because this lack of information and the controversial results, we cannot assure that the antitumor activity displayed by our compounds is partially due to a high expression of TRPM8 in the indicated cell lines or if it could be an effect totally independent of these channels.

No apparent, significant cytotoxic effects were observed for the β-lactam derivatives in HEK293 cells (up to 500 µM concentration, MTT assay).

### Antiallodynic effects in vivo

Cold allodynia (painful sensation at cold temperatures that do not usually cause pain) and cold hyperalgesia (increased sensitivity to distressing cold temperatures) are associated to different peripheral neuropathies^55^. Several chemotherapeutic agents in first clinical line induce peripheral neuropathies (known as CIPN), affecting million patients worldwide and limiting the dose administered to them, as well as the quality of life of many survivors^56^. In oxaliplatin CIPN, the increased sensitivity to cold has been correlated to an augmented expression of TRPM8 channels, among others^4–6,57^. In good correlation, there are recent experimental evidences describing that TRPM8 antagonists are able to decrease oxaliplatin-induced allodynia and cold hypersensitivity^34,58^. According to these discoveries, we decided to explore the effects of β-lactam **24a** in an in vivo model of oxaliplatin CIPN, using acetone assay for monitoring cold allodynia. In male mice, the injection of oxaliplatin on days 1, 3 and 5 at a 6 mg/kg dose produces peripheral cold allodynia. As shown in Fig. 4, the intraplantar (i.pl.) administration of β-lactam derivative **24a** (1 μg), attenuates the cold-induced paw licking in a significant manner 15 min after administration, showing the maximum activity from 30 to 60 min. At a 3 µg dose, the antagonist activity is clearly increased at 15 min, and firmly maintained up to 60 min.

## Discussion

To search for new TRPM8 antagonist chemotypes, we explore the base-assisted cyclization of linear phenylalaninol-Phe conjugates, which afforded chiral β-lactam and/or 2-ketopiperazine (KP) heterocyclic derivatives. The regioselectivity (β-lactam *versus* KP) was dependent on the chloroalkyl substituent and the configuration of the linear precursor. While 2-chloroacetyl derivatives gave almost exclusively to the KP six-membered ring heterocycle, the cyclization of 2-chloropropanoyl analogues is governed by the configuration of both the phenylalaninol-derived (2′) and the 2-chloropropanoyl (2″) stereocenters. In short, 2′*S*,2″S and 2′*R*,2″*R* isomers provides β-lactams as the very major component of the reaction, while the KP heterocycle predominates after the cyclization of the 2′*S*,2″*R* diastereoisomer, and the 2′*S*,2″*R* linear precursor provides almost the same amount of the four- and six-membered heterocyclic systems. The epimerization at the C-3 stereocenter in 3-methyl-β-lactam derivatives, not previously observed for related 2-azetidinones^43^, was low for 3*S*,4*S*-configured compounds and more important for 3*R*,4*R*-analogues.

Both, the phenylalaninol-Phe-derived β-lactams and KPs behave as new TRPM8 antagonist chemotypes, blocking the channel activation by menthol (Ca^2+^ entry assay) with micromolar or submicromolar potencies, and did not show activity at hTRPV1. Single isomer β-lactams **24a** and **29a** display IC_50_ values of 2.4 and 0.4 µM, respectively, indicating that a 2′*R*-configuration of the phenylalaninol-derived substituent is preferred for TRPM8 antagonist activity. These antagonist activities were further confirmed using electrophysiology experiments, with Patch-clamp measurements sustaining that the 2′*R* diastereoisomer is slightly more potent than the corresponding 2′*S* isomer. In general, these phenylalaninol-Phe-derived β-lactams maintain significant TRPM8 blockade activity, although they showed somewhat decreased potency compared to the longer Asp-Phe analogues^37^. For KPs, a 1′*R*- and a 5*R*-configuration seem to favor the inhibition of TRPM8 channel activation.

Docking studies, using a homology model of rat TRPM8 channel, built on the cryo-electron microscopy structure of the TRPM8 from *Ficedula albicollis*^24^, propose two putative binding sites, by the pore zone, for the phenylalaninol-Phe-derived heterocyclic compounds described here. The first site involves transmembrane S5 and S6 of one channel subunit and the S5 or S6 and/or the S5–S6 segment forming the pore of one adjacent monomer, suggesting an allosteric modulation of the channel. The second most probable binding point is located at the bottom part of the pore, involving mainly hydrophobic interaction among the phenyl rings of the molecules and hydrophobic and aromatic residues of the four channel subunits, with the compound acting as a channel blocker. The sites predicted by these models of interaction differs from those proposed for tryptophan-derived antagonists^33^, and that from AMTB and TC-I/TRPM8 complexes solved by cryo-electron microscopy^26^, which adopt different poses within the channel, but all around the menthol-binding pocket (delineated by the lower half of the TM4-TM5 helices and the TRP domain)^25^. The larger volume of our molecules could be behind this different behaviour.

TRPM8 channels are overexpressed in a number of tumors, like prostate, melanoma, lung and colon adenocarcinomes, and some TRPM8 antagonists demonstrated good antitumor activity^32,48,49^. Interestingly, enantiopure β-lactams **24a** and **29a** exhibited non-selective antitumor activity in different tumor cell lines, showing micromolar potency in four of them, while 2-KP regioisomeric compounds displayed lower antifloliferative activity. No direct correlation between TRPM8 antagonist and antitumor activity could be established. Abnormalities in TRPM8 expression was also found in models of chemotherapy-induced peripheral neuropathy^57^. To evaluate the usefulness of this family of compounds in relieving persistent pain associated to antineoplastic agents, we evaluated β-lactam **24a** in a mice model of oxaliplatin-induced peripheral neuropathy. Compound **24a** demonstrated significant antiallodynic effects in vivo, with maximum activity from 30 to 60 min. Compared to literature reported results, the antiallodynic activity of compound **24a** seems superior or comparable to that of other described TRPM8 antagonists. Thus, its potency is higher than that of a described spirochromene derivative ^59^, and seems to span longer than that of a Trp-OMe derivative, which showed more potent antagonist activity at the Ca^2+^ assay than **24a**^34^. At 1 µg i.pl., β-lactam derivative **24a** showed slightly lower potency and similar duration of action than a biphenyl amide TRPM8 antagonist recently reported^60^.

In conclusion, the two phenylalaninol-Phe-derived heterocyclic systems, highly functionalized β-lactam and/or 2-ketopiperazine, allow the identification of new hits for TRPM8 modulation. Therefore, these two new chemotypes could constitute the starting point for further modifications on the road to improved compounds for future therapeutic applications in both pain and cancer.

## Experimental section

### Chemistry

Preparation of synthetic intermediates, their characterization and that of most final compounds are detailed in Supplementary Information.

### Cyclization reactions

BTPP (2.4 mmol, 0.75 mL), or BEMP (2.4 mmol, 0.69 mL) or Cs_2_CO_3_ (3.2 mmol, 1.04 g) was added to a solution of the corresponding *N*-alkyl-*N*-chloroacetyl- or *N*-alkyl-*N*-chloropropanoyl-Xaa derivative (1.6 mmol) in dry CH_3_CN (4 mL), under Ar atmosphere. The reaction mixture was stirred until consumption of the starting material. Then, the solvent was removed and the residue was extracted with EtOAc, and washed with 0.1 M HCl, H_2_O and brine, successively. Finally, the organic phase was dried over Na_2_SO_4_, filtered, and concentrated. The resulting residue was purified by flash chromatography on silica gel, using the eluent indicated in each case.

### 4*S*-Benzyl-4-benzyloxycarbonyl-3*S*-methyl-1-[(2′*S*-benzyloxycarbonylamino-3′-phenyl)prop-1′-yl]-2-oxoazetidine (24a)

Syrup. Yield: 65% (from 22, B: BTPP). Eluent: EtOAc:Hexane (1:2). HPLC: t_R_ = 16.20 min (gradient of 30% to 95% of A, in 20 min).[α]_D_ = -35.04 (c 1, CHCl_3_). Isomer ratio M(3*S*,4*S,2′S*):m(3*R*,4*S,2′S*) = 97:3. ^1^H NMR (400 MHz, CDCl_3_): δ 7.35–6.96 (m, 20H, Ar), 5.75 (d, 1H, *J* = 7.4 Hz, 2-NH), 5.24 (d, 1H, *J* = 12.0 Hz, OCH_2_), 5.14 (d, 1H, *J* = 12.0 Hz, OCH_2_), 5.05 (s, 2H, OCH_2_), 4.17 (m, 1H, 2′-H), 3.43 (d, 1H, *J* = 14.5 Hz, 4-CH_2_), 3.23 (dd, 1H, *J* = 14.5, 8.0, 1′-H), 3.11 (q, 1H, *J* = 7.6 Hz, 3-H), 3.05 (d, 1H, *J* = 14.5 Hz, 4-CH_2_), 3.00 (dd, 1H, *J* = 14.5, 4.0 Hz, 1′-H), 2.86 (dd, 1H, *J* = 14.0, 8.0 Hz, 3′-H), 2.72 (dd, 1H, *J* = 14.0, 7.0 Hz, 3′-H), 1.08 (d, 3H, *J* = 7.5 Hz, 3-CH_3_).^13^C NMR (75 MHz, CDCl_3_): δ 171.0 (COO), 170.6 (C2), 156.0 (OCON), 137.8, 137.0, 134.9, 134.8, 129.7, 129.2, 128.9, 128.85, 128.8, 128.7, 128.5, 128.45, 127.9, 127.8, 127.5, 126.5 (Ar), 68.7 (C4), 67. 7, 66.3 (OCH_2_), 53.8 (C3), 51. 9 (C2′), 46.1 (C1′), 40.7 (4-CH_2_). 39.1 (C3′), 10.6 (3-CH_3_). MS (ES)^+^: 577.3 [M + H]^+^. Exact Mass calculated for C_36_H_36_N_2_O_5_: 576.26242; found: 576.26457.

### 4*S*-Benzyl-4-benzyloxycarbonyl-3*S*-methyl-1-[(2′R-benzyloxycarbonylamino-3′-phenyl)prop-1′-yl]-2-oxoazetidine (29a)

Syrup. Yield: 39% (from 27, B: BTPP). Eluent: EtOAc:Hexane (1:3). HPLC: t_R_ = 16.36 min (gradient of 30% to 95% of A, in 20 min).[α]_D_ = -72.67 (c 1, CHCl_3_). Isomer ratio M(3*S*,4*S,2′R*):m(3*R*,4*S,2′R*) = 97:3. ^1^H NMR (400 MHz, CDCl_3_): δ 7.37–6.99 (m, 20H, Ar), 5.83 (d, 1H, *J* = 8.1 Hz, 2-NH), 5.27 (d, 1H, *J* = 12.0 Hz, OCH_2_), 5.17 (d, 1H, *J* = 12.0 Hz, OCH_2_), 5.05 (s, 2H, OCH_2_), 4.07 (m, 1H, 2′-H), 3.56 (d, 1H, *J* = 14.5 Hz, 4-CH_2_), 3.43 (q, 1H, *J* = 7.6 Hz, 3-H), 3.03 (d, 1H, *J* = 14.5 Hz, 4-CH_2_), 2.99 (m, 2H, 1′-H), 2.71 (dd, 1H, *J* = 13.5, 7.4 Hz, 3′-H), 2.64 (dd, 1H, *J* = 13.4, 6.6 Hz, 3′-H), 1.08 (d, 3H, *J* = 7.5 Hz, 3-CH_3_). ^13^C NMR (100 MHz, CDCl_3_): δ 171.3 (COO), 170.3 (C2), 156.3 (OCON), 137.5, 135.1, 134.8, 129.7, 129.5, 129.2, 129.0, 128.9, 128.8, 128.7, 128.5, 128.4, 127.9, 127.5, 126.5 (Ar), 69.0 (C4), 67.8, 66.3 (OCH_2_), 54.1 (C3), 51.6 (C2′), 47.0 (C1′), 41.2 (4-CH_2_). 39.3 (C3′), 10.6 (3-CH_3_). MS (ES)^+^: 577.25 [M + H]^+^. Exact Mass calculated for C_36_H_36_N_2_O_5_: 576.26242; found: 576.26248.

### Functional assays by calcium microfluorimetry

Human embryonic kidney cell line (HEK) stably transfected with rTRPM8 or h-TRPV1 were used as previously described^37^. Briefly, cells were seeded in 96-well plates at a cell density of 30,000 cells 2 days before treatment. Buffer used was HBBS (in mM): 138 NaCl, 5.33 KCl, 1.26 CaCl_2_, 0.5 MgCl-6H_2_O, 0.4 MgSO_4_-7H_2_O, 4 NaHCO3 0.44 KH_2_PO_4_, 0.3 Na_2_HPO_4_, pH 7.4. The day of treatment the medium was replaced with 100 µL of the dye loading solution Fluo-4 NW supplemented with probenecid 2.5 mM. After incubation, plates were transferred to a fluorescence plate reader (Polastar BMG). In the experimental protocol, the baseline fluorescence was recorded (Em 485 nm/Ex 520 nm) for 3 cycles. After, vehicle, compound at different concentrations and the corresponding antagonist, 10 µM AMTB for TRPM8 or 10 µM Ruthenium Red for TRPV1 were added to the well. Fluorescence intensity was recorded during 7 cycles more. Finally, the agonist 100 µM menthol for TRPM8 or 1 µM capsaicin for TRPV1 was added and fluorescence intensity was recorded during 10 cycles more.

#### Data analysis

The degree of inhibition of TRPM8 channel gating was calculated by comparison to calcium increase signal elicited by test substances and menthol at 100 µM. Decrease of menthol signal was expressed as percentage of inhibition (%). All data are expressed as mean ± standard deviation (SD). Each condition was assessed by triplicate (n = 3) in 3 independent experiments (N = 3). Z-factor was calculated in each assay using the following equation: (3*(SDmax + SDmin))/(Mean max-Mean min). In all the experiments Z-factor was ≥ 0.5. To calculate IC_50_, normalized responses (%) versus log [µM] were adjusted to a non-linear fit with variable slope, a four-parameter dose–response curve following curve Y = 100/(1 + 10^((Log IC_50_-X)*HillSlope)) where X = % normalized response and Y = log [µM].

### Functional assays by Patch-clamp electrophysiology

Electrophysiological recording was carried out 1–3 days after cells seeded. Membrane currents and voltages were recorded by patch clamp using the whole-cell configuration. For whole-cell recordings of HEK-rTRPM8 cells^37^, pipette solution contained (in mM) 150 NaCl, 5 EGTA, 3 MgCl_2_ and 10 HEPES, adjusted to pH 7.2 with NaOH, and bath solution contained (in mM) 150 NaCl, 6 CsCl, 1.5 CaCl_2_, 1 MgCl_2_, 10 d-glucose and 10 HEPES, adjusted to pH 7.4 with NaOH. Data were sampled at 10 kHz (EPC10 amplifier with PatchMaster 2.53 software, HEKA Electronics, Lambrecht, Germany) and low-pass filtered at 3 kHz for analysis (PatchMaster 2.53 and GraphPad Prism 5, Graphpad Software, USA). The series resistance was < 10 MΩ and to minimize voltage errors was compensated to 60–80%. All measurements were performed at 24–25 °C. The TRPM8 response was quantified as the ratio P2/P1, being P2 the second menthol pulse in the presence of the vehicle or antagonist tested, and P1 refers to the first menthol pulse. This paradigm considers the extend of receptor desensitization as the P2/P1 obtained with the vehicle. Thus, the estimated IC50 values are corrected from receptor desensitization.

### Docking Studies

The molecular model for rat TRPM8 was obtained using the structure of the TRPM8 from *Ficedula albicollis* determined by cryo-electron microscopy at 4.01 resolution^24^. First, the missing loops in the structure from *Ficedula albicolis* was completed by using Yasara^44,61^. Second, the rat TRPM8 sequence (Uniprot Q8R455) was modeled on completed *Ficedula a.* structure, although the N-term (44–719) and C-term (1,031–1,104) were removed for docking. The homology modelling was performed with the standard homology modelling protocol implemented in Yasara (version 19.9.17)^44,45^. Sequence alignment between rat and *Ficedula a.* TRPM8 was performed with ClustalO^62^, from the European Bioinformatic Institute (EBI, https://www.ebi.ac.uk). The visualization and edition of the molecules were also done with Yasara (https://www.yasara.org). Figures were drawn with open source Pymol (The PyMOL Molecular Graphics System, Version 1.8 Schrödinger, LLC) at https://www.pymol.org.

The global docking procedure was accomplished with AutoDock^46^, implemented in Yasara, in which a total of 800 flexible docking runs were set and clustered around the putative binding sites. The program then performed a simulated annealing optimization of the complexes, which moved the structure to a nearby stable energy minimum, by using the implemented Assisted Model Building with Energy Refinement (AMBER03) force field^63^. The Yasara pH command was set to 7.0, to ensure that molecules preserved their pH dependency of bond orders and protonation patterns. The best binding energy complex in each cluster was stored, analyzed, and used to select the best orientation of the interacting partners.

### Antitumor activity

#### Cell lines

Human tumor cell lines used in this study were purchased from the ATCC. A-549 (CCL-185), lung carcinoma; HT-29 (HTB-38), colorectal adenocarcinoma; MDA-MB-231 (HTB-26), breast adenocarcinoma; PSN1, pancreas adenocarcinoma.

#### Cell culture

All cell lines were maintained in DMEM (Dulbecco’s Modified Eagle's Medium) culture medium supplemented with 10% FBS (Fetal Bovine Serum), 100 Units/mL penicillin/streptomycin at 37 °C, 5% CO_2_ and 95% humidity.

#### Cytotoxicity Assay

Triplicate cultures were incubated for 72 h in the presence or absence of test compounds in dose–response curves (10 concentrations, typically ranging from 10 to 0.0026 µg/mL). A colorimetric assay using sulforhodamine B (SRB) was adapted for quantitative measurement of cell growth and cytotoxicity^27^. A more detailed information on this assay is provides in the supplementary information. GI_50_ is the compound concentration that produces 50% inhibition on cell growth as compared to control cells.

### In vivo anti-allodynic effects

Male C57-mice (≈30 g) (Harlam, Holland) were used for the study. All experiments were approved by the Institutional Animal and Ethical Committee of the Universidad Miguel Hernandez where experiments were conducted and they were in accordance with the guidelines of the Economic European Community and the Committee for Research and Ethical Issues of the International Association for the Study of Pain. All parts of the study concerning animal care were performed under the control of veterinarians.

As previously described^34^, oxaliplatin (Tocris) was dissolved in water with gentle warming and was subcutaneously (s.c.) injected on days 1, 3 and 5 at a 6 mg/kg dose. The day 7 after administration, experiments were performed. Together with Oxaliplatin injection, saline and a 5% Mannitol solution were intraperitoneally injected to prevent kidney damage and dehydration. The compound 24a stock was prepared in DMSO (Sigma-Aldrich) and diluted in saline for injections. Compound at different doses (1 to 3 μg) was injected into the plantar surface (25 μL) of the right hind paw of mice. Cold chemical thermal sensitivity was assessed using acetone drop method. Mice were placed in a metal mesh cage and allowed to habituate for approximately 30 min in order to acclimatize them. Freshly dispensed acetone drop (10μL) was applied gently on to the mid plantar surface of the hind paw. Cold chemical sensitive reaction with respect to paw licking was recorded as a positive response (nociceptive pain response). The responses were measured for 20-s with a digital stopwatch. For each measurement, the paw was sampled twice and the mean was calculated. The interval between each application of acetone was approximately 5 min.

## Supplementary information

Supplementary information 1

Supplementary information 2

## Data Availability

The datasets generated during and/or analyzed during the current study are available from the corresponding author on reasonable request.
