# Supplementary material for: Highly functionalized β-lactams and 2-ketopiperazines as TRPM8 antagonists with antiallodynic activity
Source: Sci Rep. 2020 Aug 25;10:14154. doi: 10.1038/s41598-020-70691-x (PMC7447632; doi:10.1038/s41598-020-70691-x)

Highly functionalized β-lactams and 2-ketopiperazines as TRPM8 antagonists with antitumor and antiallodynic activity

M. Ángeles Bonache, Cristina Martín-Escura, Roberto de la Torre Martínez, Alicia Medina, Sara González-Rodríguez, Francesc Solloso, Carmen Cuevas, Ana María Roa, Gregorio Fernández-Ballester, Antonio Ferrer-Montiel, Asia Fernández-Carvajal, Rosario González-Muñiz

SUPPLEMENTARY INFORMATION-SPECTRA

^{1}H-NMR (400 MHz, $CDCl_3$) and ^{13}C-NMR (75 MHz, $CDCl_3$). Compound 6

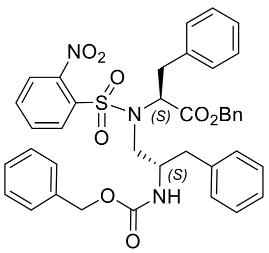

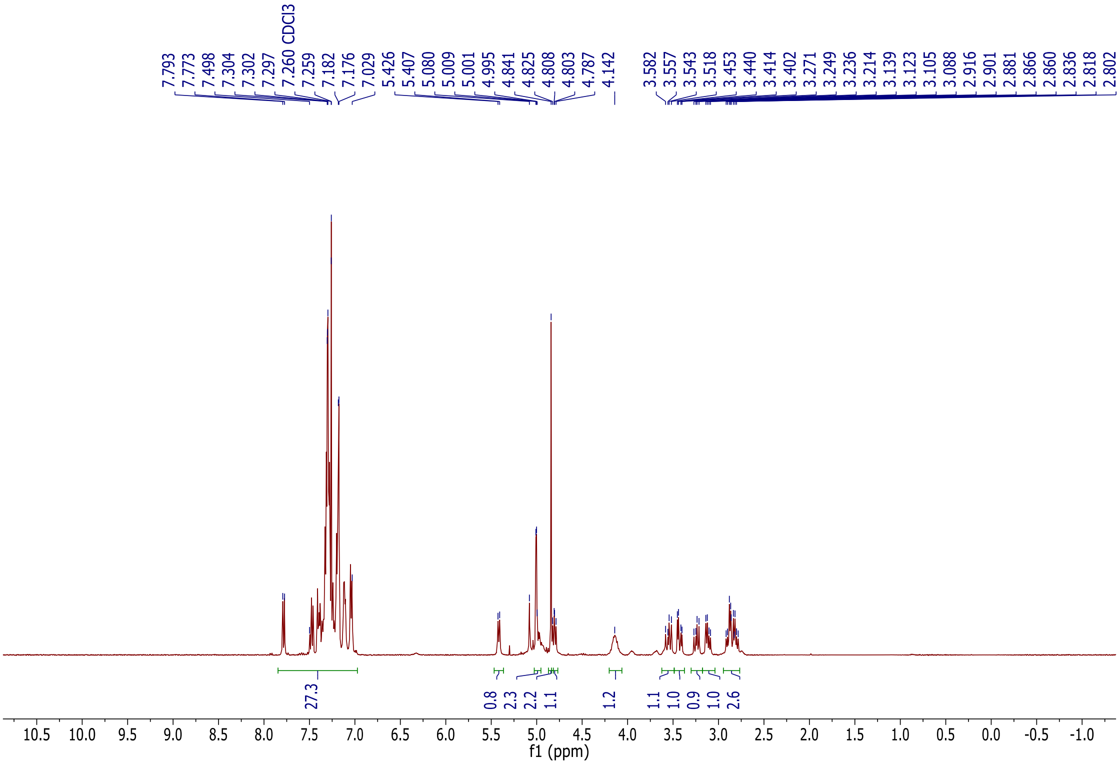

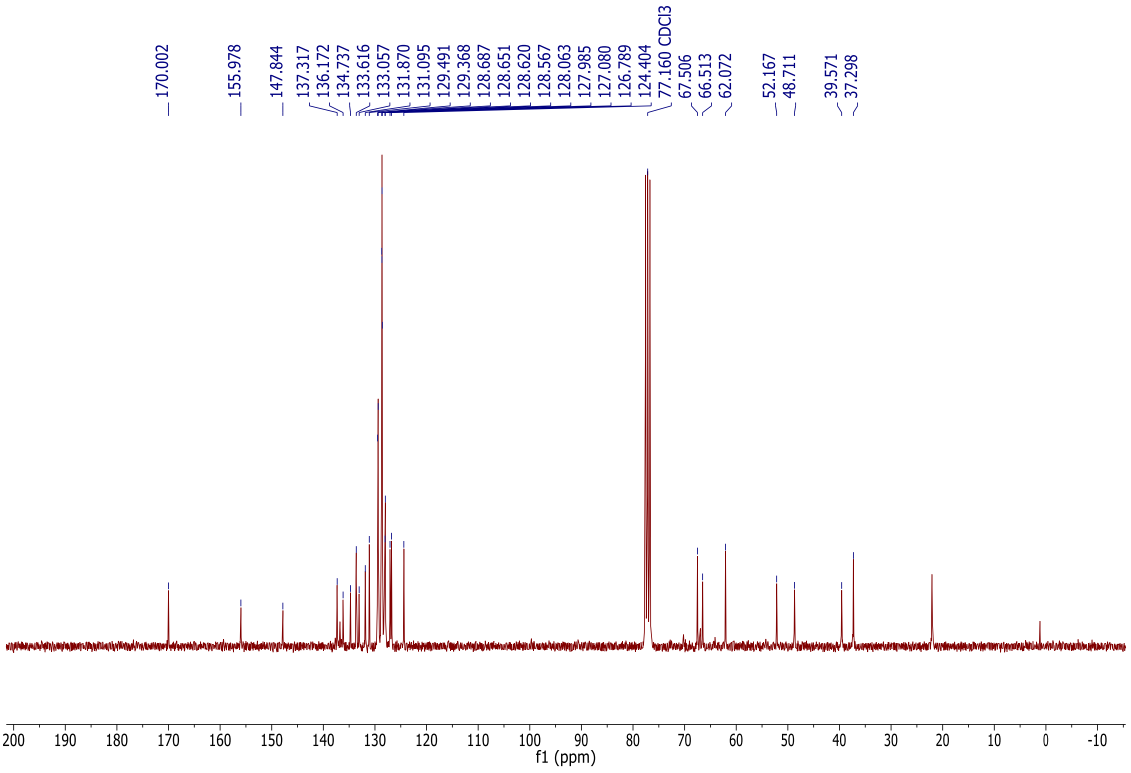

^{1}H-NMR (400 MHz, $CDCl_3$) and ^{13}C-NMR (75 MHz, $CDCl_3$). Compound 7

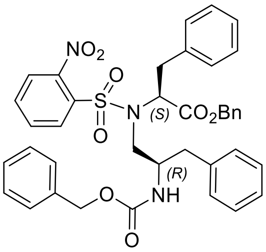

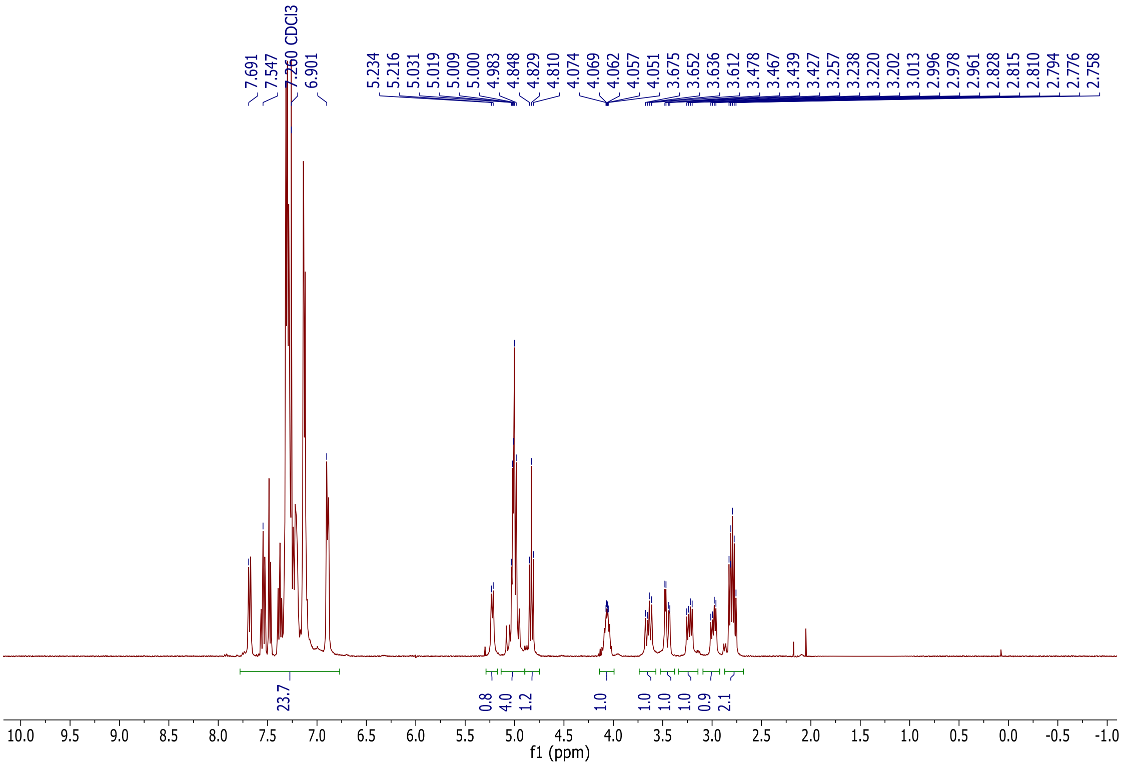

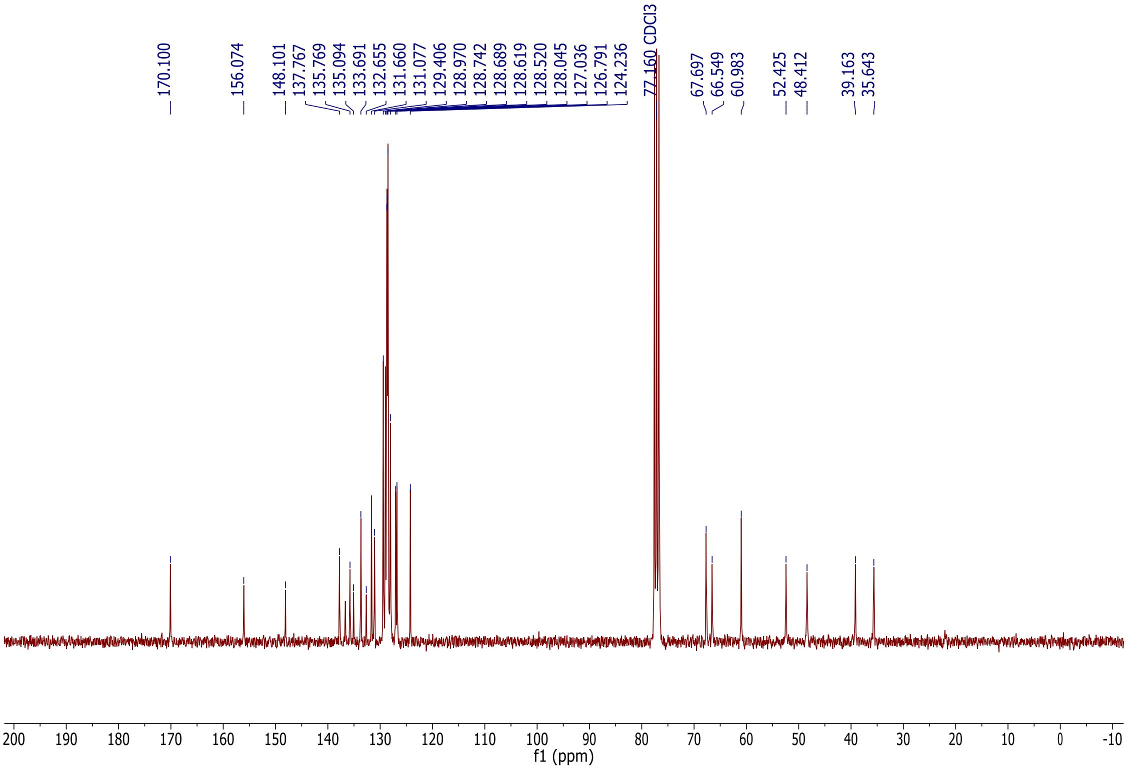

^{1}H-NMR (400 MHz, $CDCl_3$) and ^{13}C-NMR (75 MHz, $CDCl_3$). Compound 8

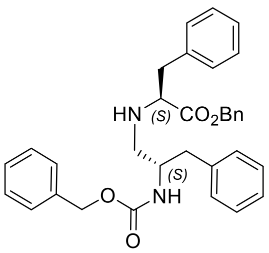

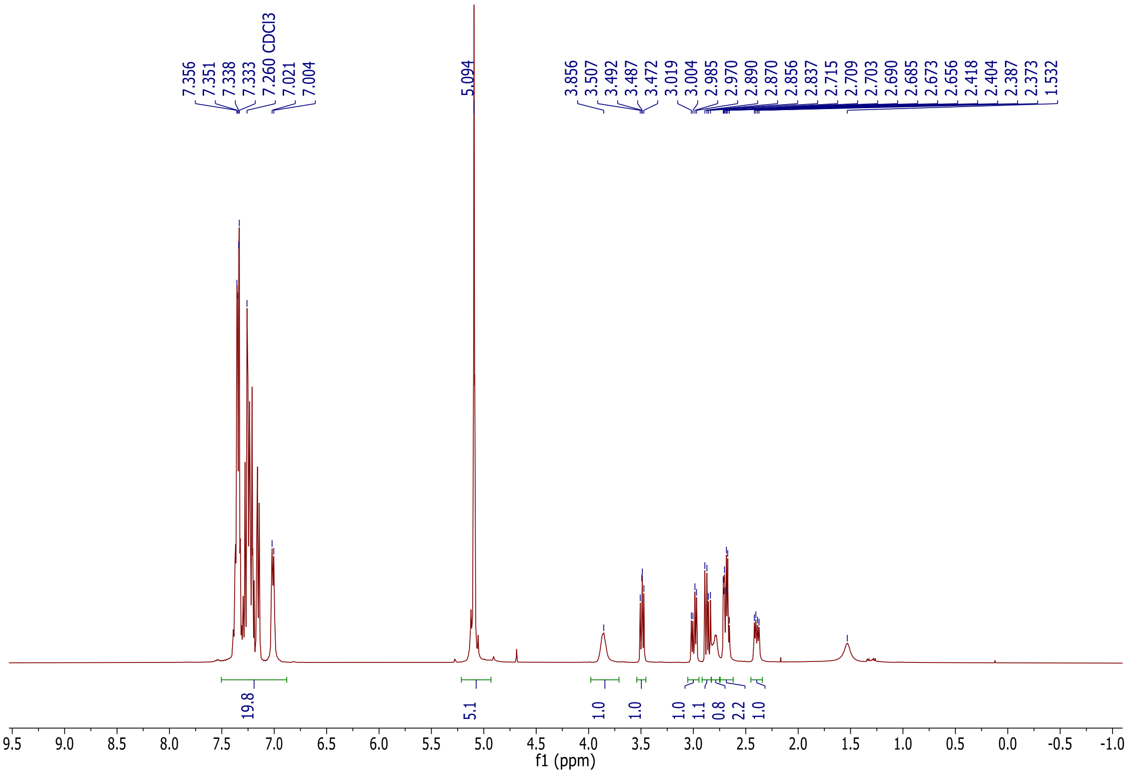

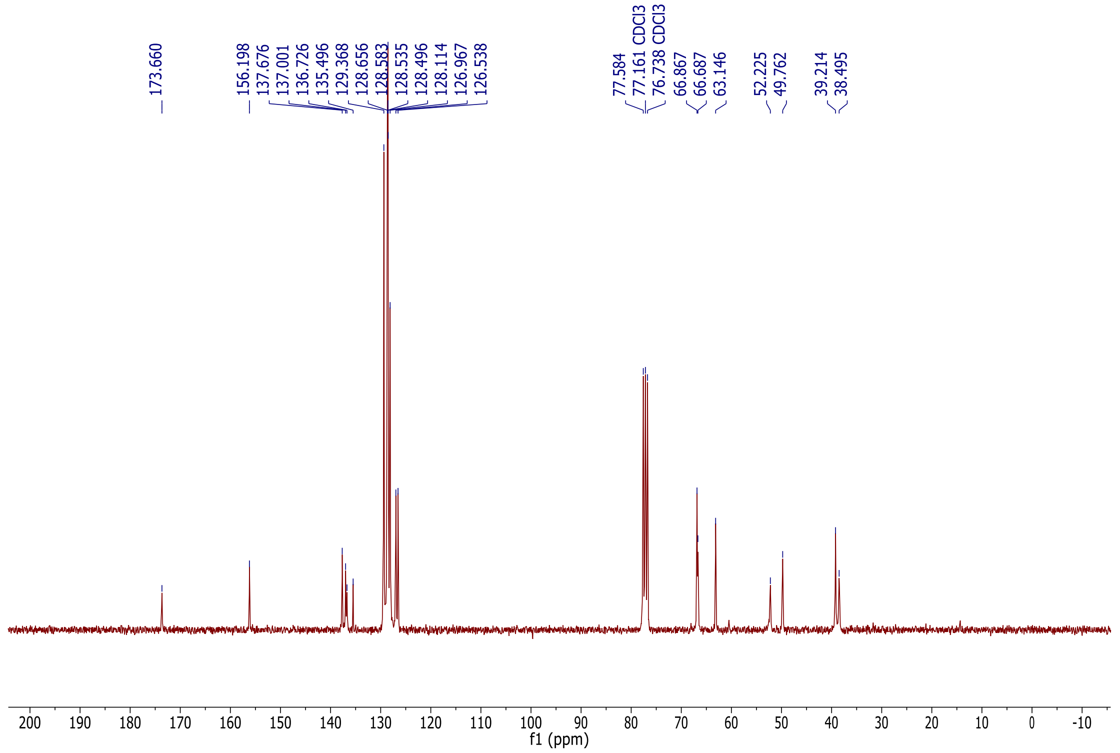

^{1}H-NMR (400 MHz, $CDCl_3$) and ^{13}C-NMR (75 MHz, $CDCl_3$). Compound 9

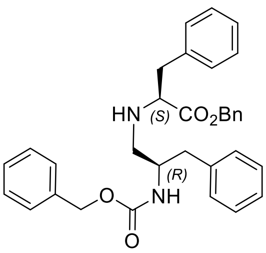

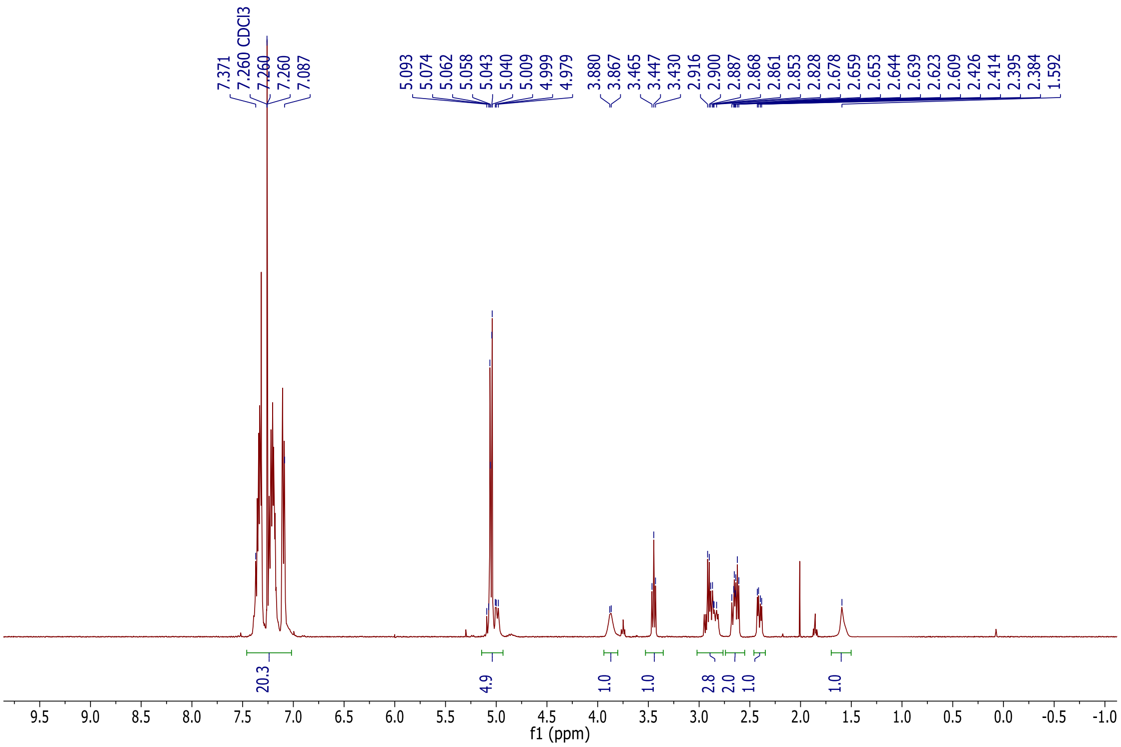

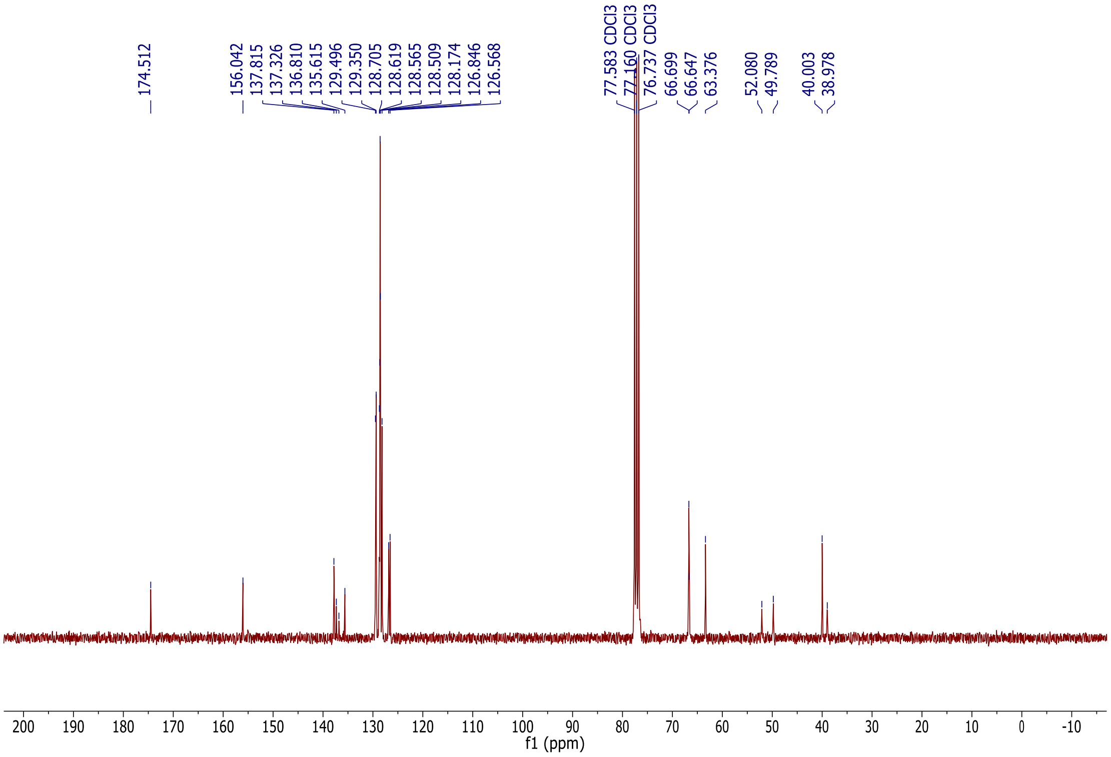

^{1}H-NMR (400 MHz, DMSO-d_6) and ^{13}C-NMR (75 MHz, DMSO-d_6). Compound 10

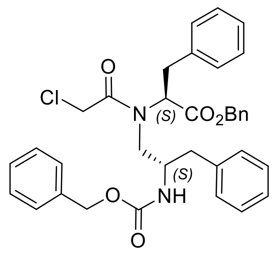

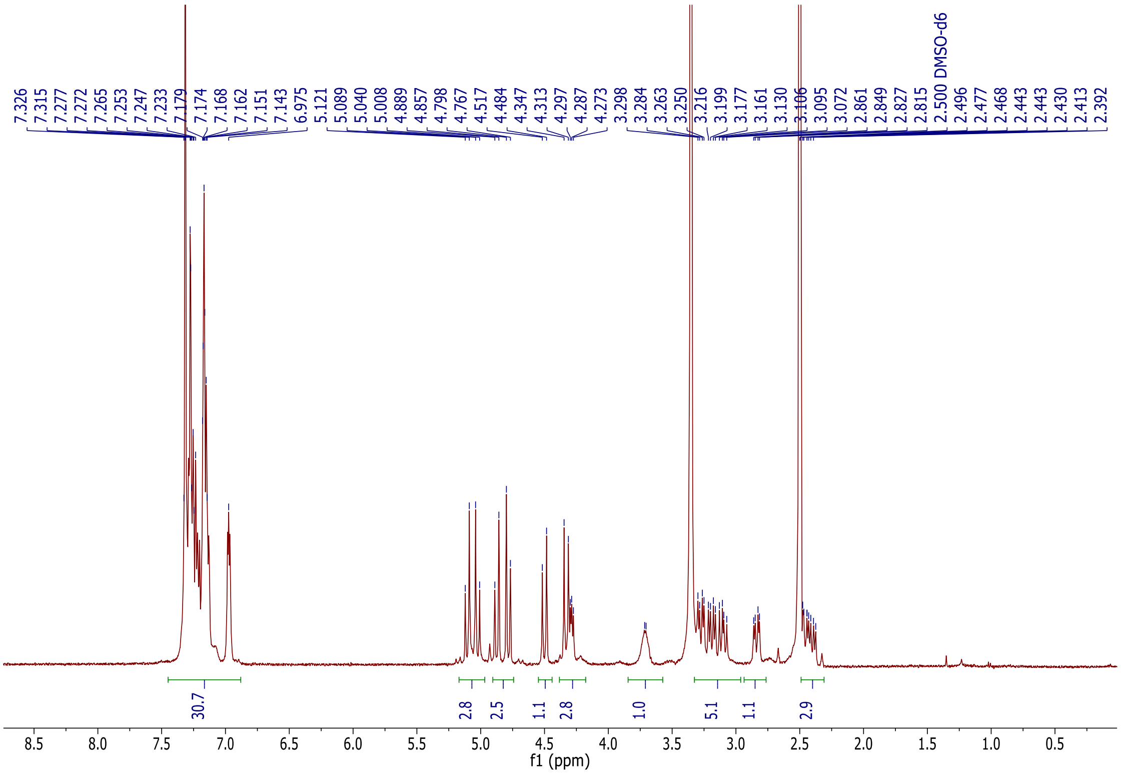

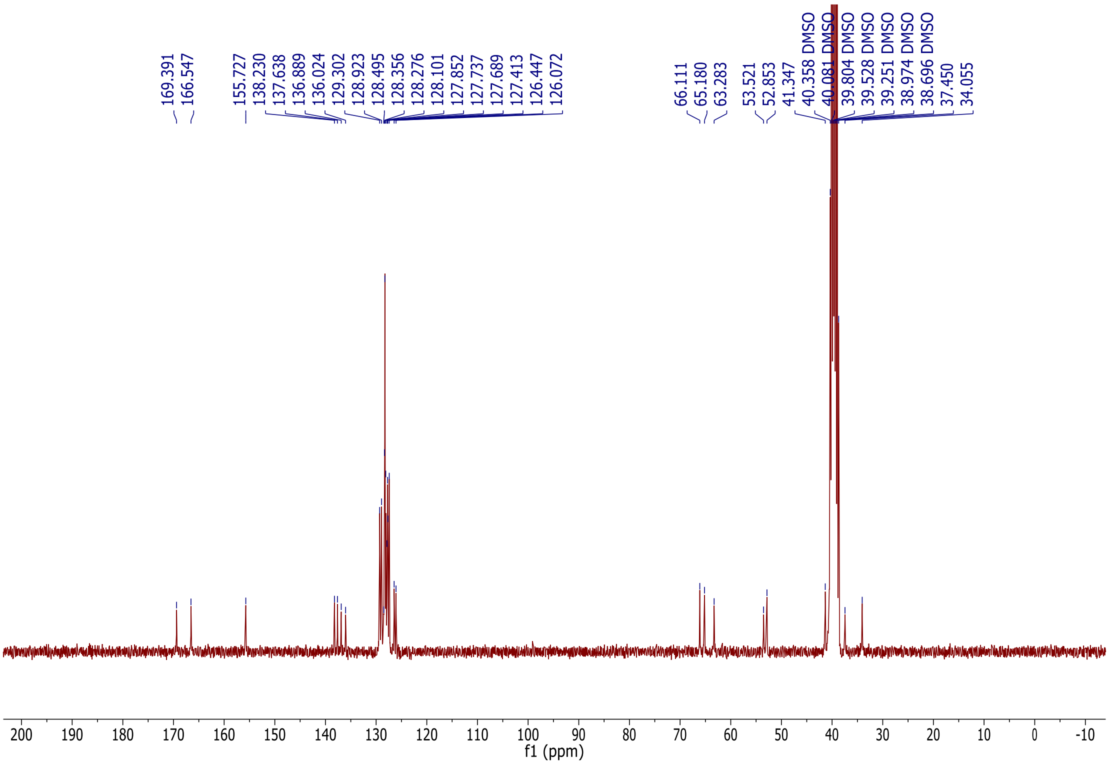

^{1}H-NMR (400 MHz, DMSO-d_6) and ^{13}C-NMR (75 MHz, DMSO-d_6). Compound 11

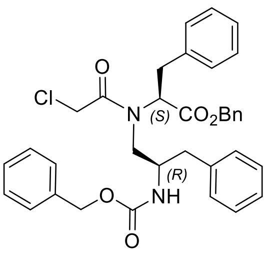

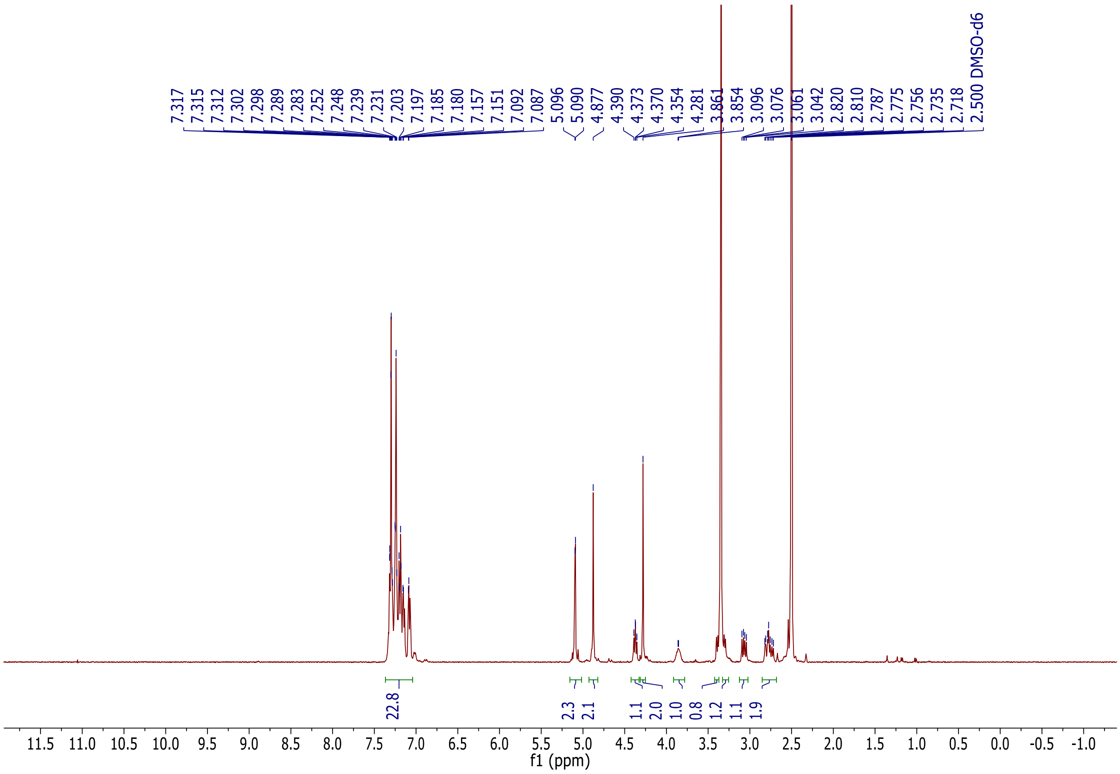

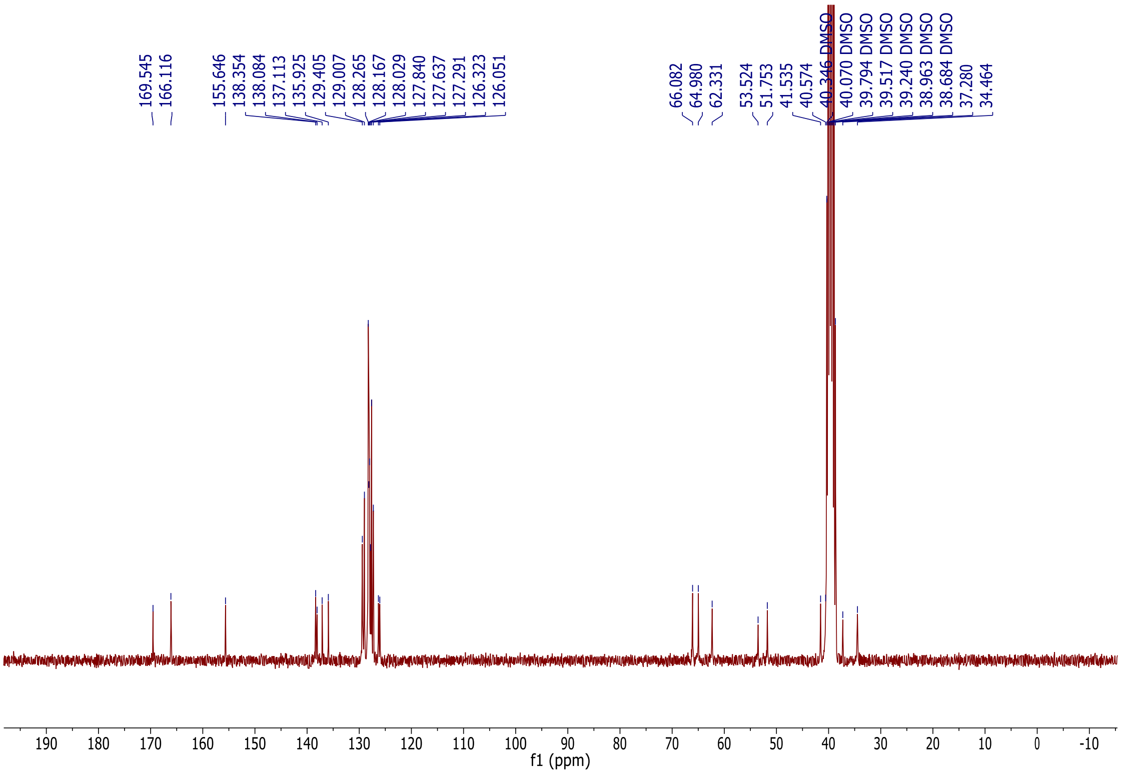

^{1}H-NMR (400 MHz, $CDCl_3$) and ^{13}C-NMR (75 MHz, $CDCl_3$). Compound 12ab

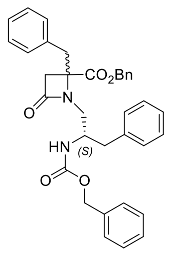

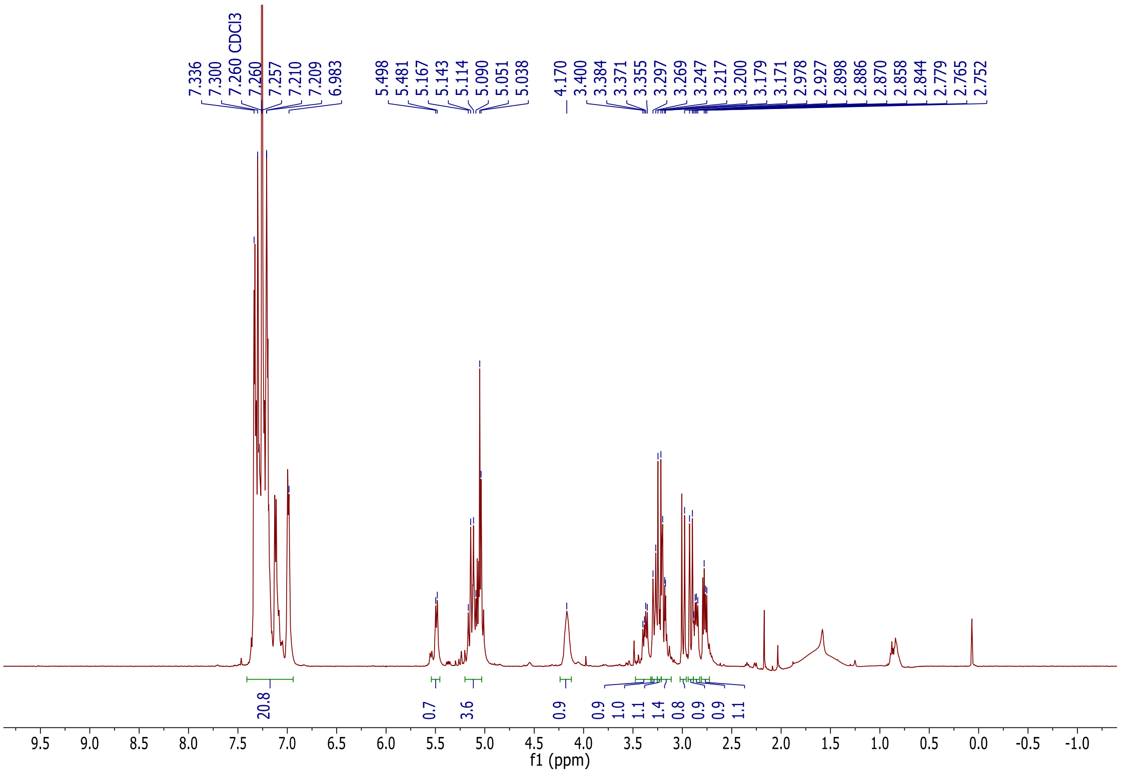

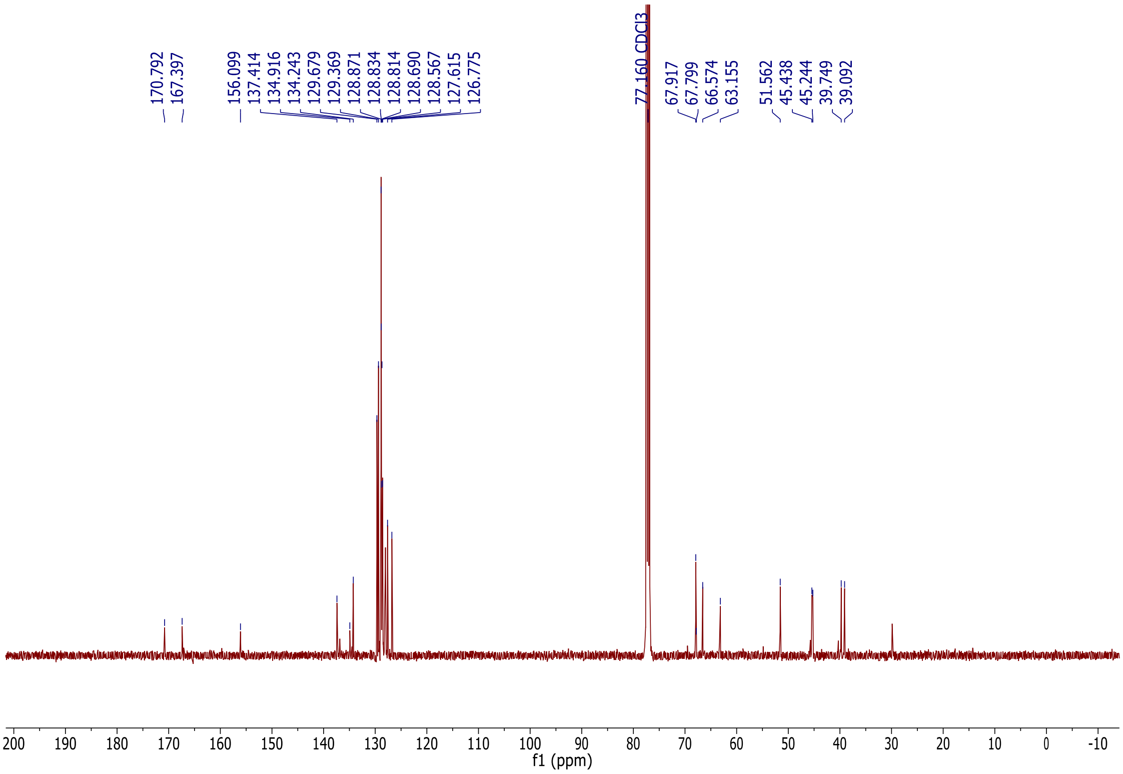

^{1}H-NMR (400 MHz, DMSO-d_6) and ^{13}C-NMR (100 MHz, DMSO-$d_{6,}$) 90 °C. Compound 13ab

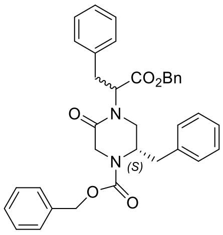

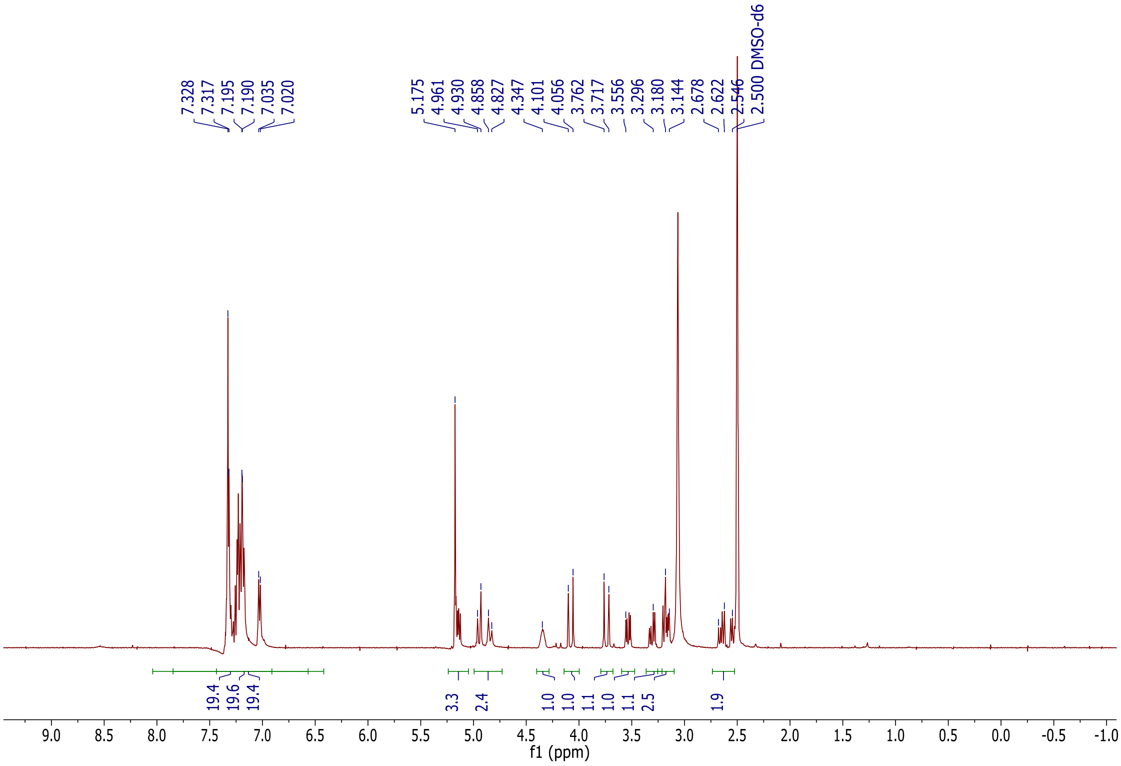

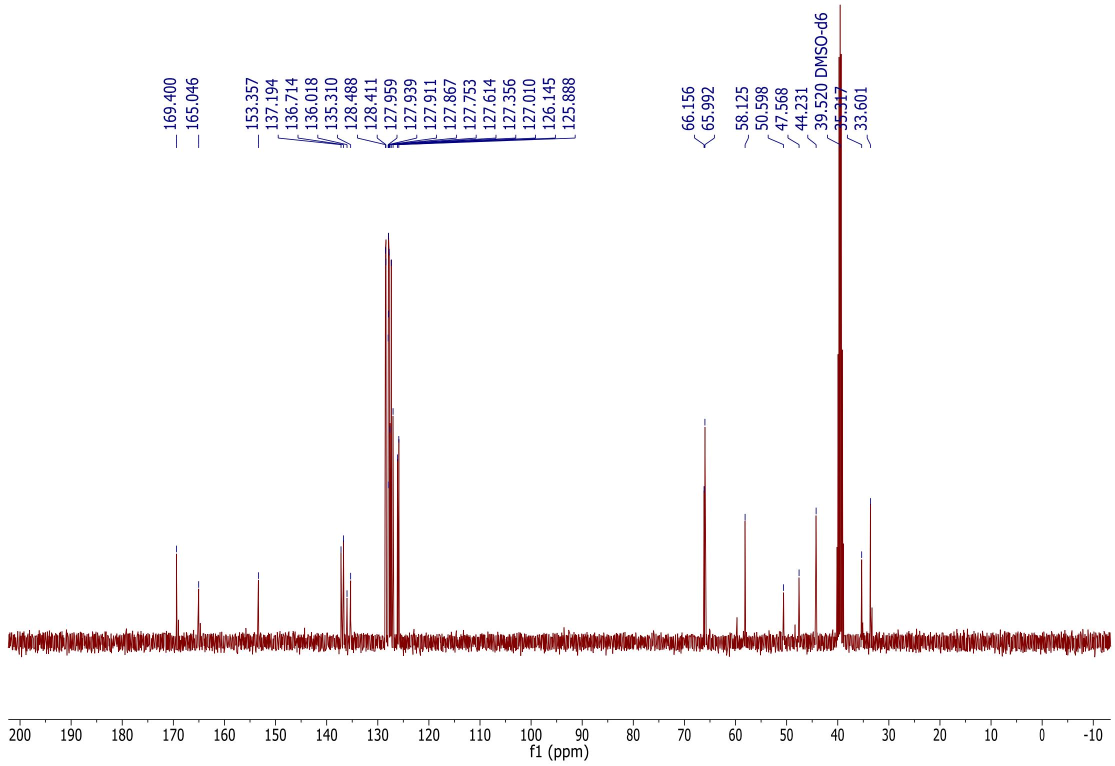

^{1}H-NMR (400 MHz, $CDCl_3$) and ^{13}C-NMR (75 MHz, $CDCl_3$). Compound 14ab

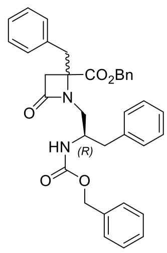

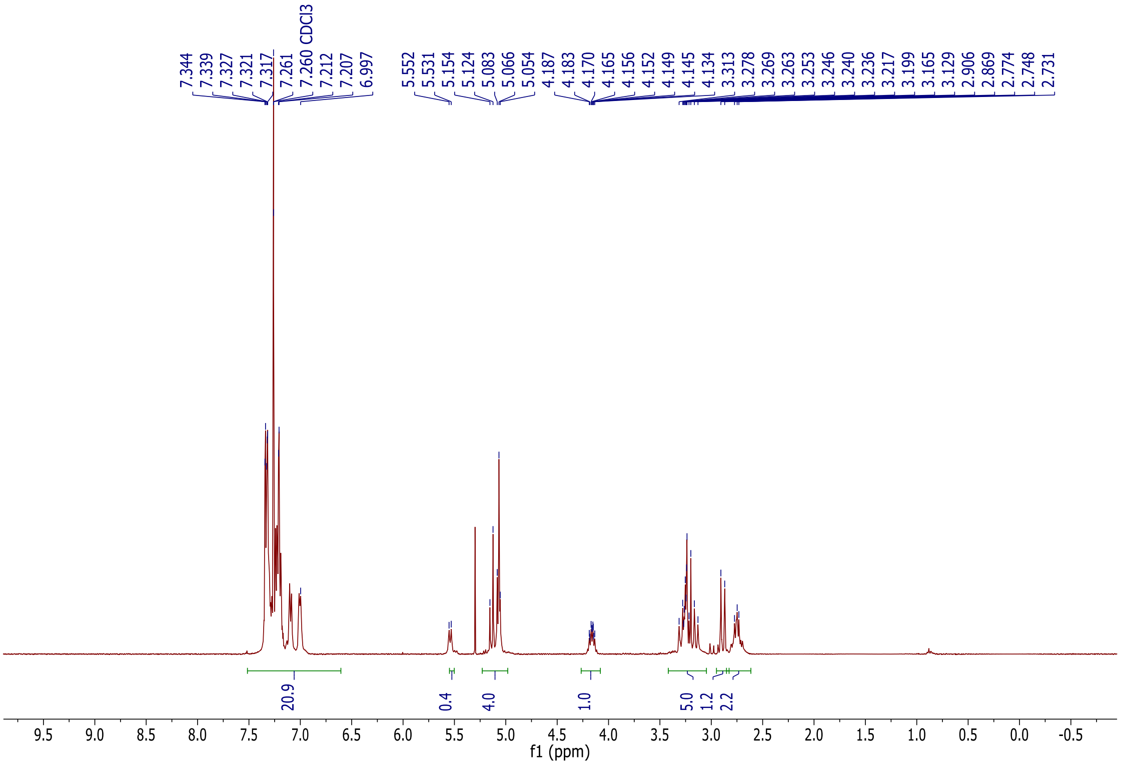

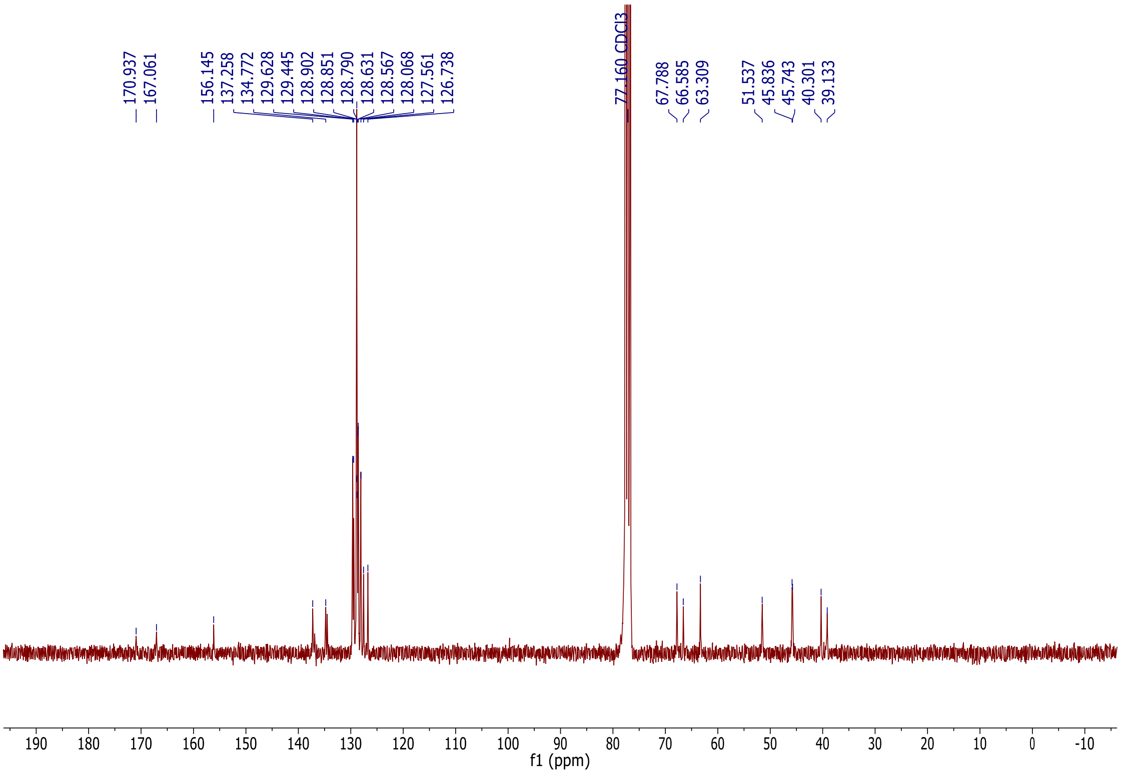

^{1}H-NMR (400 MHz, DMSO-d_6) and ^{13}C-NMR (100 MHz, DMSO-$d_{6,}$) 90 °C. Compound 15ab

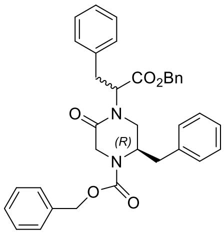

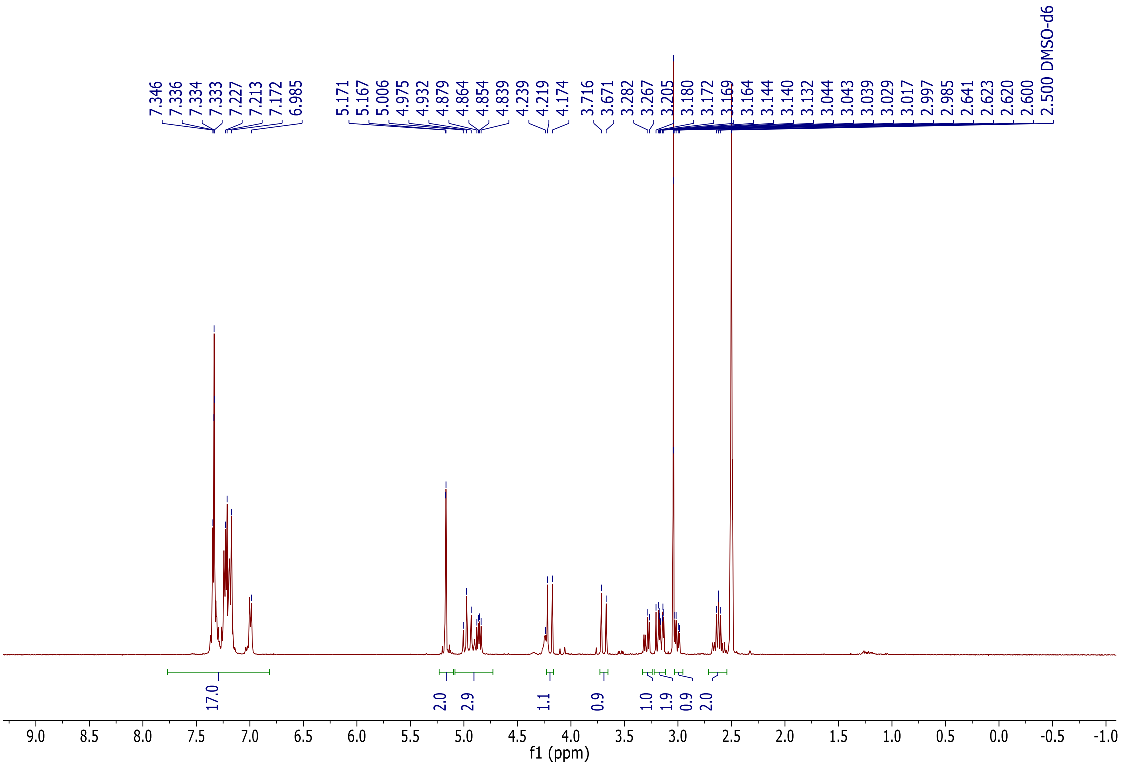

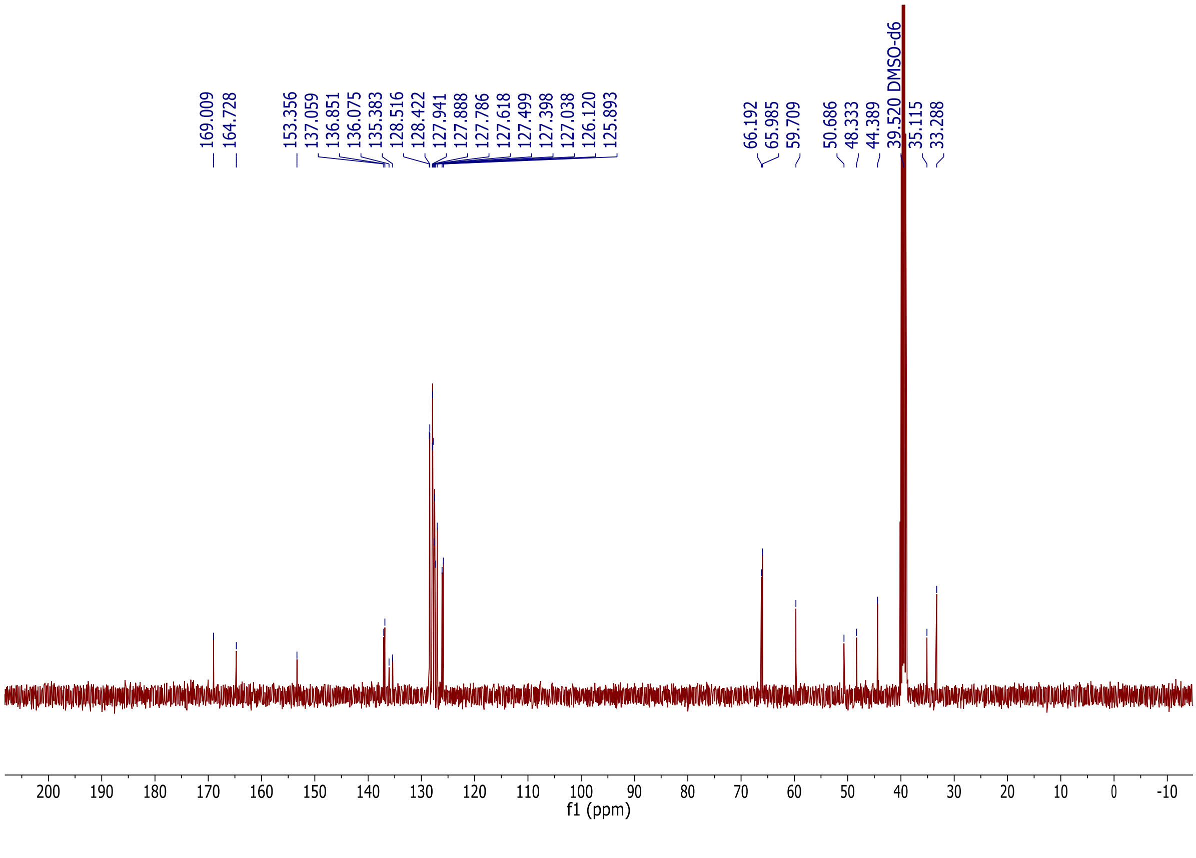

^{1}H-NMR (400 MHz, $CDCl_3$) and ^{13}C-NMR (75 MHz, $CDCl_3$). Compound 17

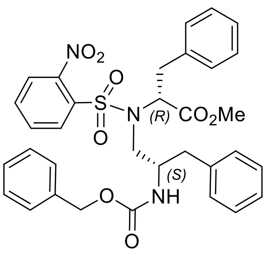

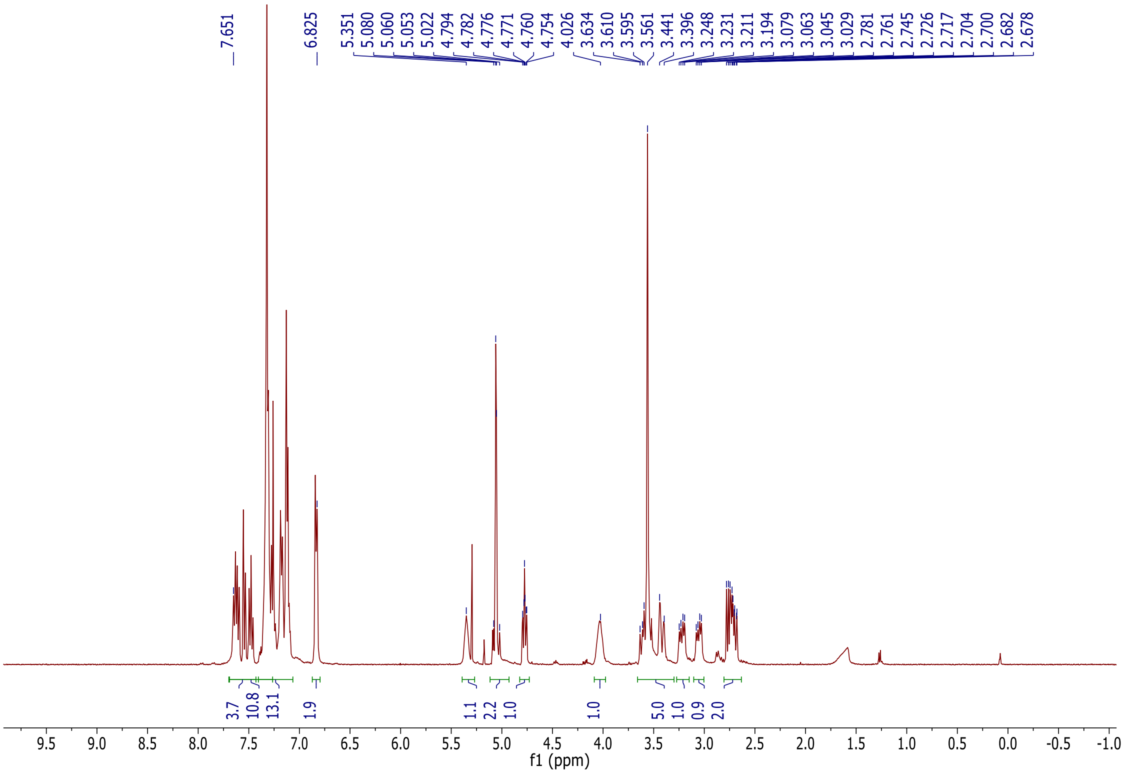

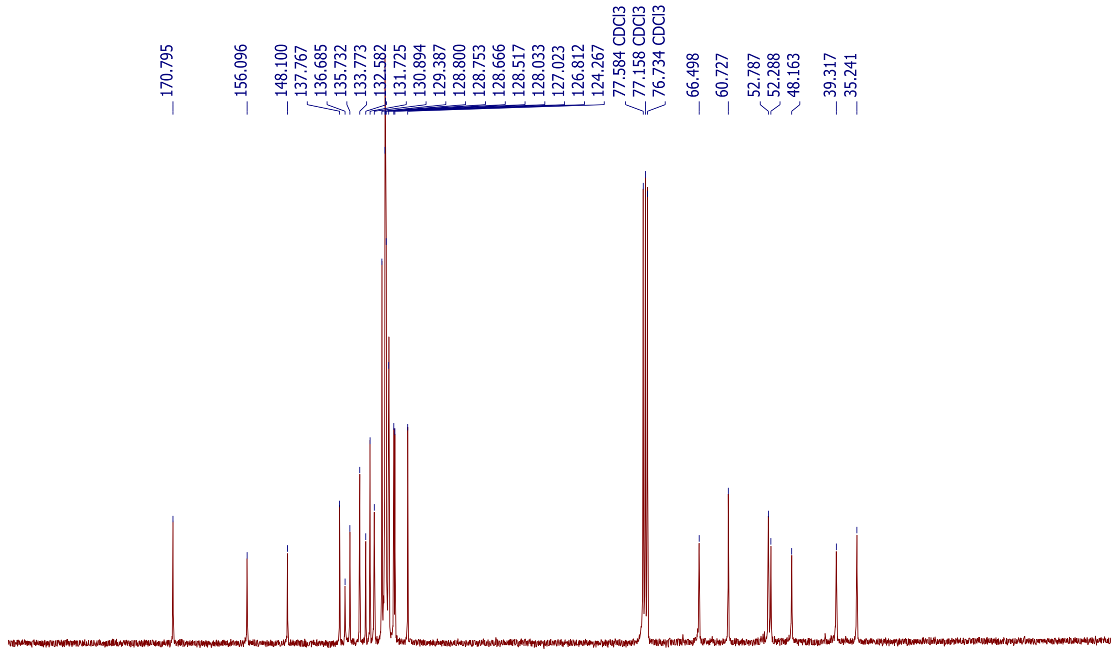

^{1}H-NMR (400 MHz, $CDCl_3$) and ^{13}C-NMR (75 MHz, $CDCl_3$). Compound 18

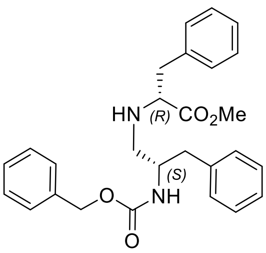

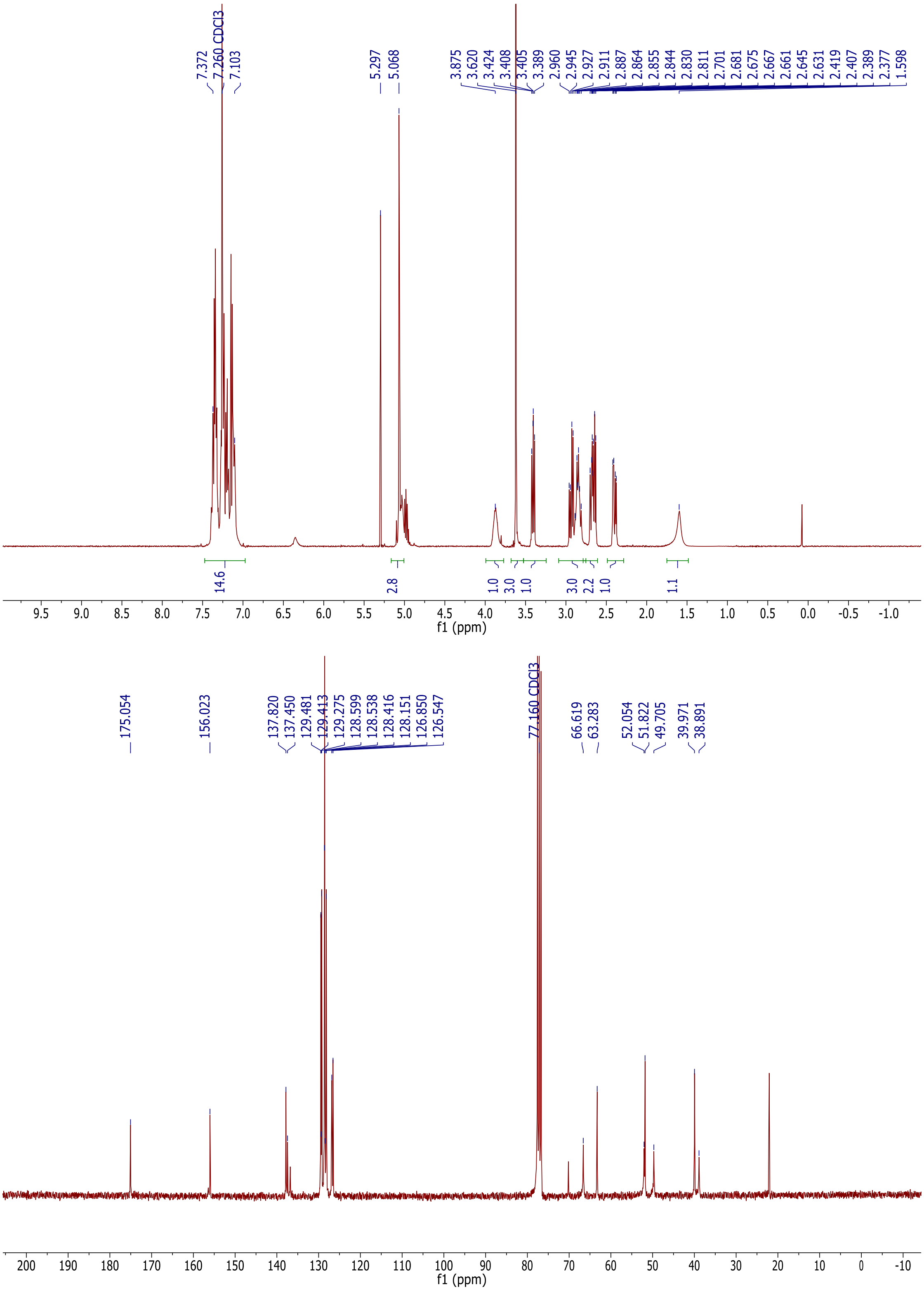

^{1}H-NMR (400 MHz, DMSO-d_6) and ^{13}C-NMR (75 MHz, DMSO-d_6). Compound 19

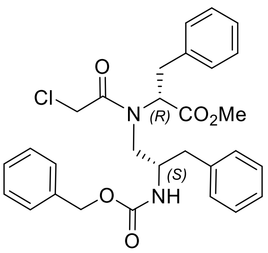

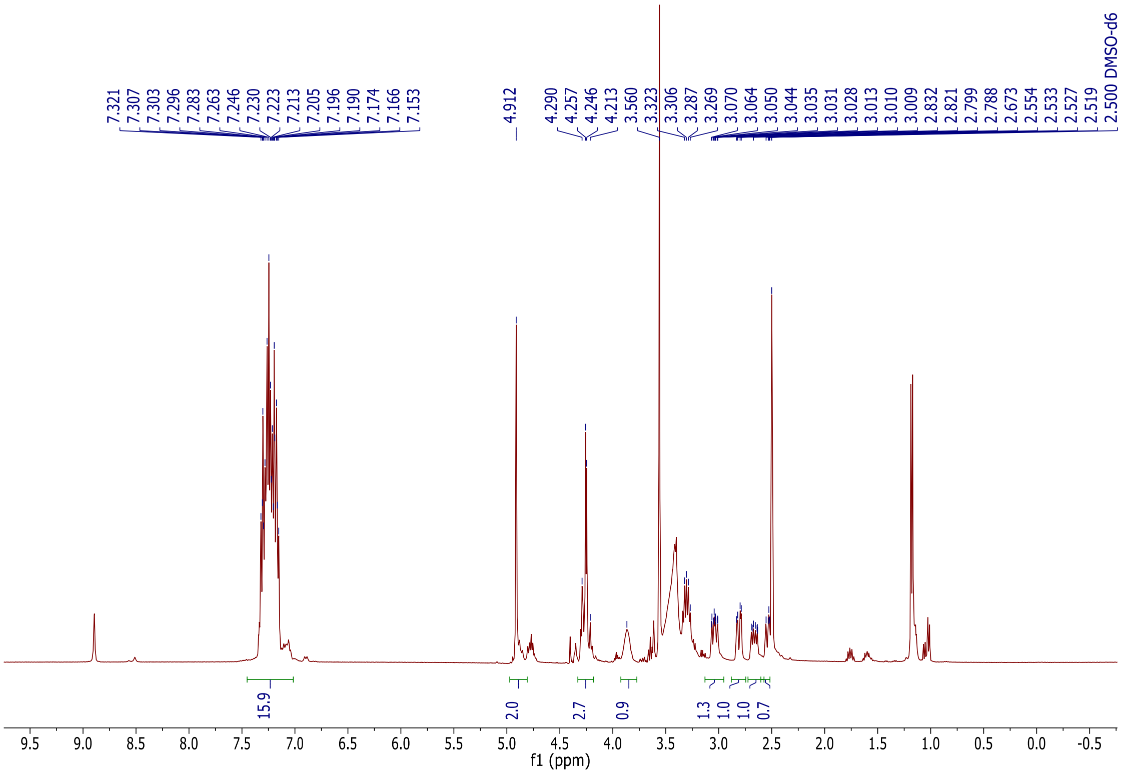

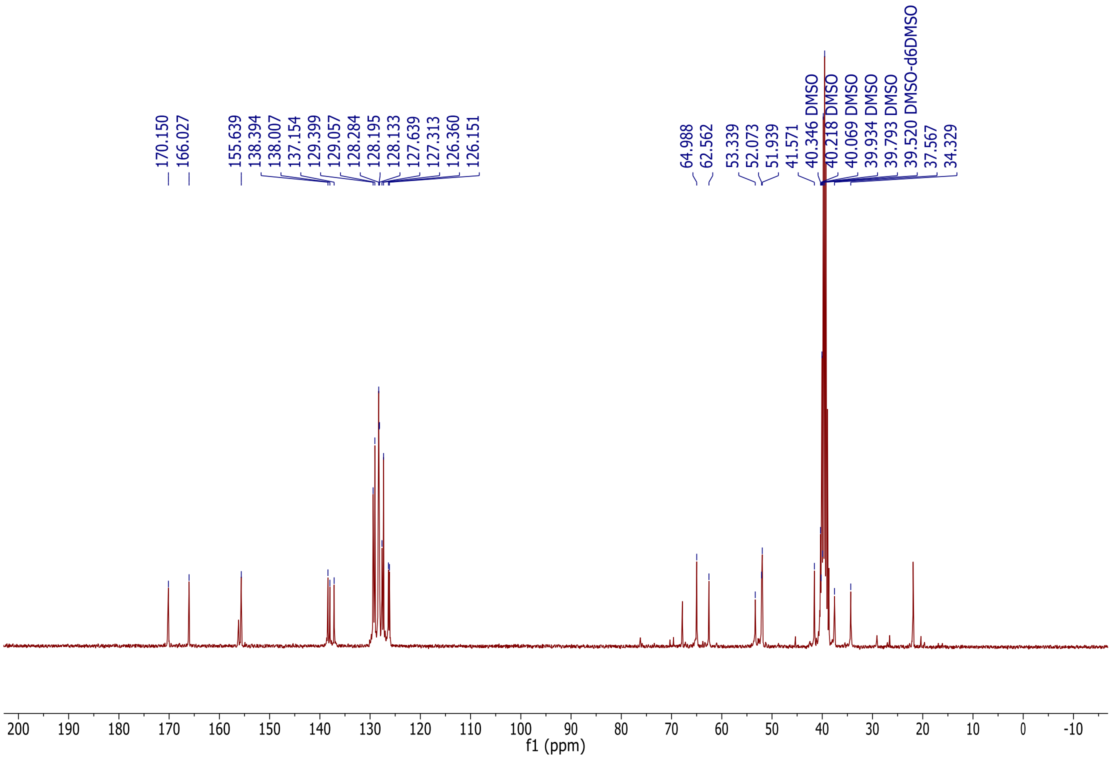

^{1}H-NMR (400 MHz, DMSO-*d*$_6$) and ^{13}C-NMR (100 MHz, DMSO-*d*$_{6,}$) 90 °C. Compound 20ab

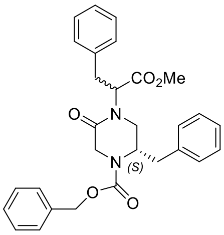

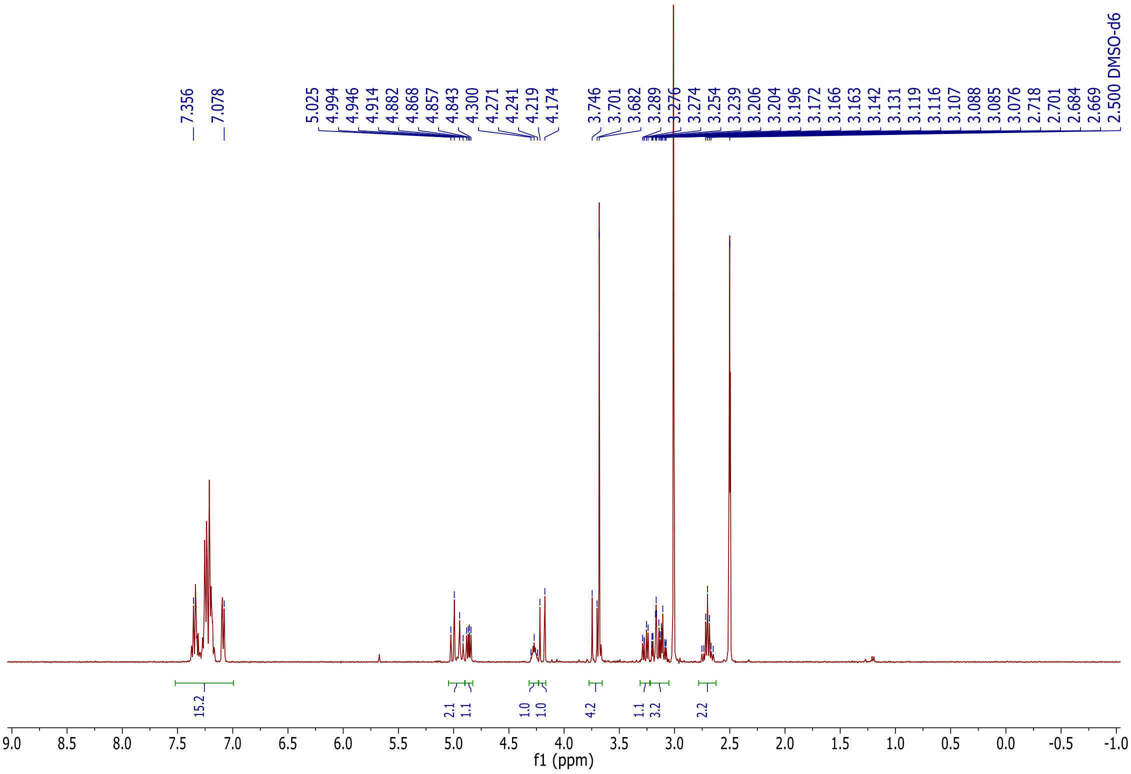

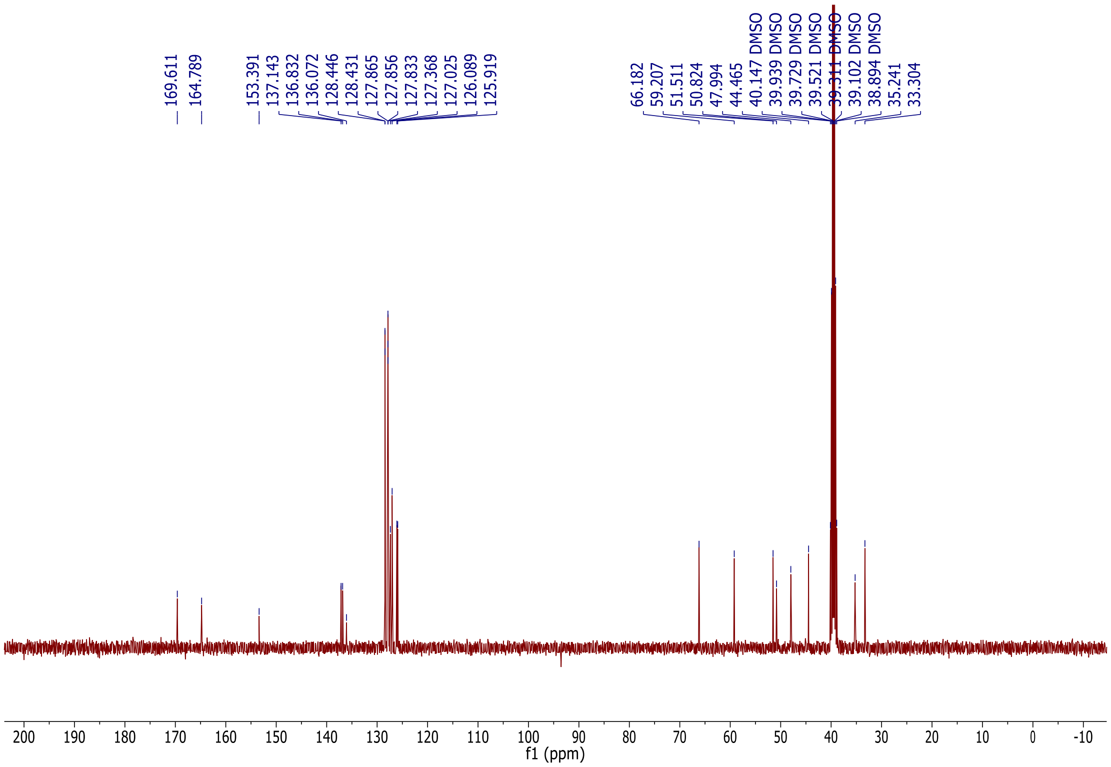

^{1}H-NMR (400 MHz, DMSO-d_6) and ^{13}C-NMR (100 MHz, DMSO-$d_{6,}$) 90 °C. Compound 21ab

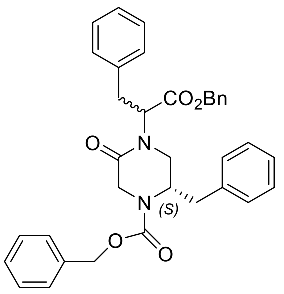

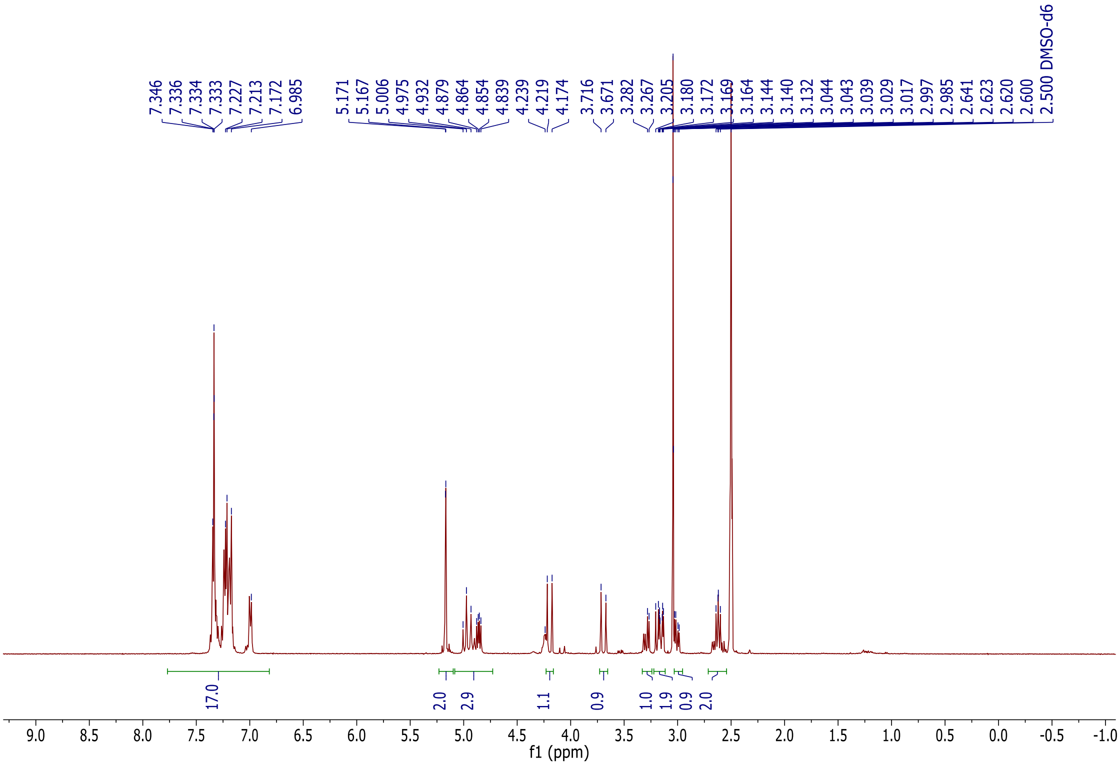

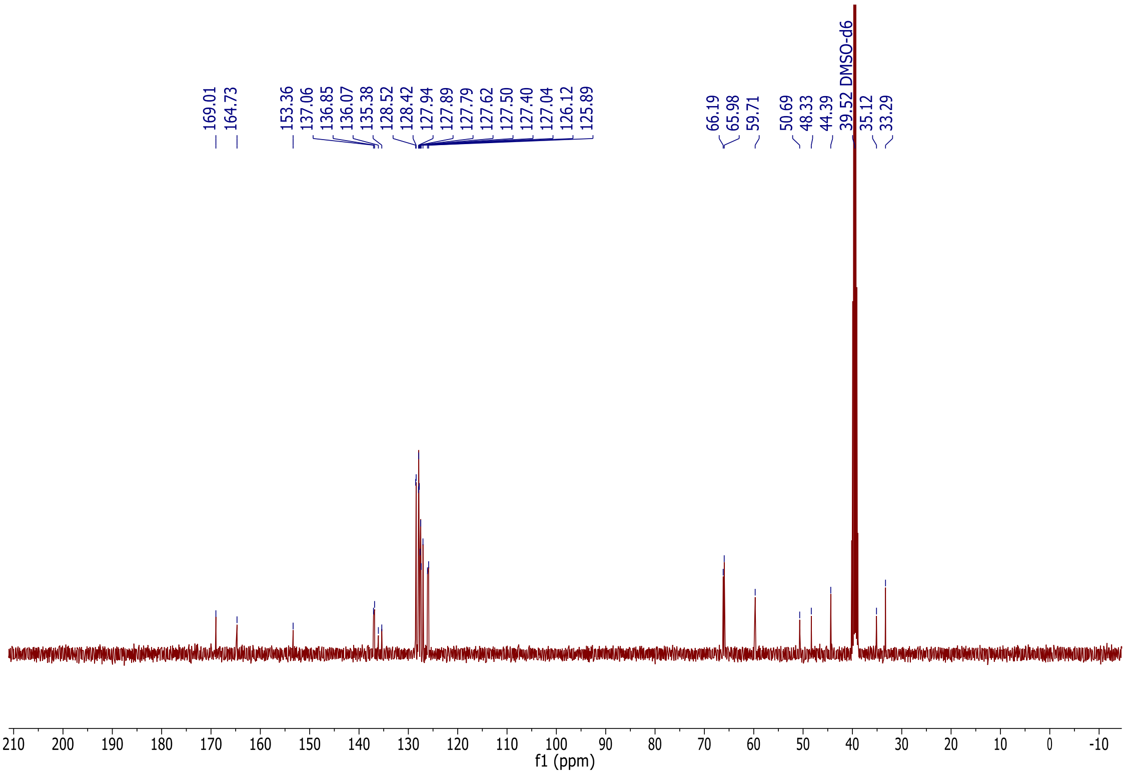

^{1}H-NMR (400 MHz, DMSO-d_6) and ^{13}C-NMR (75 MHz, DMSO-d_6). Compound 22

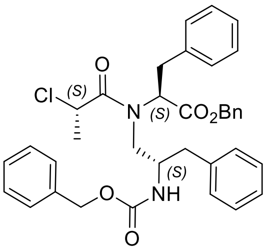

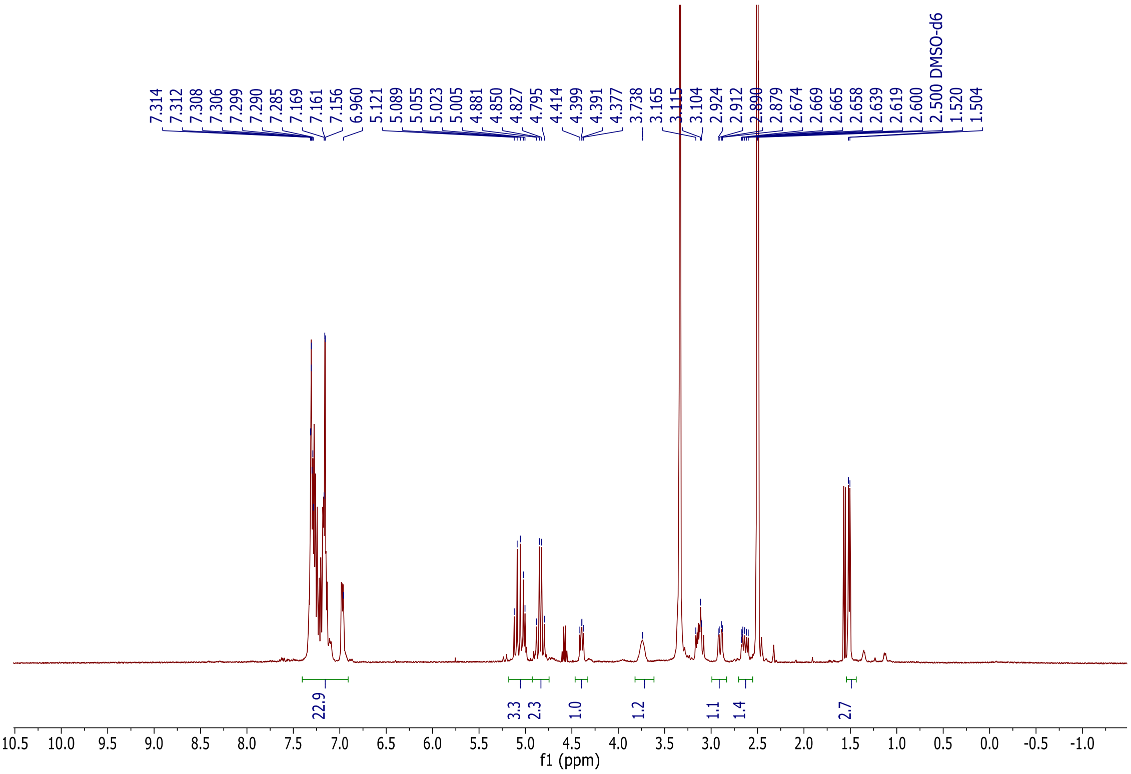

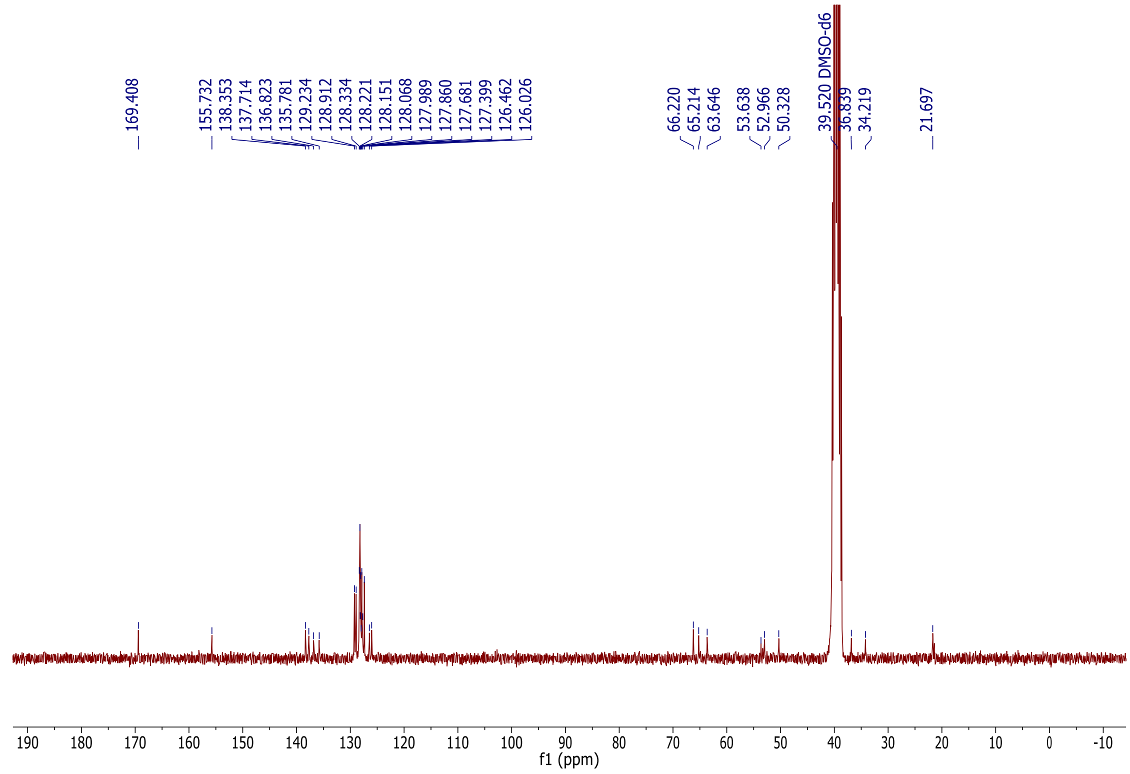

^{1}H-NMR (400 MHz, DMSO-d_6) and ^{13}C-NMR (75 MHz, DMSO-d_6). Compound 23

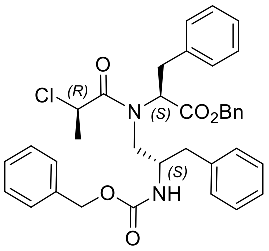

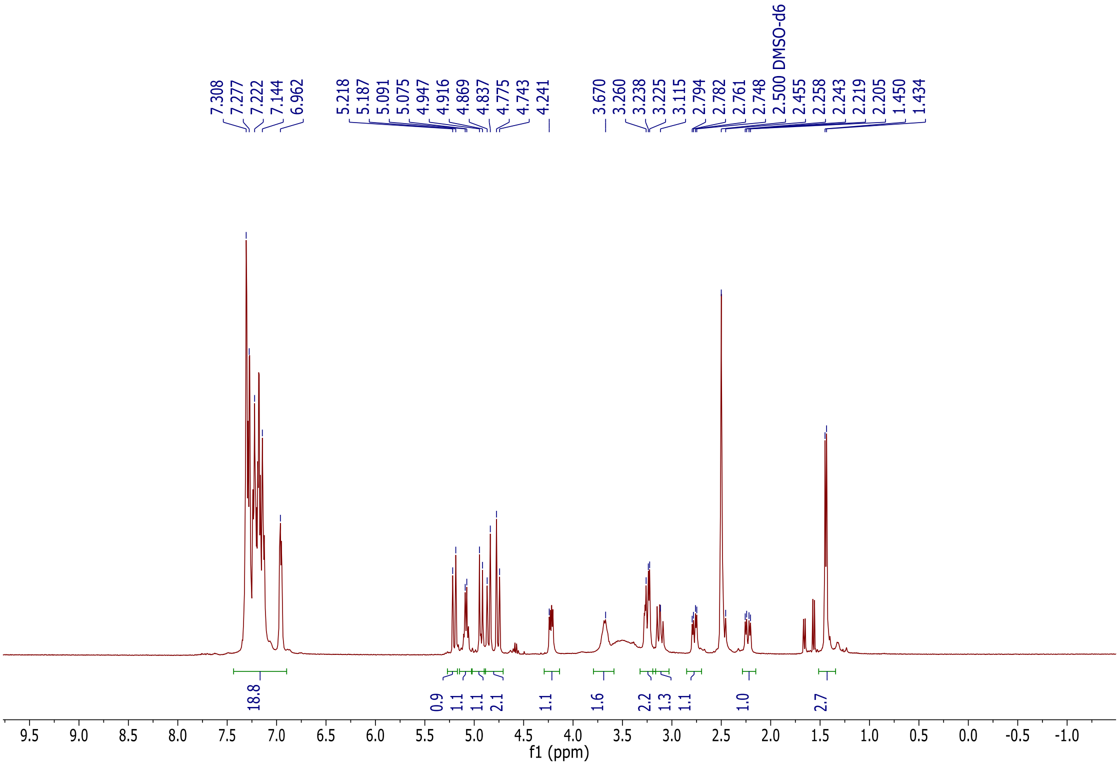

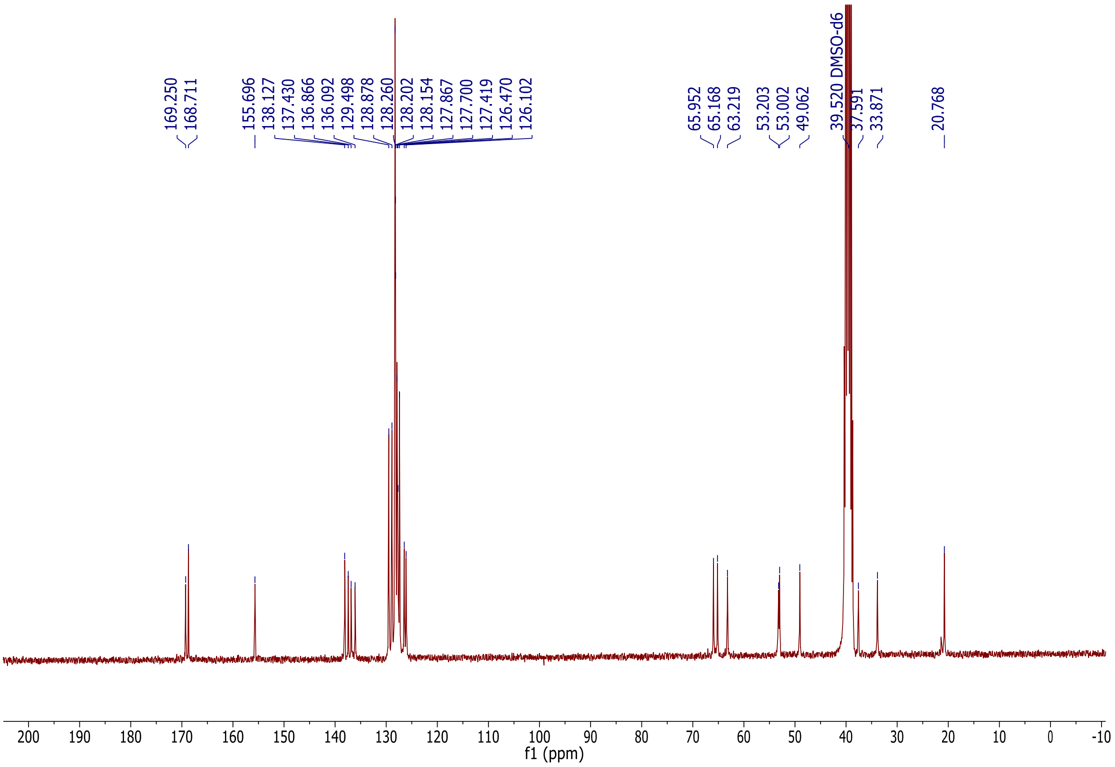

^{1}H-NMR (400 MHz, $CDCl_3$) and ^{13}C-NMR (75 MHz, $CDCl_3$). Compound 24a

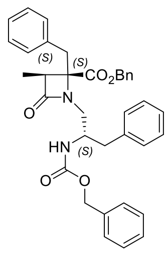

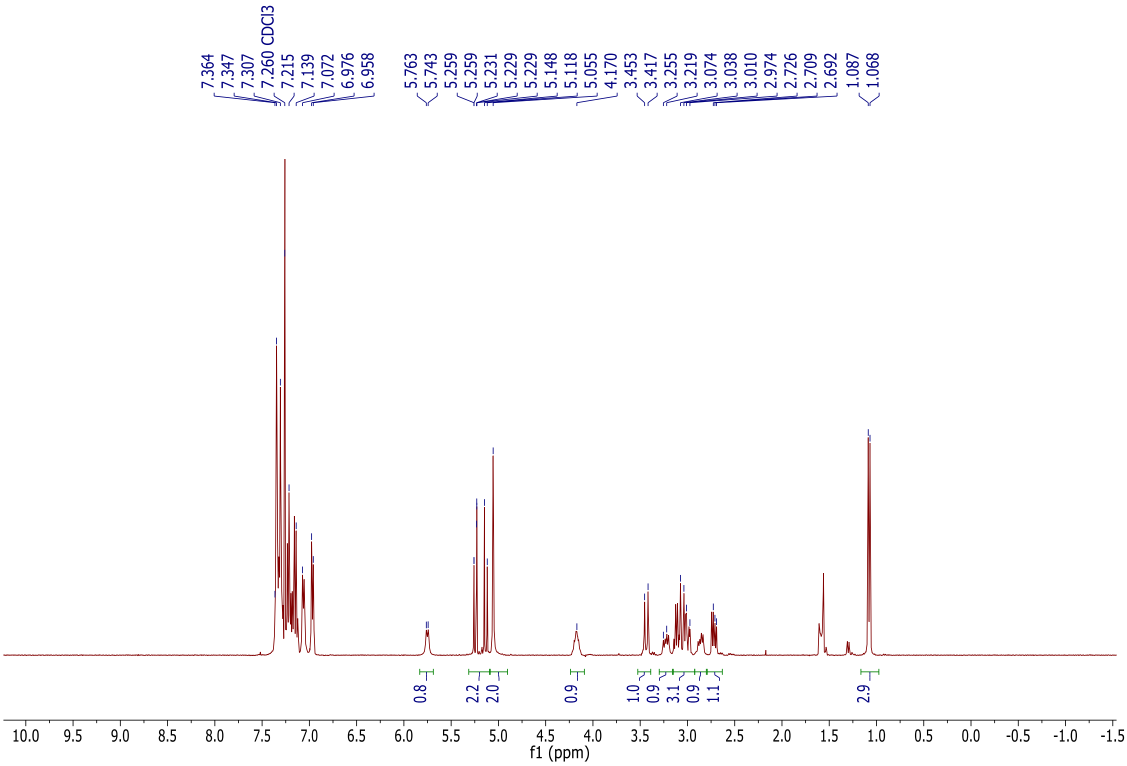

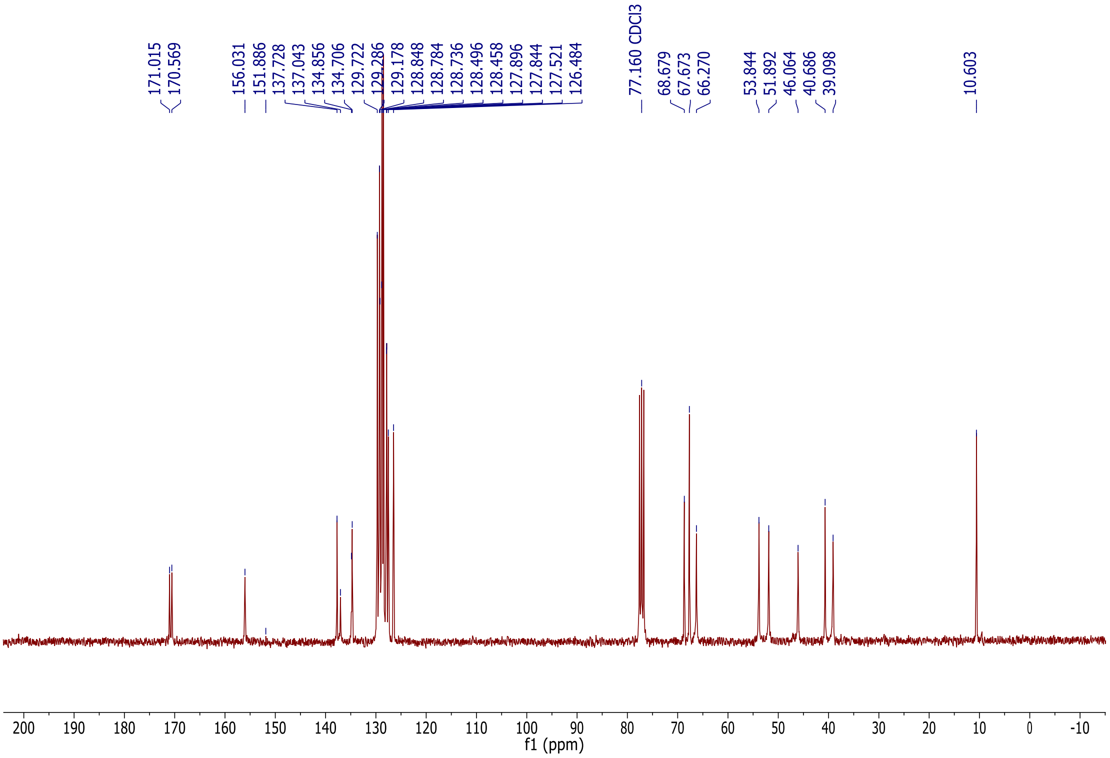

^{1}H-NMR (400 MHz, $CDCl_3$) and ^{13}C-NMR (75 MHz, $CDCl_3$). Compound 25ab

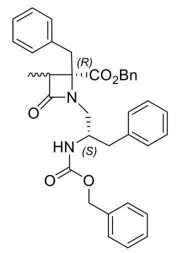

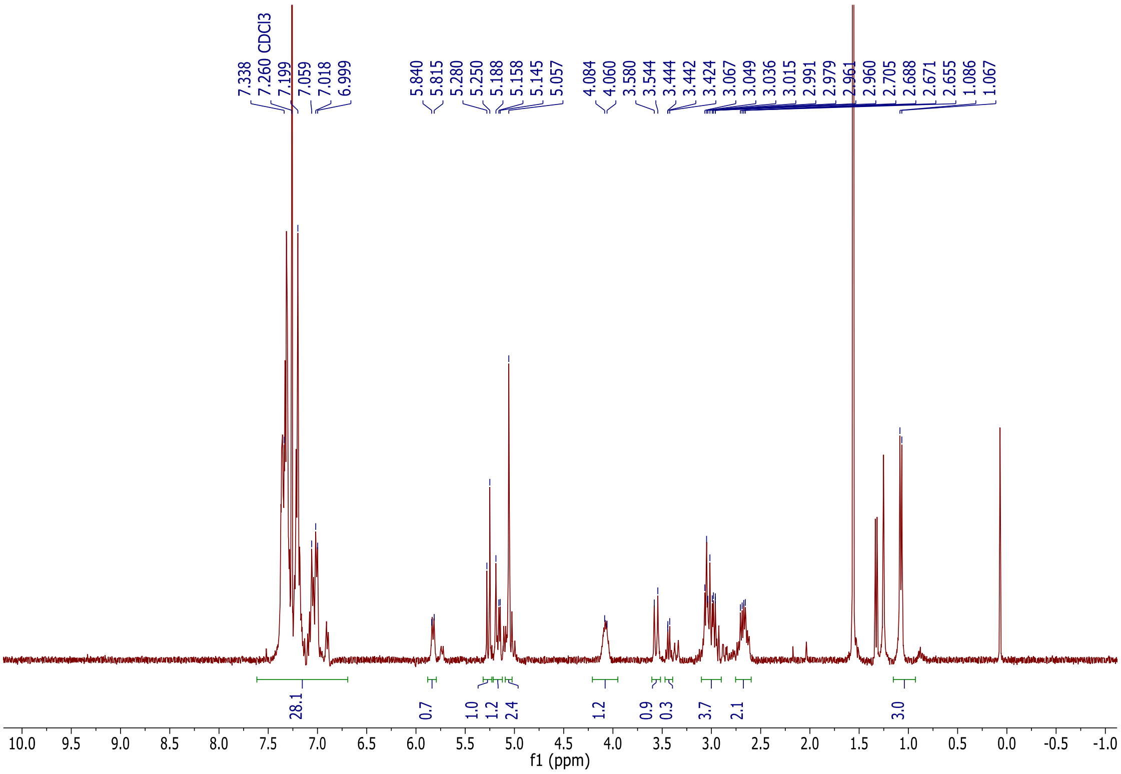

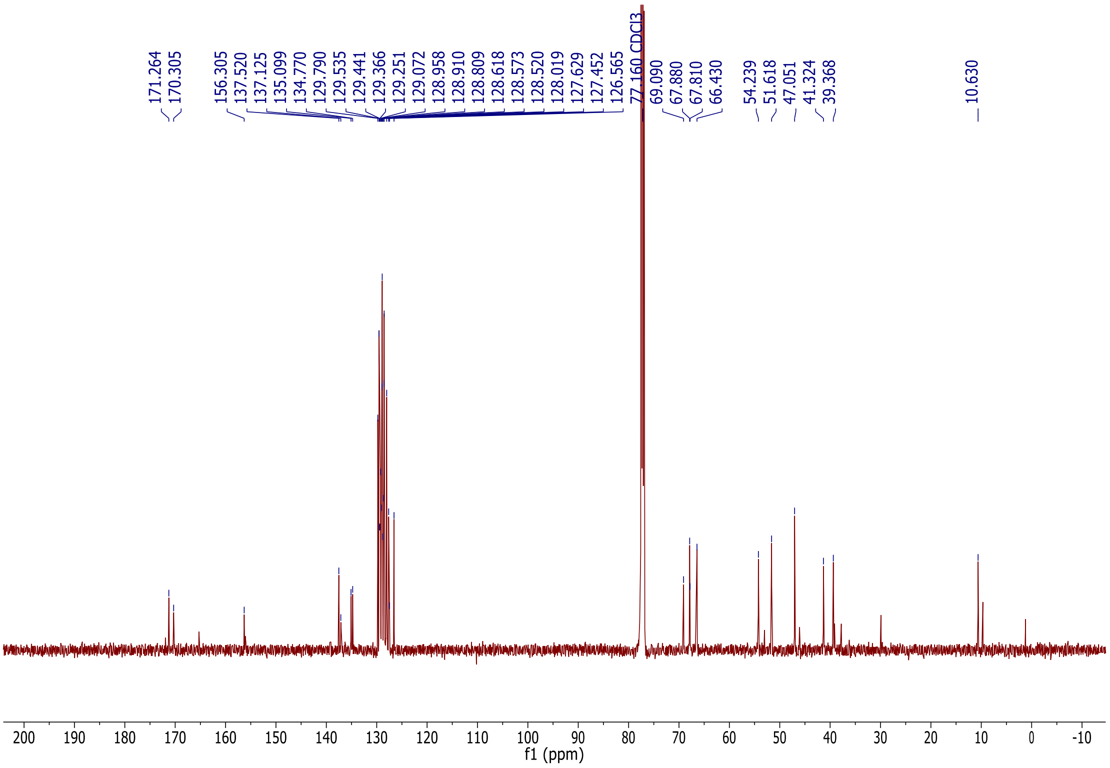

^{1}H-NMR (400 MHz, DMSO-d_6) and ^{13}C-NMR (100 MHz, DMSO-$d_{6,}$) 90 °C. Compound 26ab

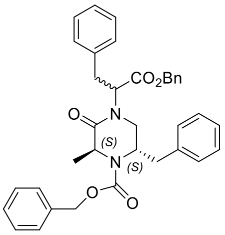

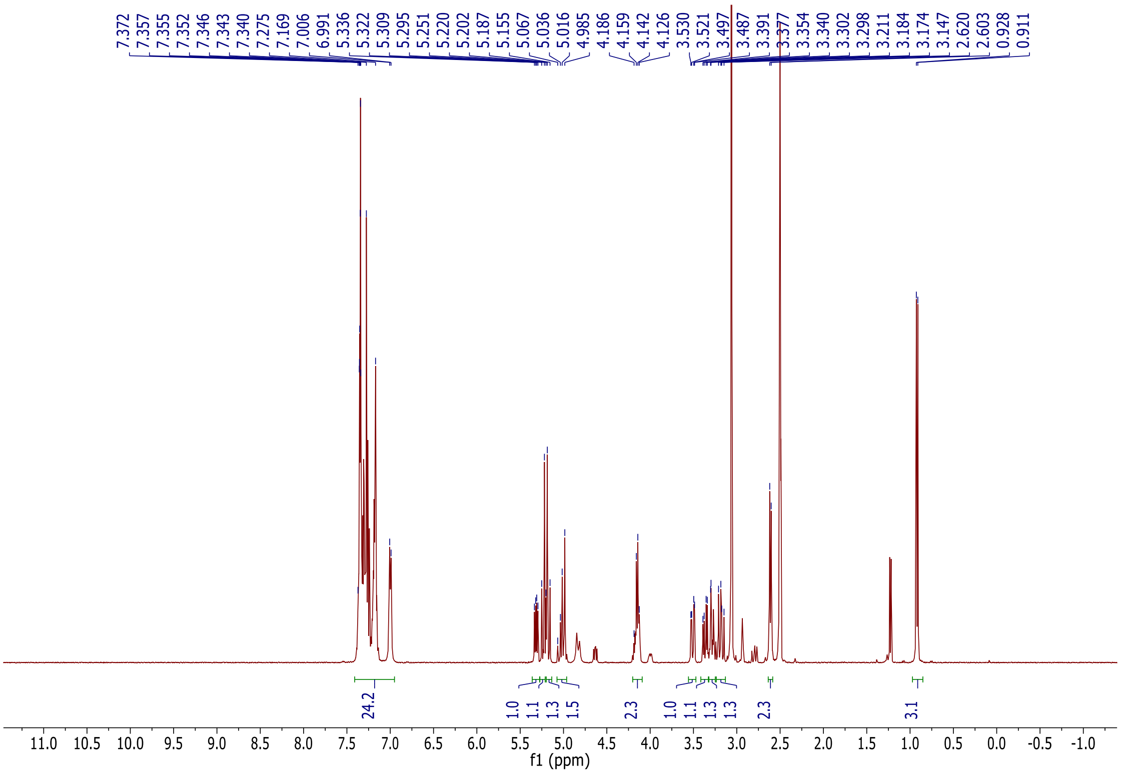

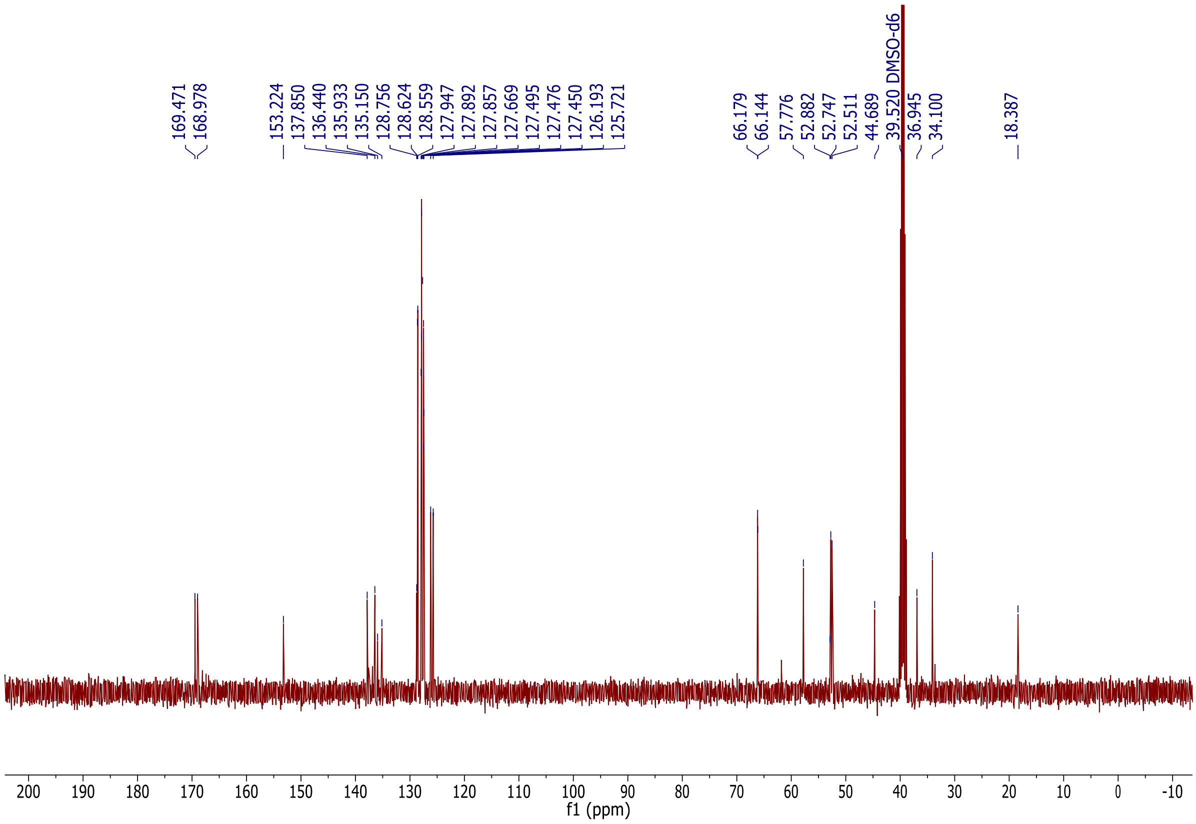

^{1}H-NMR (400 MHz, DMSO-d_6) and ^{13}C-NMR (75 MHz, DMSO-d_6). Compound 27

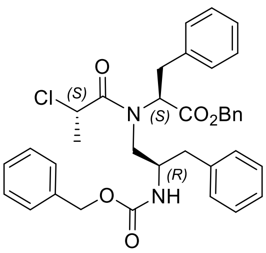

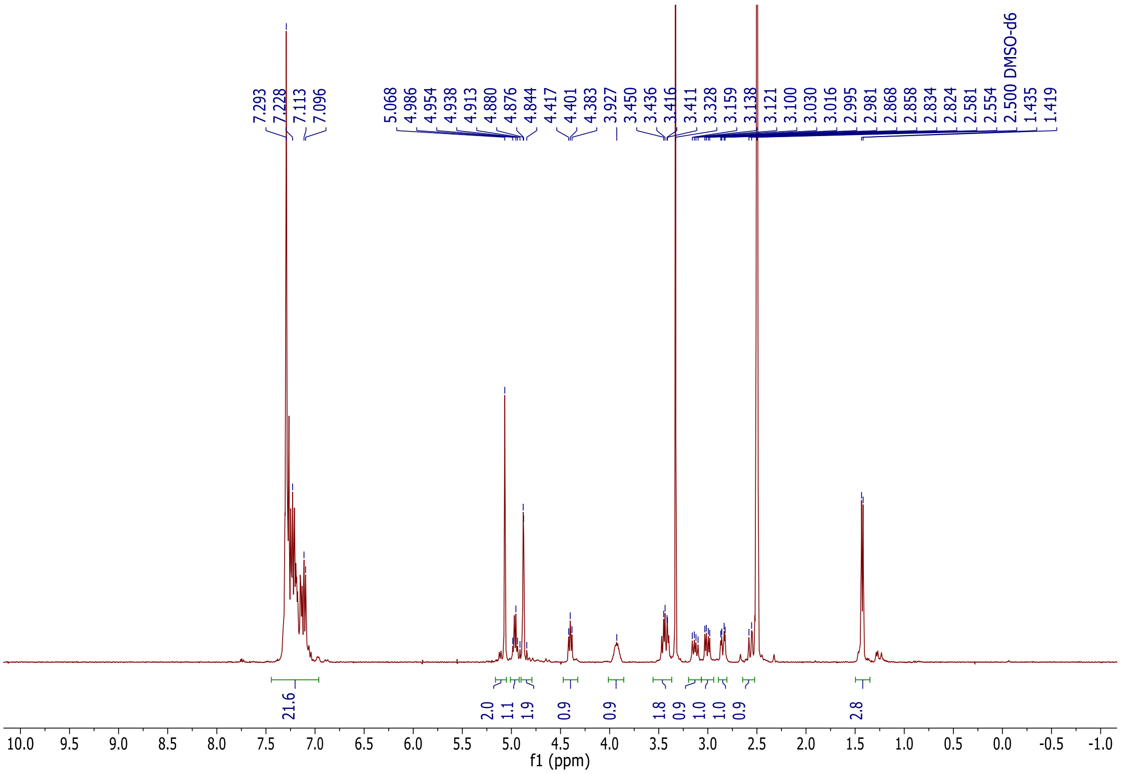

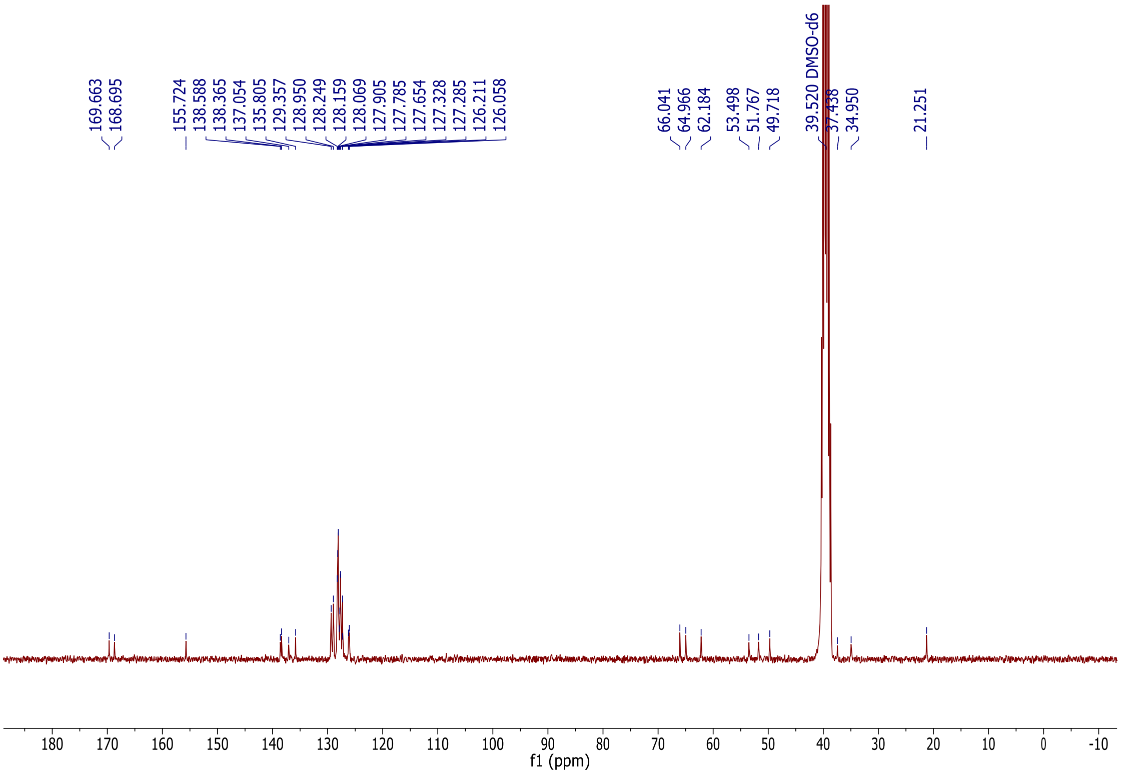

^{1}H-NMR (400 MHz, DMSO-d_6) and ^{13}C-NMR (75 MHz, DMSO-d_6). Compound 28

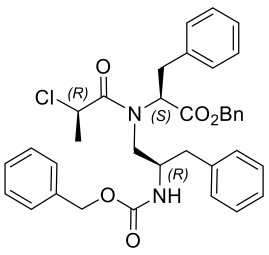

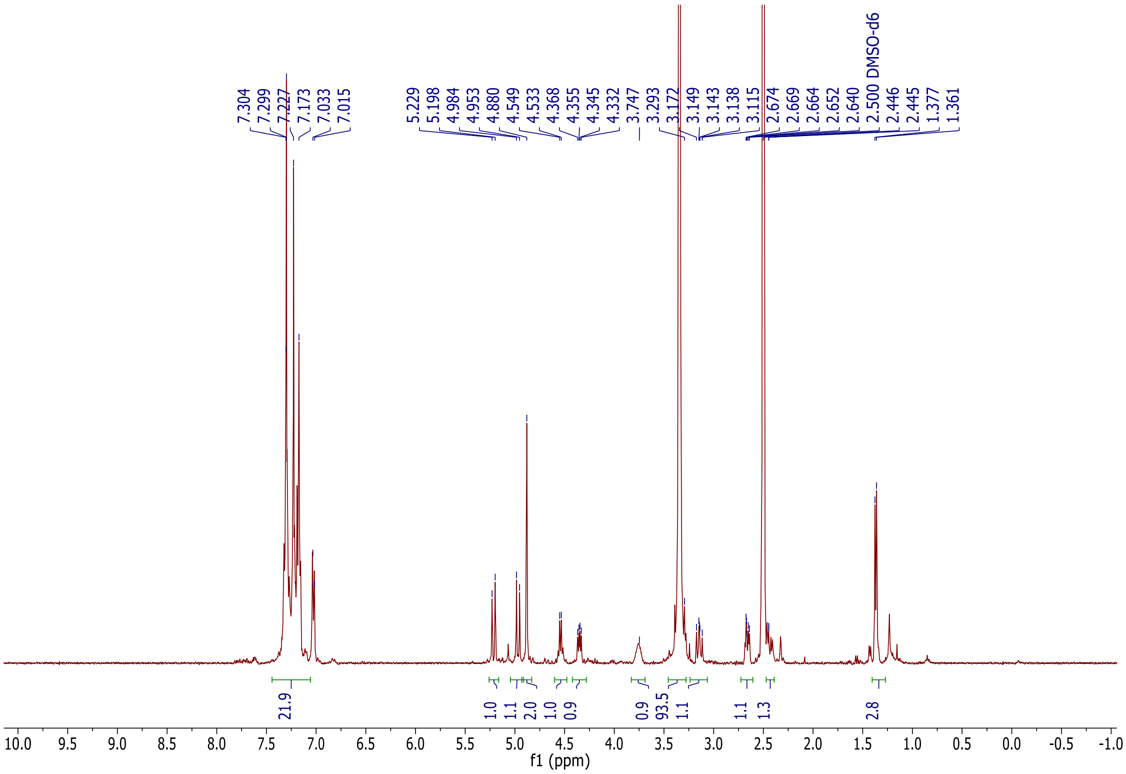

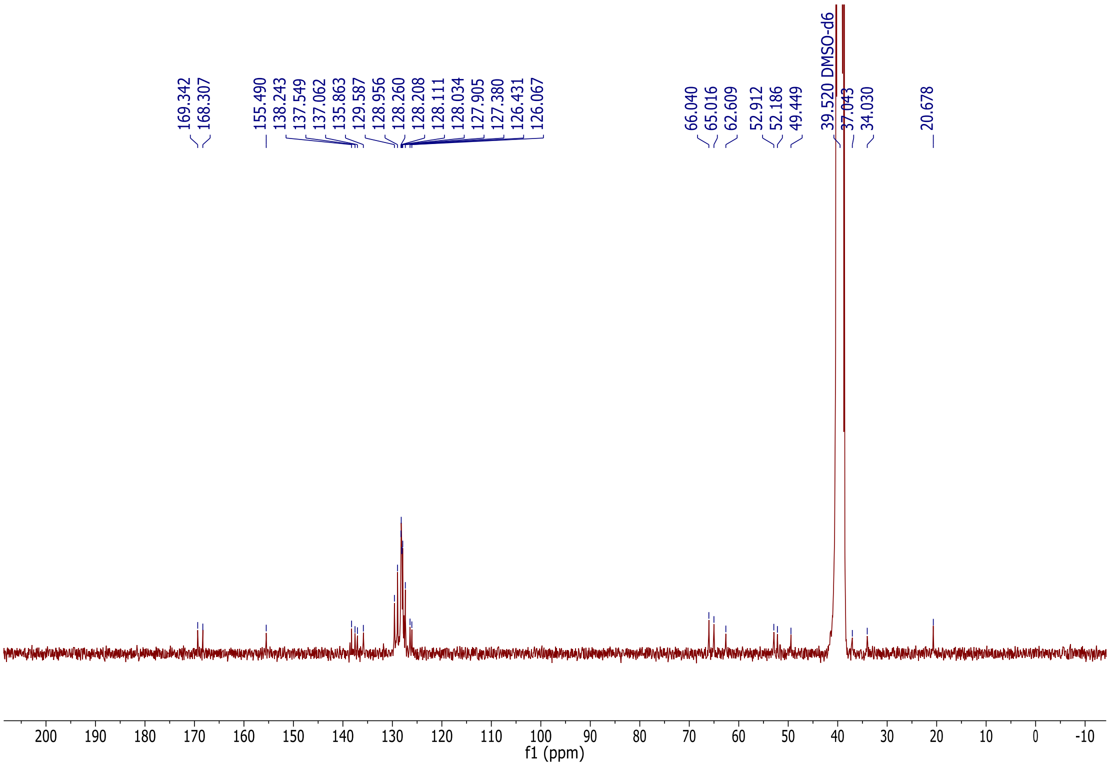

^{1}H-NMR (400 MHz, $CDCl_3$) and ^{13}C-NMR (75 MHz, $CDCl_3$) 29a

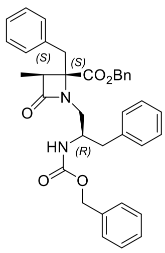

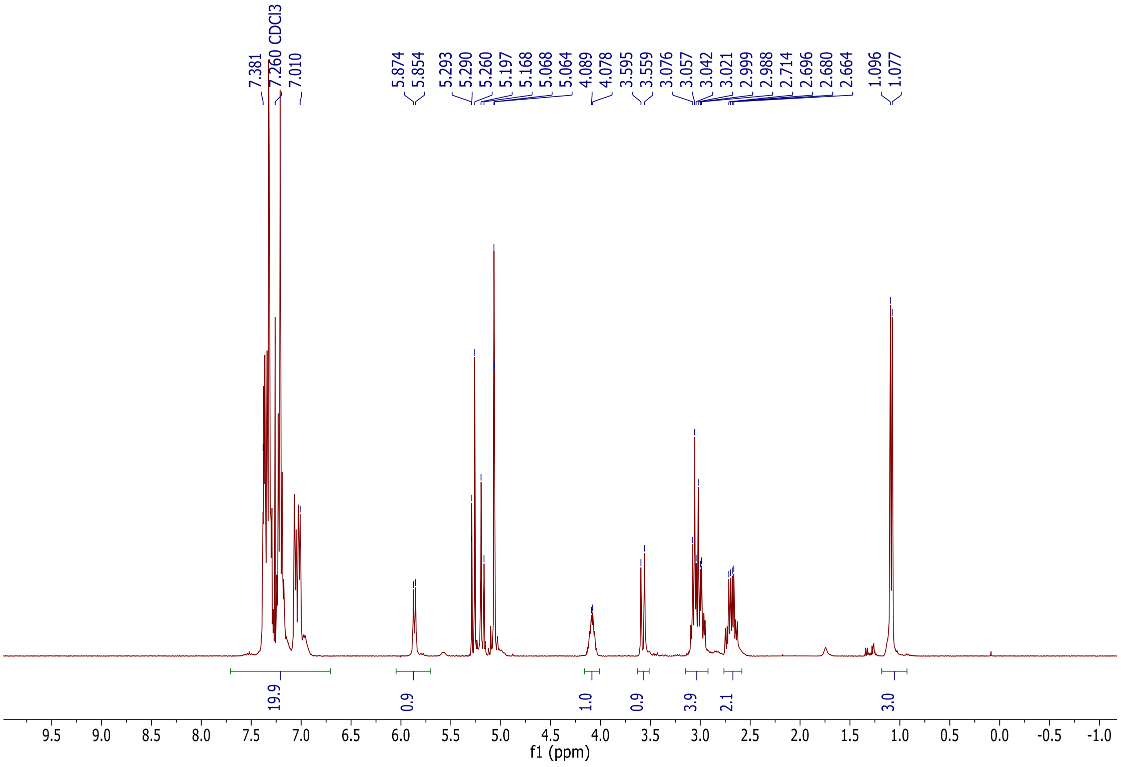

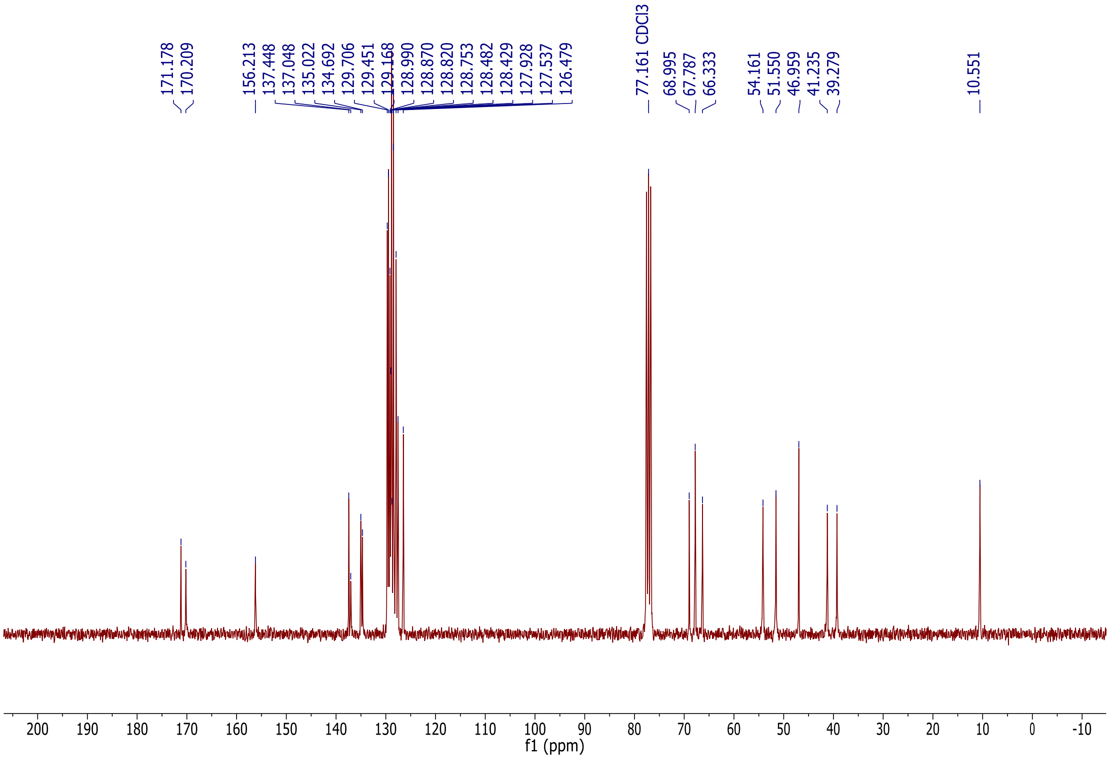

^{1}H-NMR (400 MHz, DMSO-d_6) and ^{13}C-NMR (100 MHz, DMSO-$d_{6,}$) 90 °C. Compound 30ab

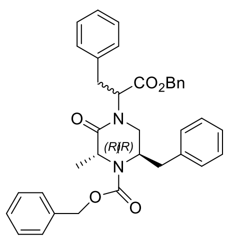

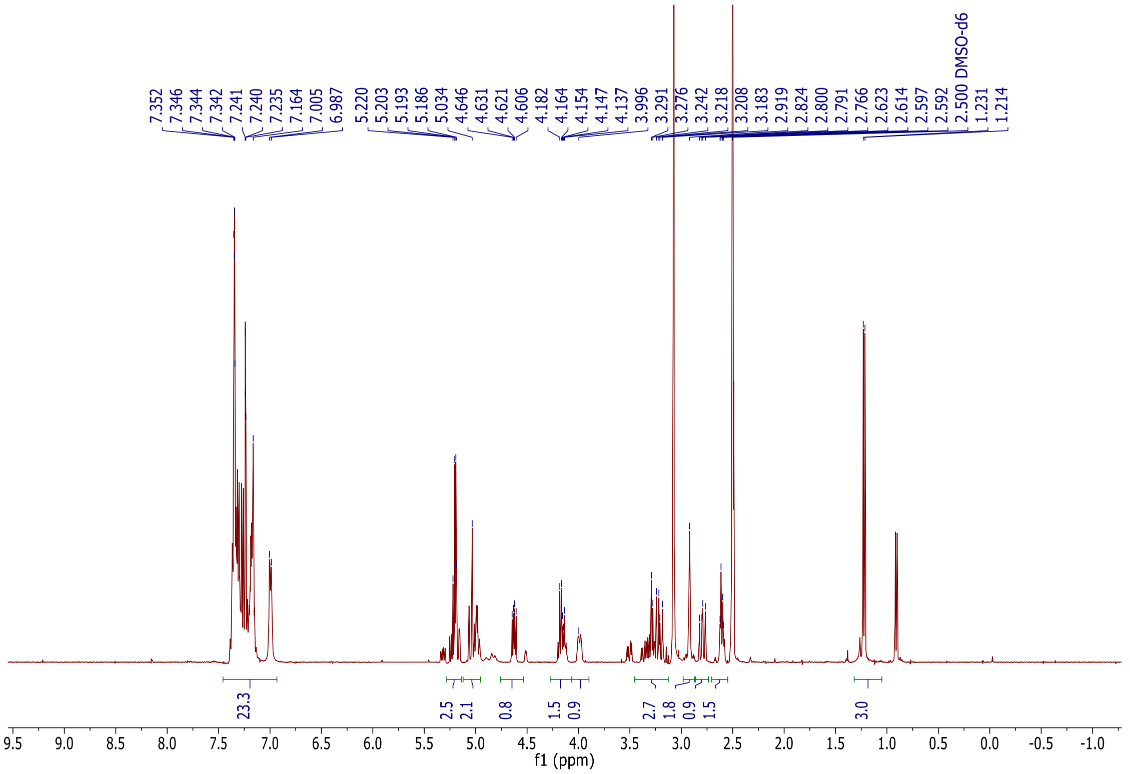

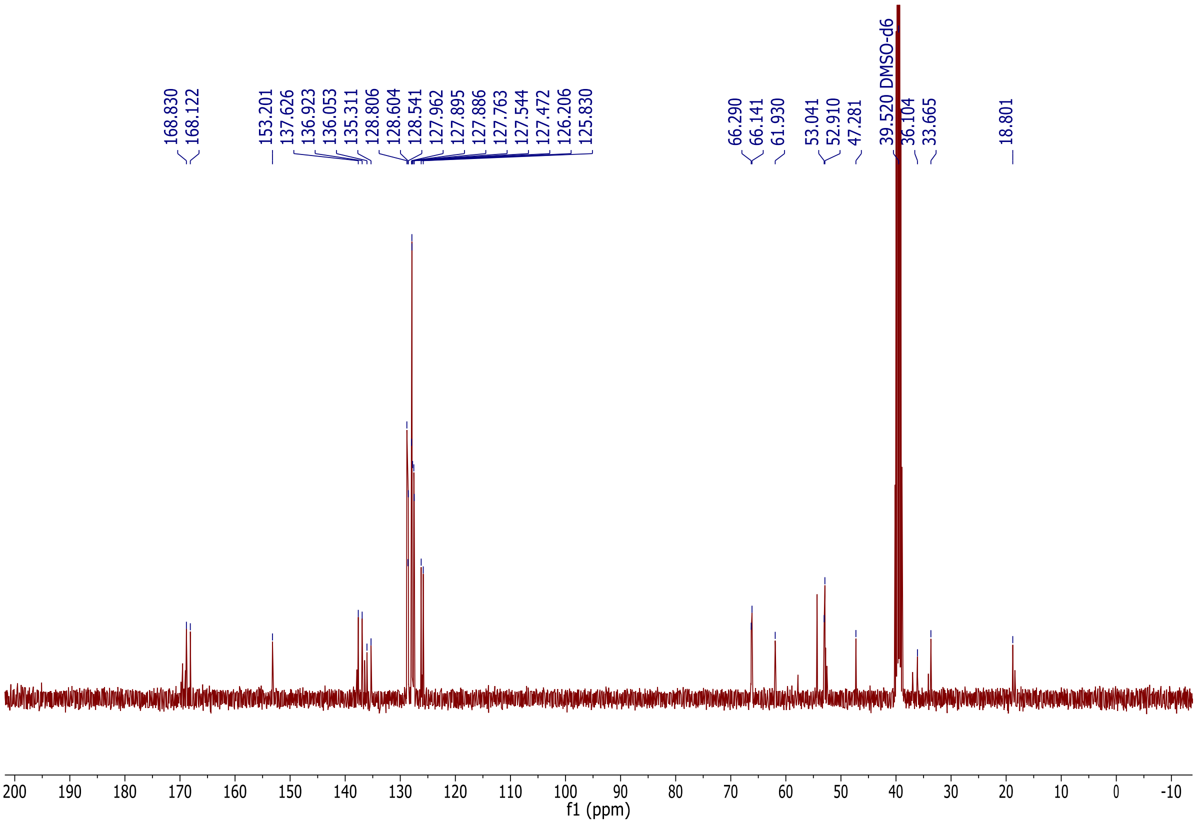

^{1}H-NMR (400 MHz, $CDCl_3$) and ^{13}C-NMR (75 MHz, $CDCl_3$). Compound 31ab

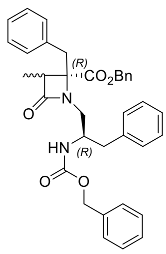

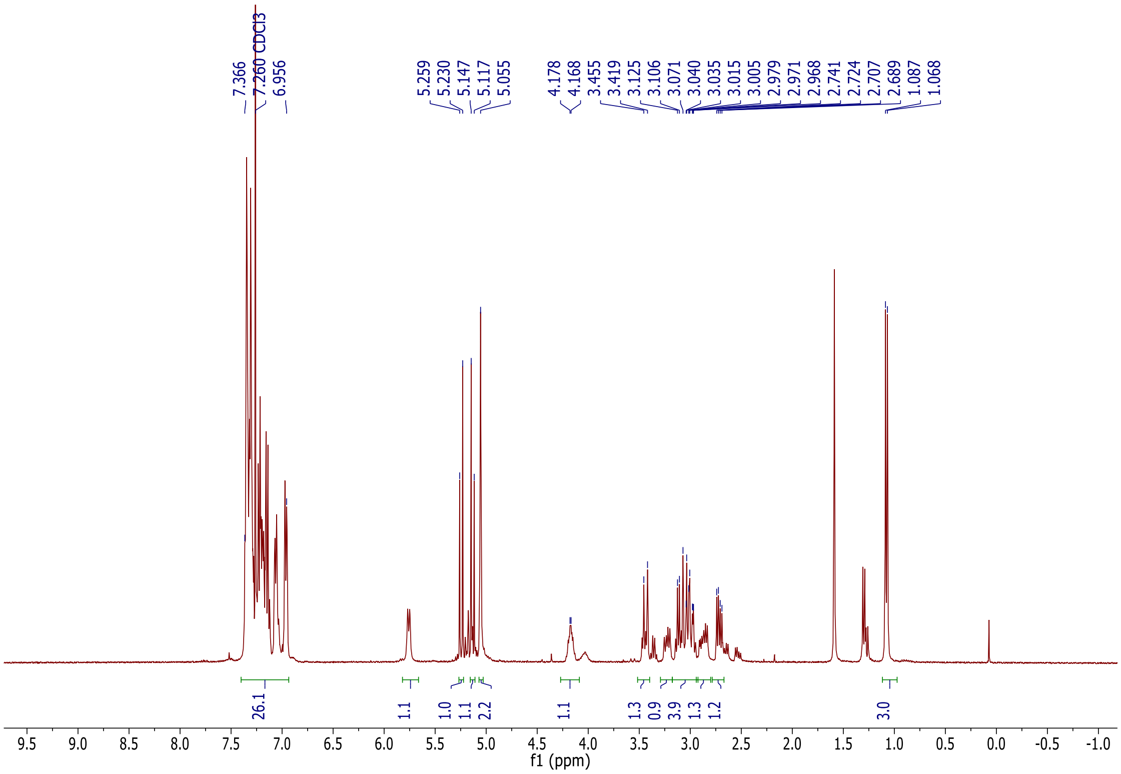

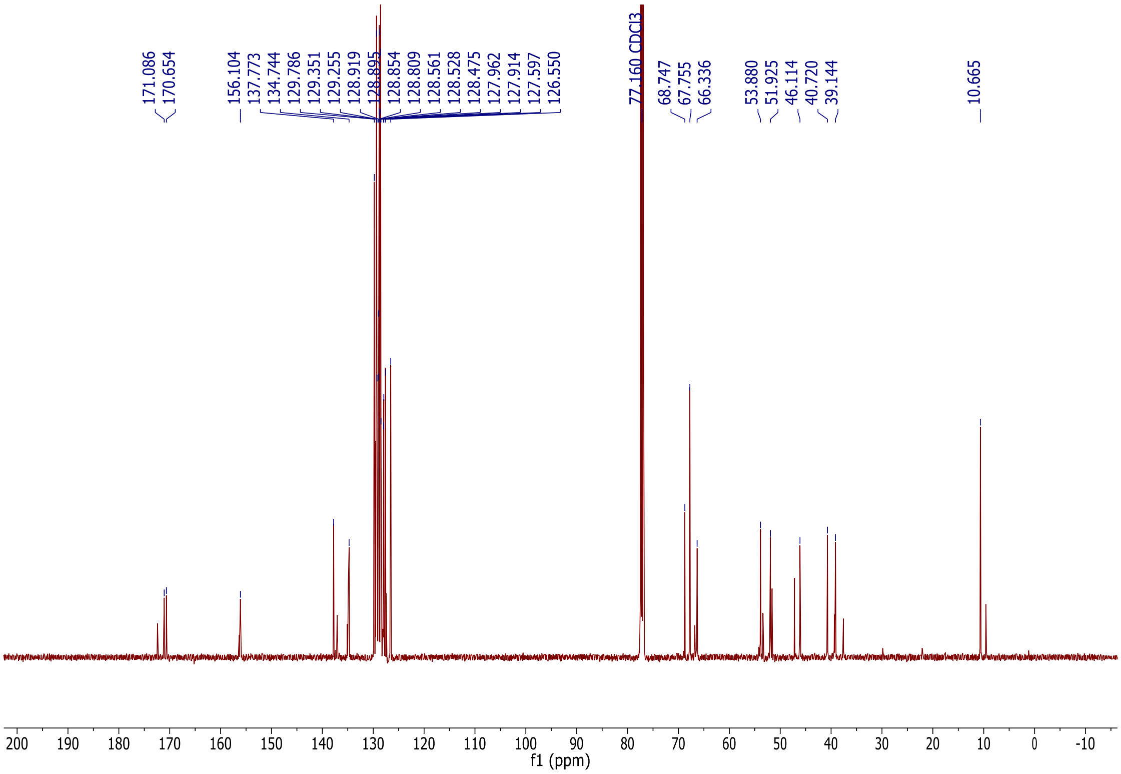

^{1}H-NMR (400 MHz, DMSO-d_6) and ^{13}C-NMR (100 MHz, DMSO-$d_{6,}$) 90 °C. Compound 32ab

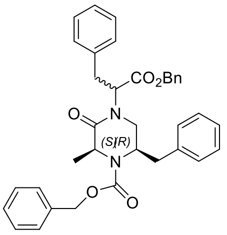

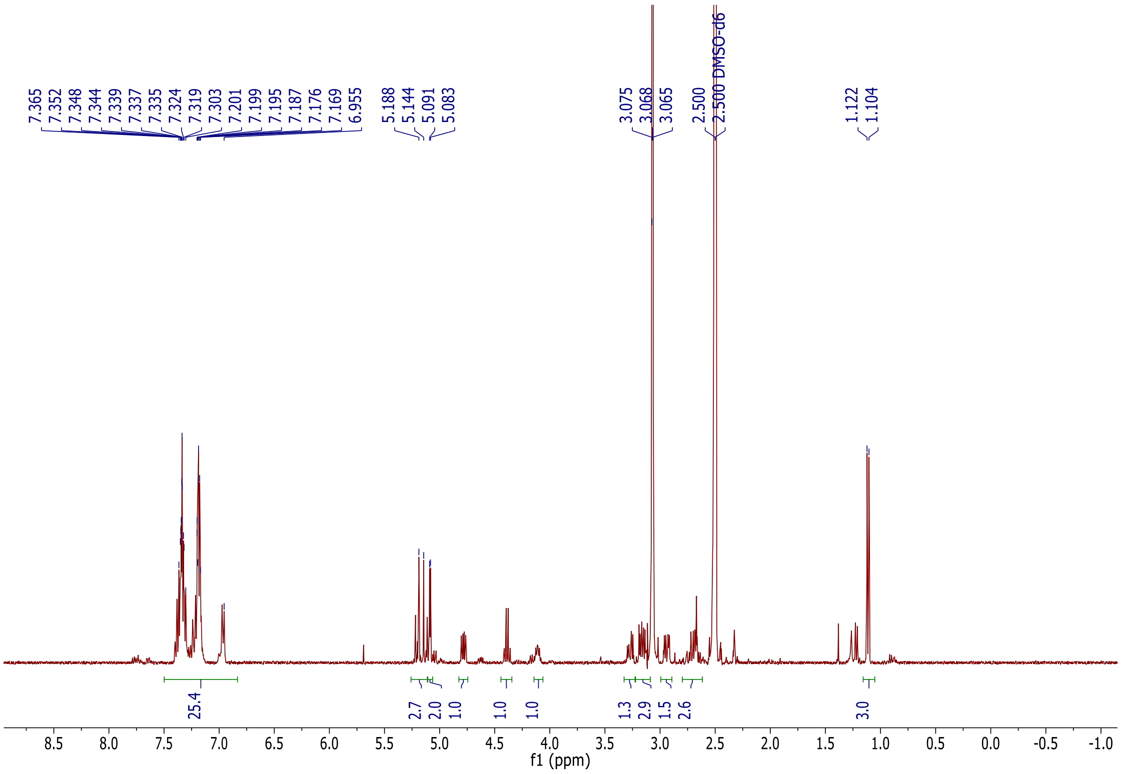

^{1}H-NMR (400 MHz, $CDCl_3$) and ^{13}C-NMR (75 MHz, $CDCl_3$). Compound 34

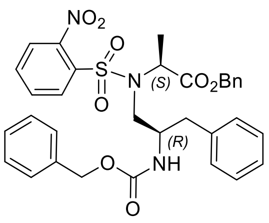

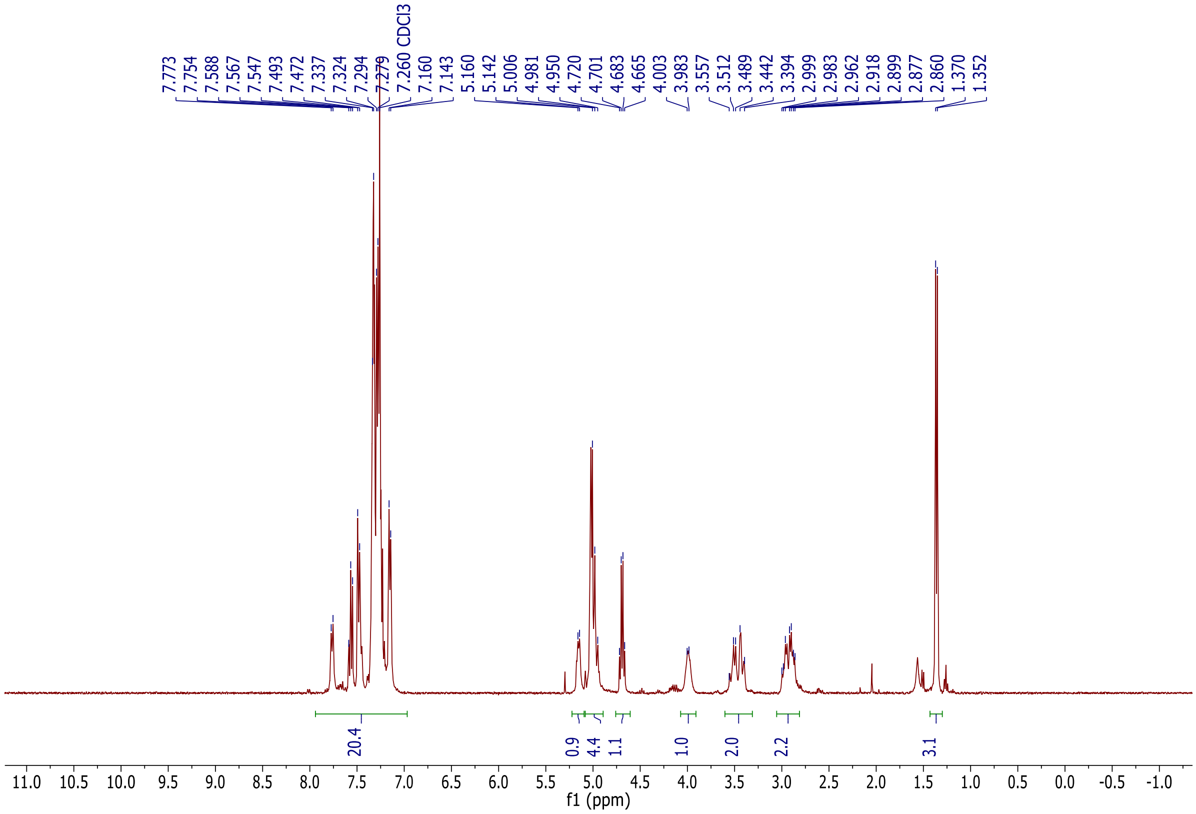

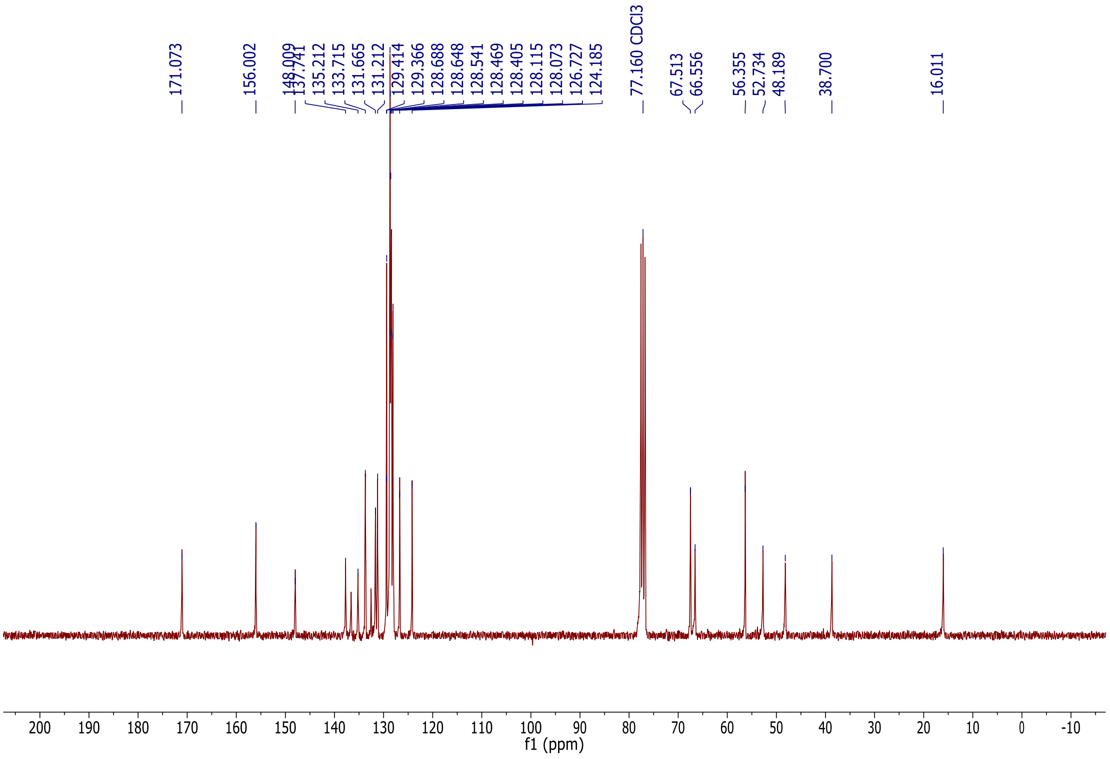

^{1}H-NMR (400 MHz, $CDCl_3$) and ^{13}C-NMR (75 MHz, $CDCl_3$). Compound 35

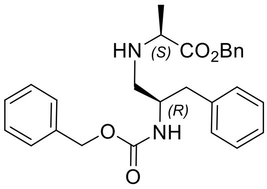

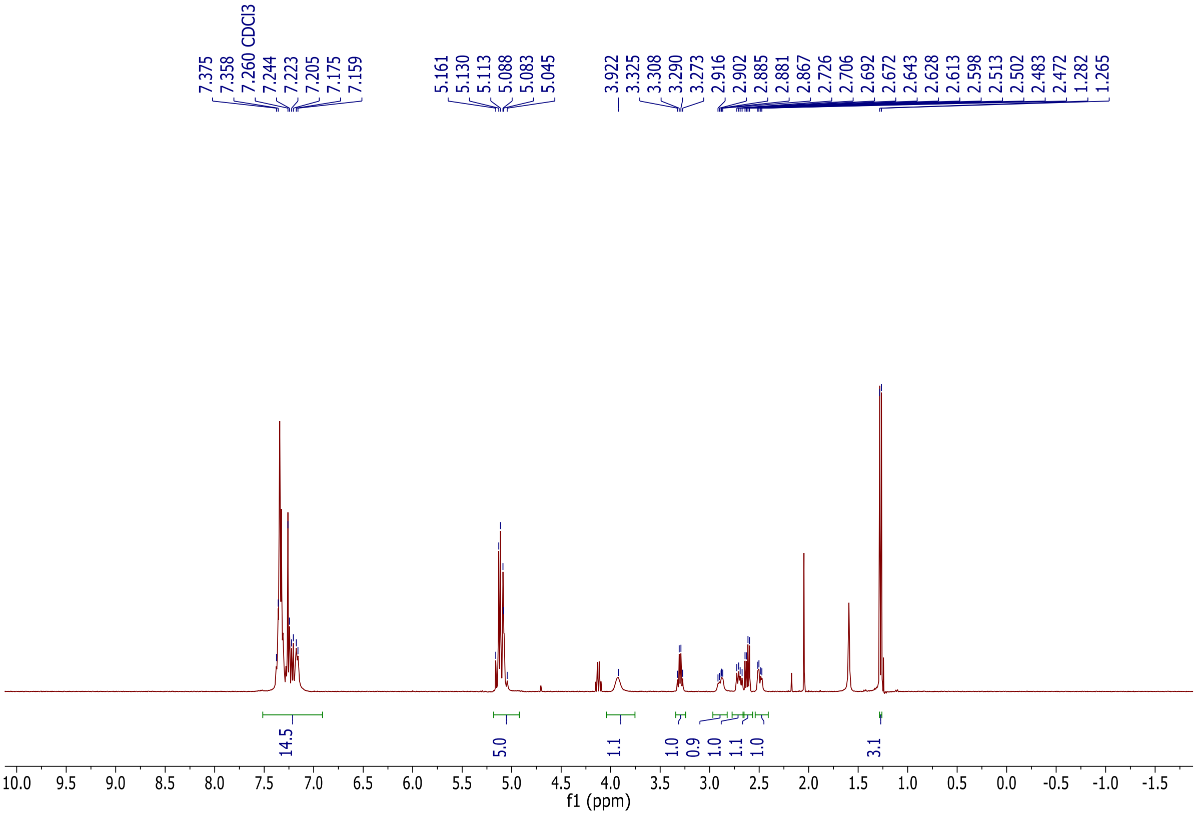

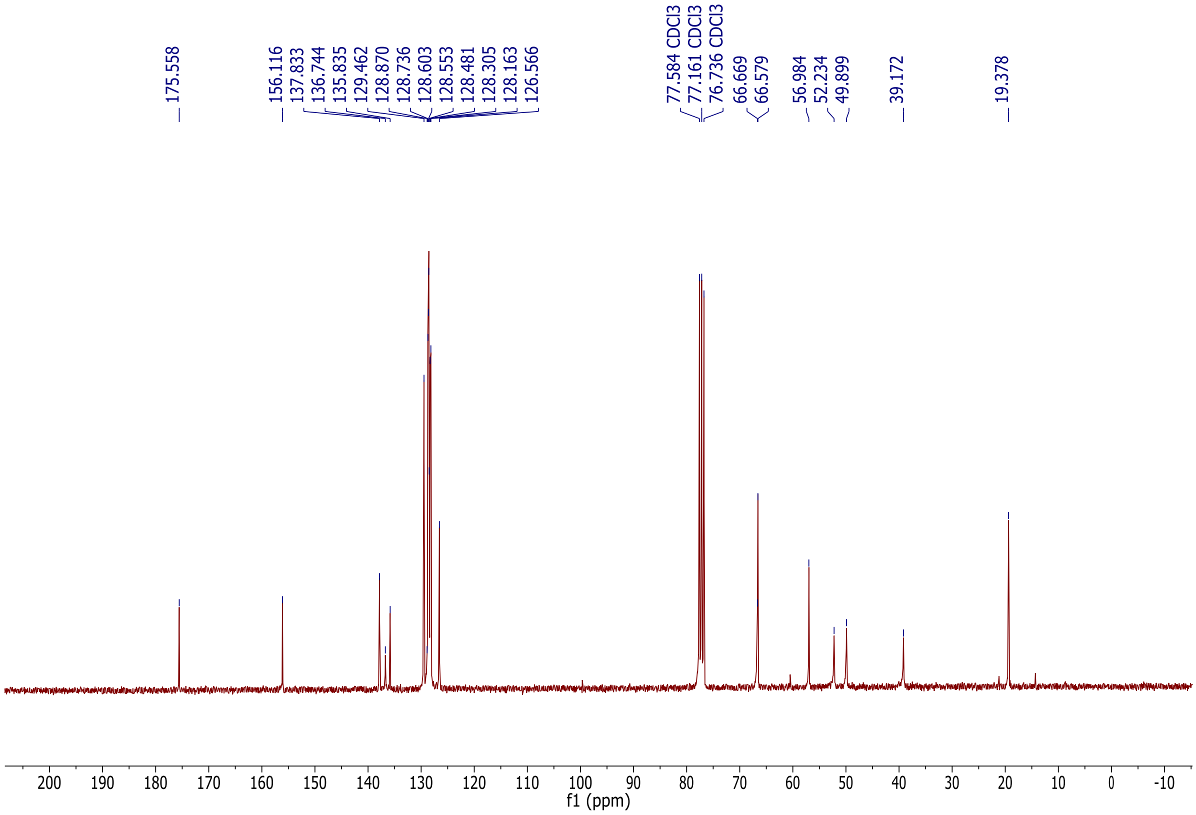

^{1}H-NMR (400 MHz, DMSO-d_6) and ^{13}C-NMR (75 MHz, DMSO-d_6). Compound 36

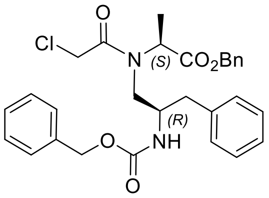

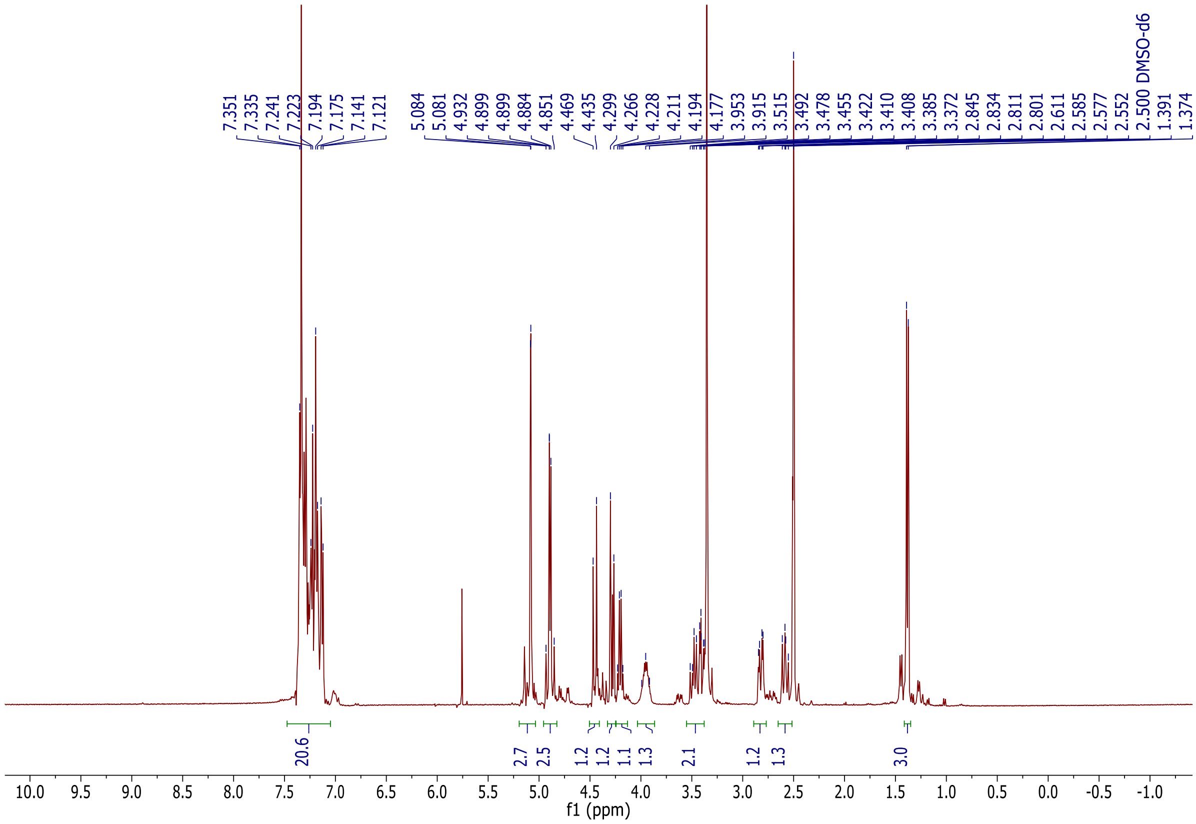

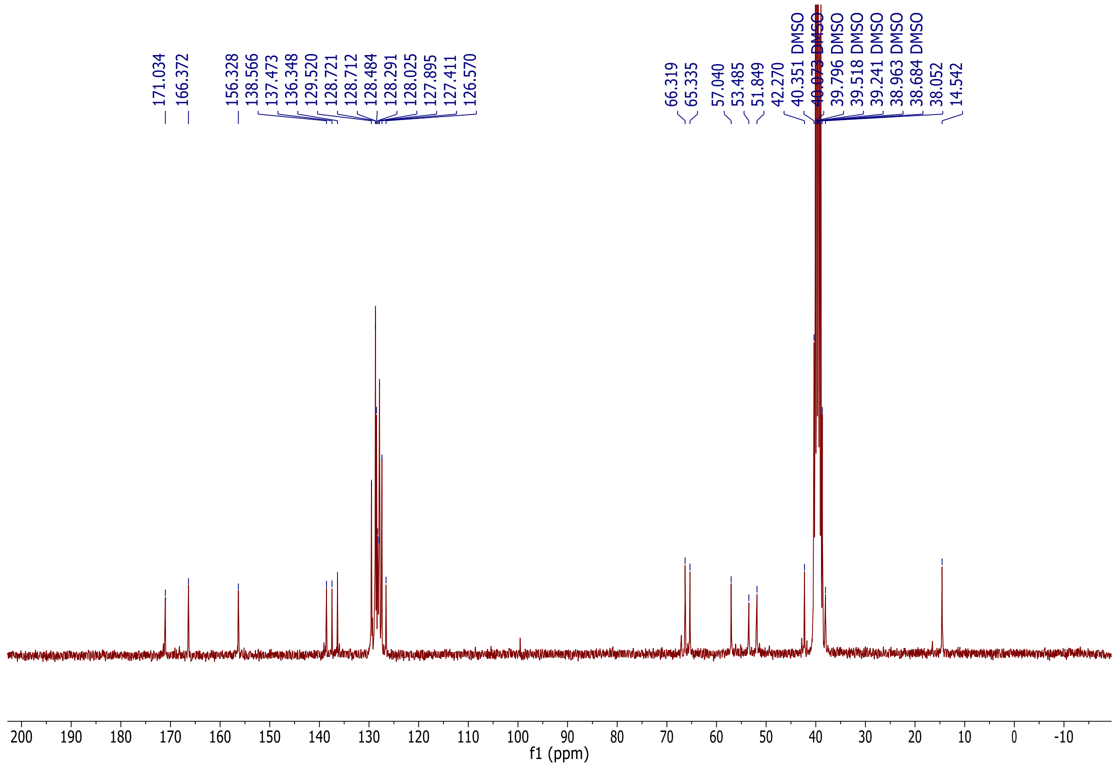

^{1}H-NMR (400 MHz, DMSO-d_6) and ^{13}C-NMR (75 MHz, DMSO-d_6). Compound 37

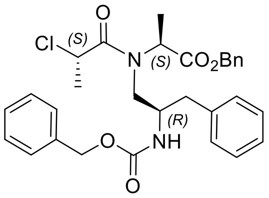

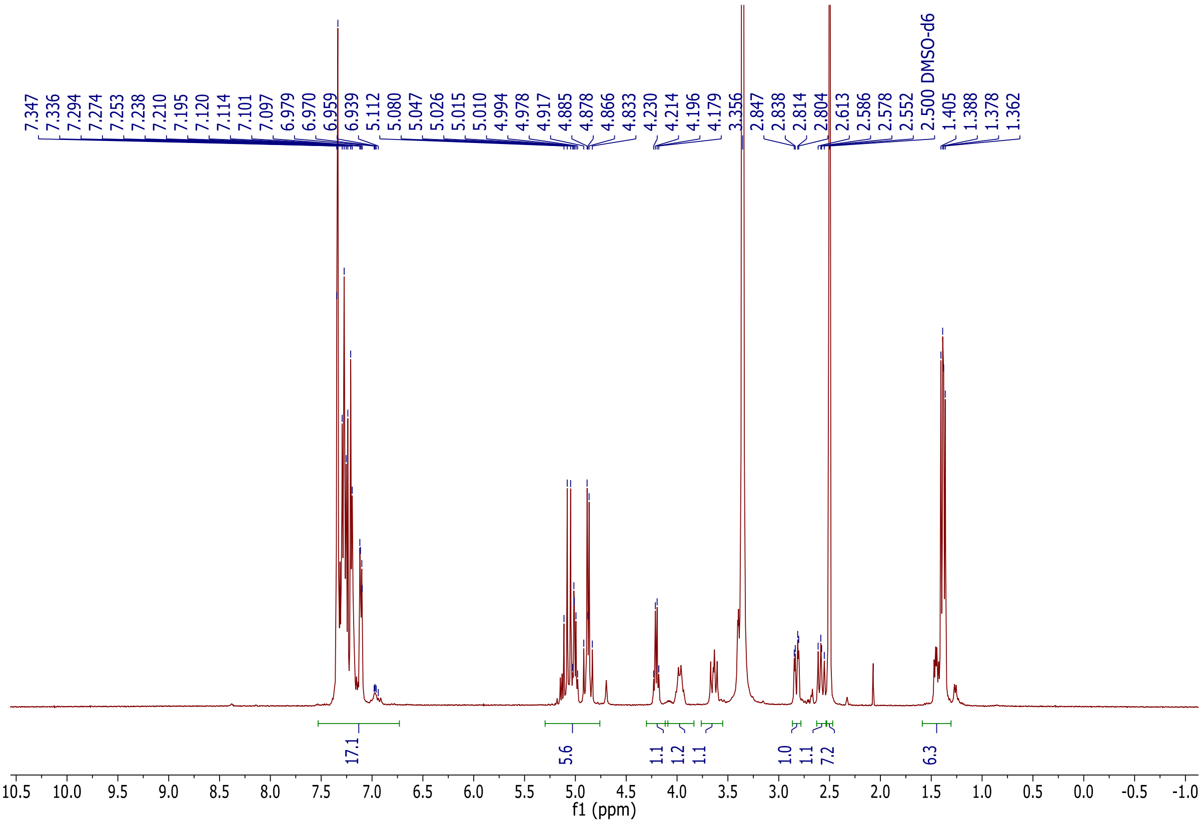

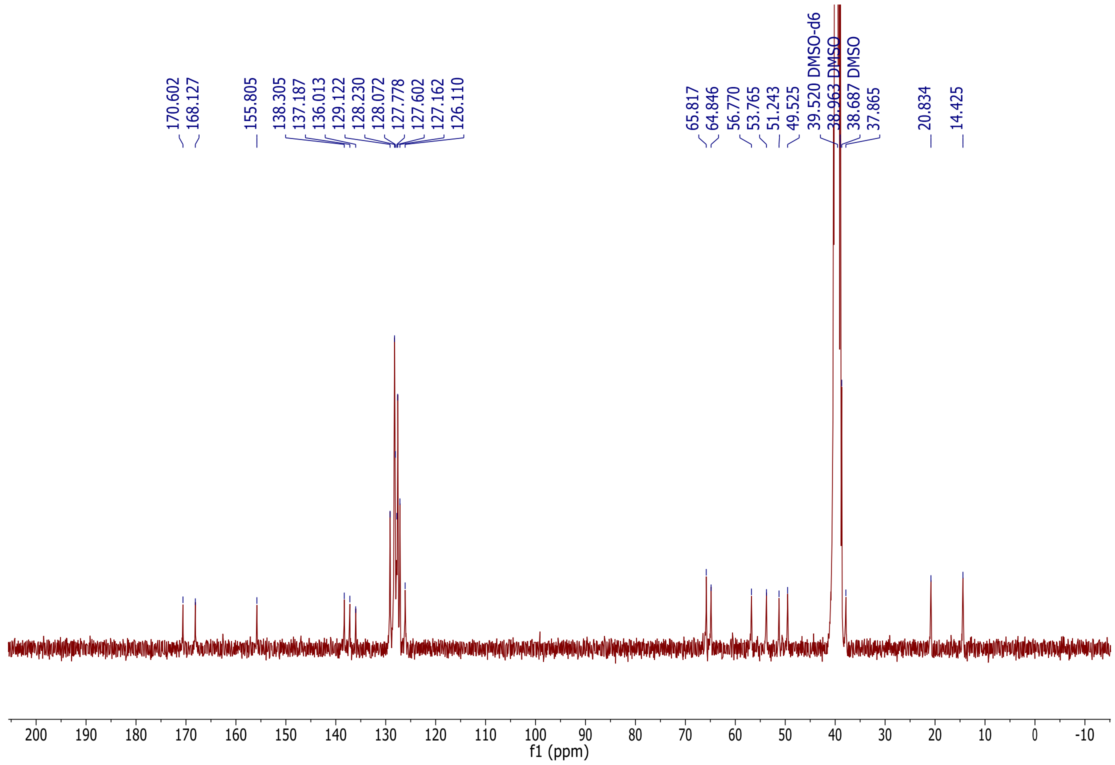

^{1}H-NMR (400 MHz, DMSO-d_6) and ^{13}C-NMR (100 MHz, DMSO-$d_{6,}$) 90 °C. Compound 38ab

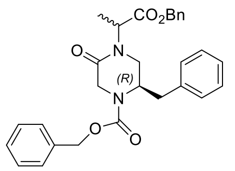

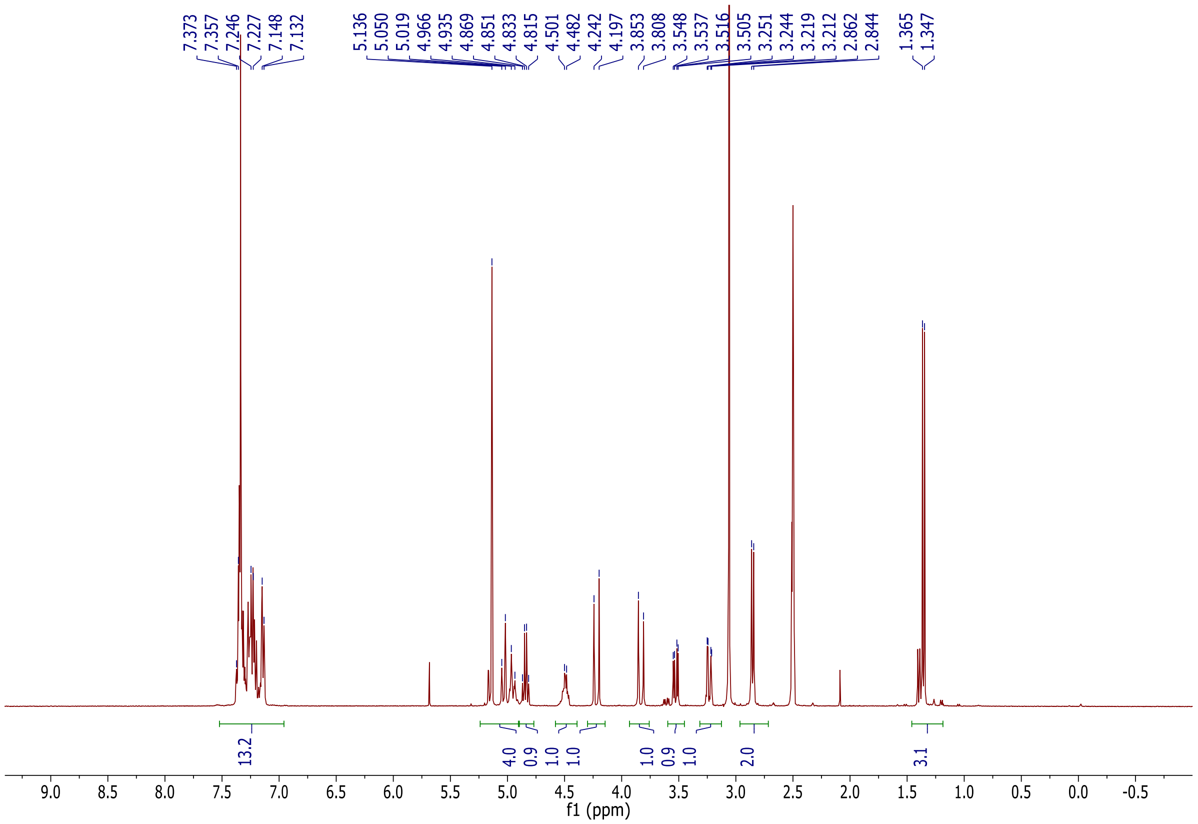

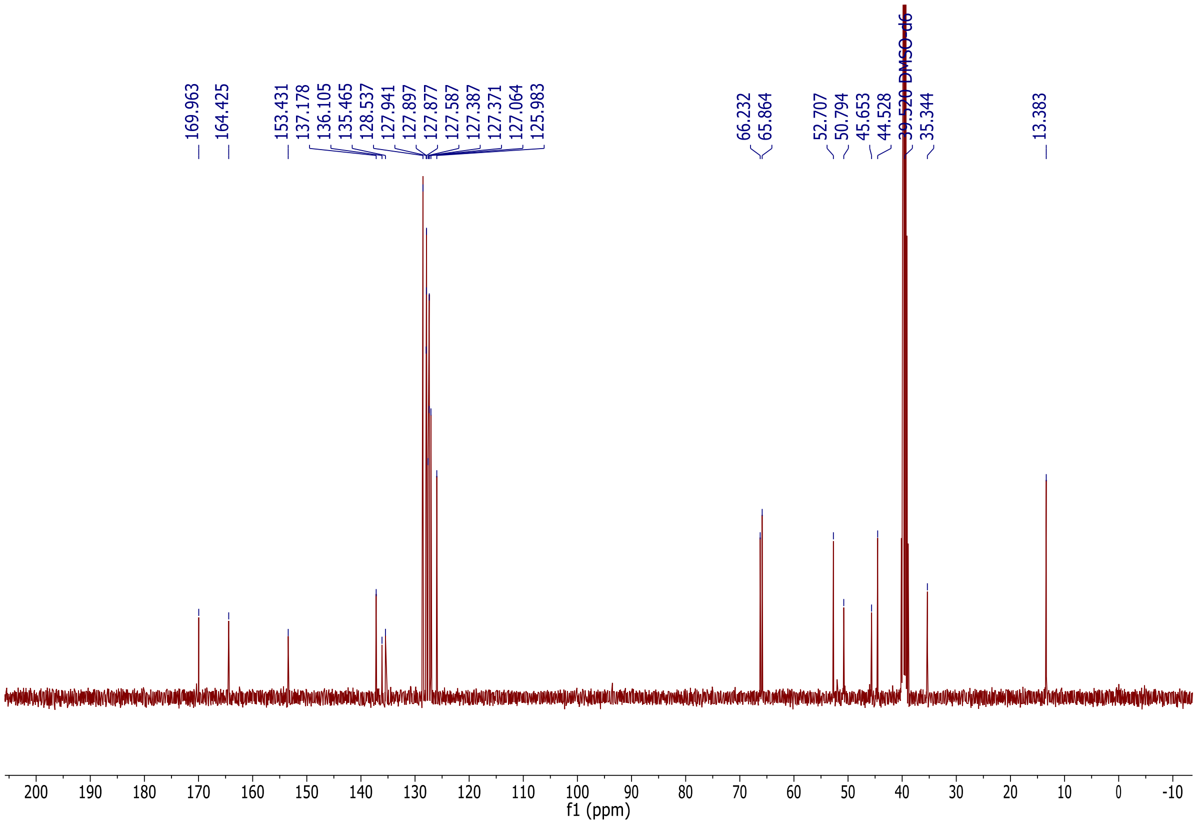

^{1}H-NMR (400 MHz, $CDCl_3$) and ^{13}C-NMR (75 MHz, $CDCl_3$) 39a

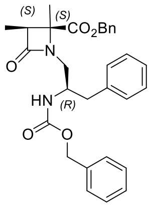

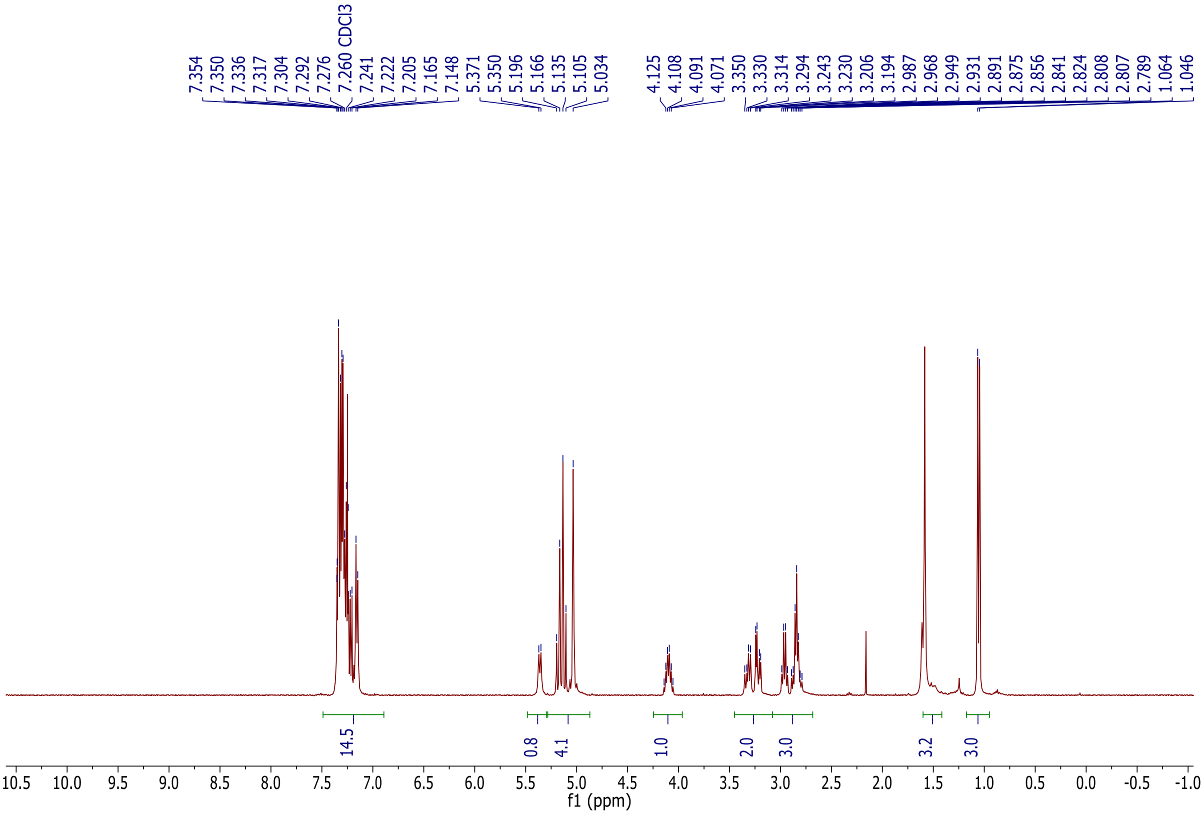

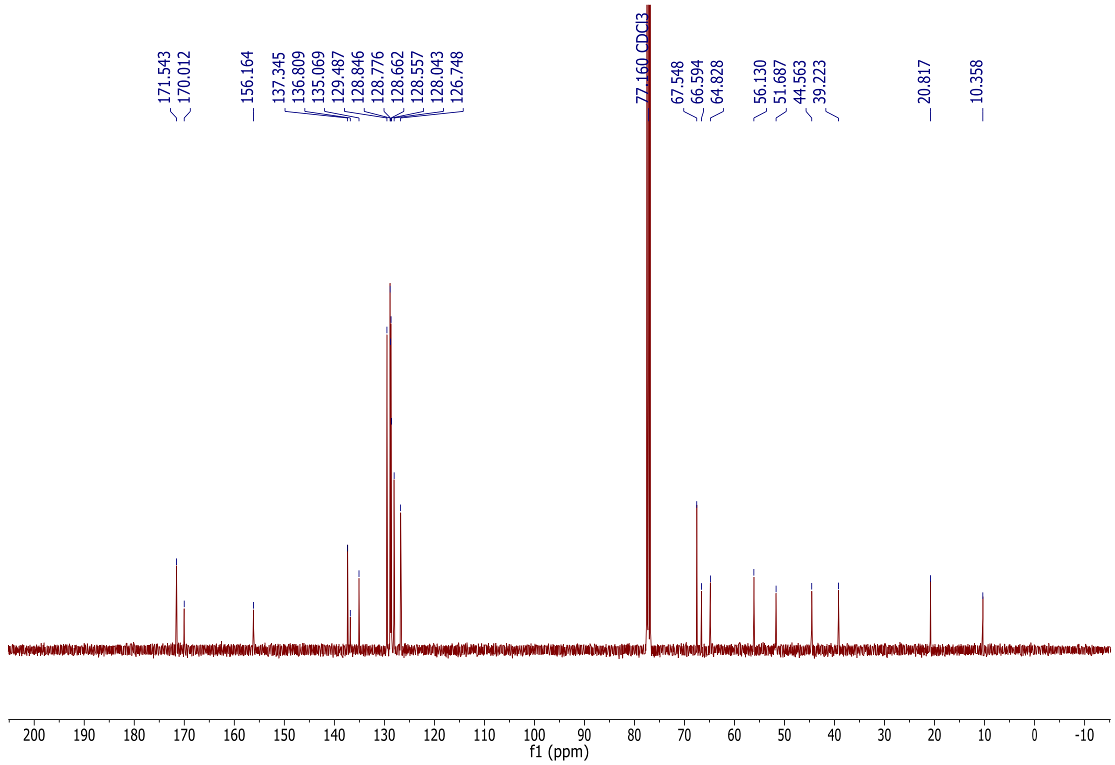

^{1}H-NMR (400 MHz, DMSO-d_6) and ^{13}C-NMR (100 MHz, DMSO-$d_{6,}$) 90 °C 40ab

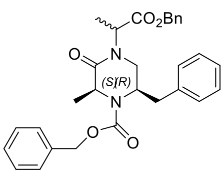

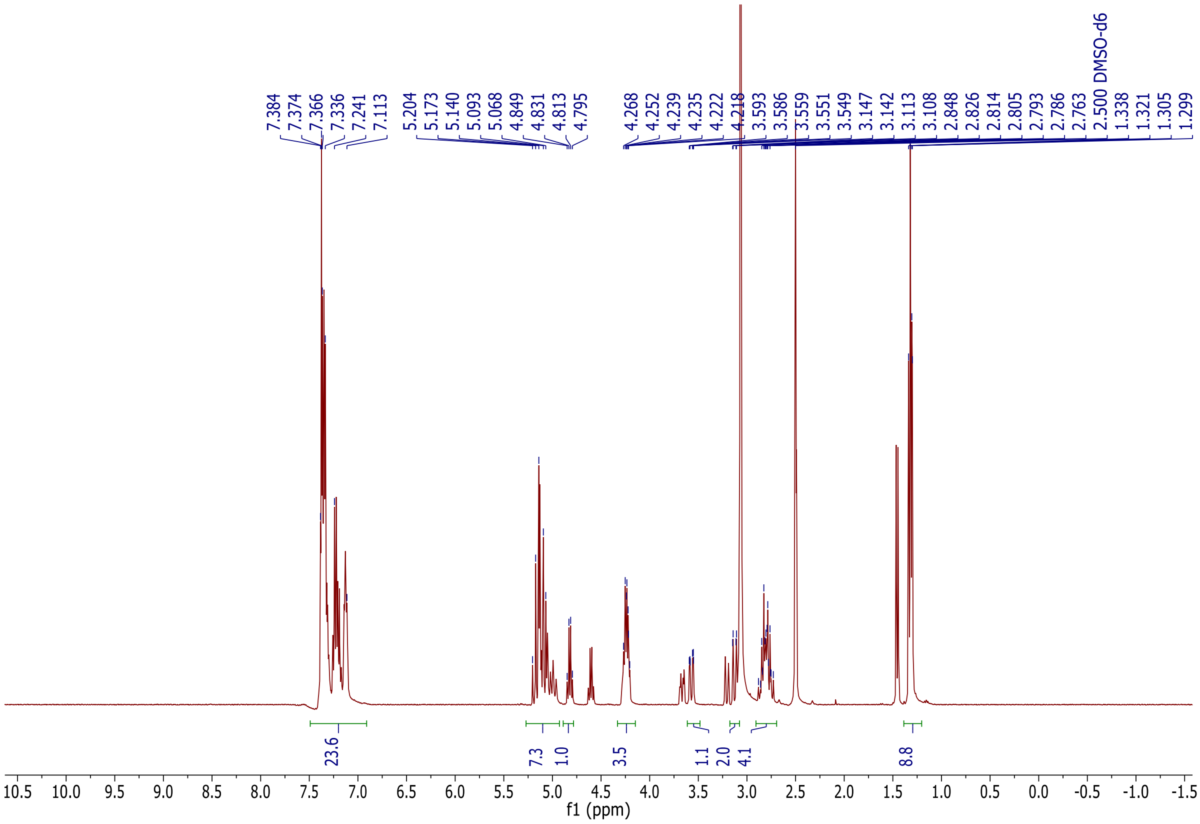

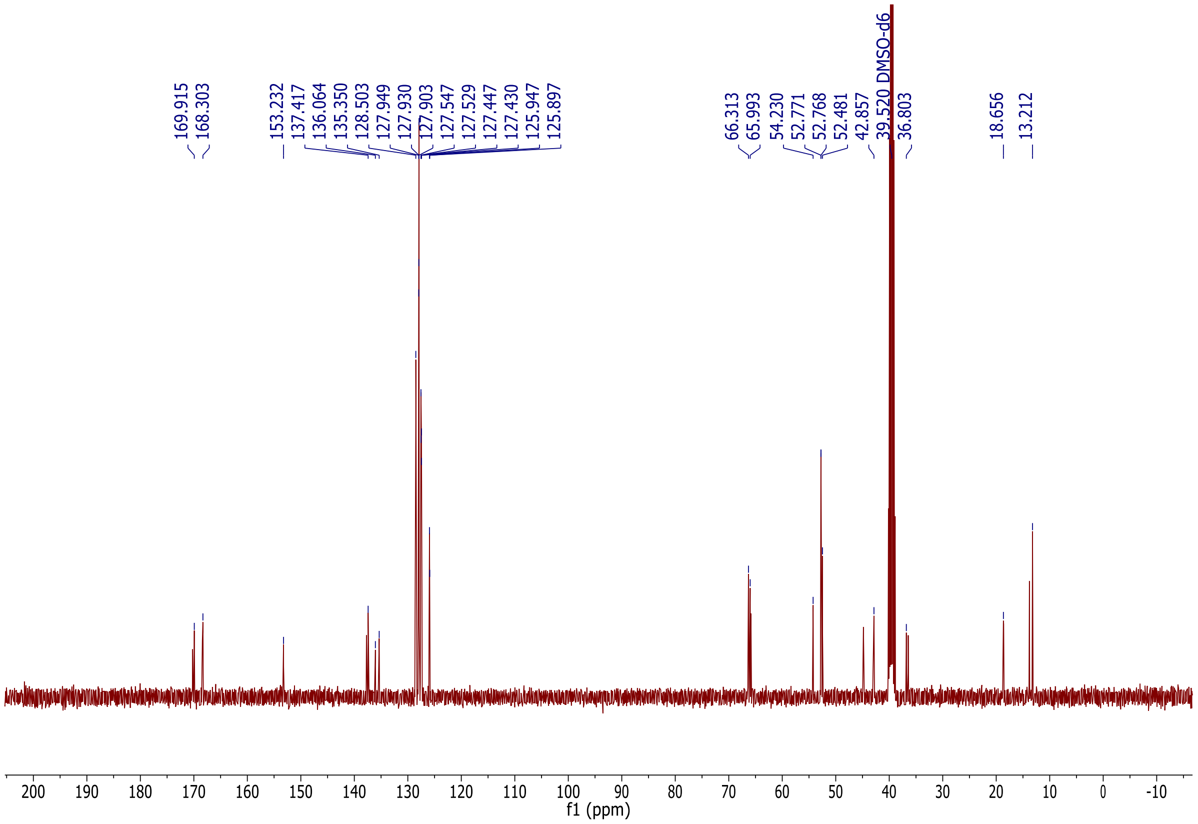

Supplement: Supplementary file 1 — Supplementary information 1 [file 41598_2020_70691_MOESM1_ESM.pdf]
