# Supplementary material for: Highly functionalized β-lactams and 2-ketopiperazines as TRPM8 antagonists with antiallodynic activity
Source: Sci Rep. 2020 Aug 25;10:14154. doi: 10.1038/s41598-020-70691-x (PMC7447632; doi:10.1038/s41598-020-70691-x)
Supplement: Supplementary file 2 — Supplementary information 2 [file 41598_2020_70691_MOESM2_ESM.pdf]

Highly functionalized β-lactams and 2-ketopiperazines as TRPM8 antagonists with antitumor and antiallodynic activity

M. Ángeles Bonache, Cristina Martín-Escura, Roberto de la Torre Martínez, Alicia Medina, Sara González-Rodríguez, Francesc Solloso, Carmen Cuevas, Ana María Roa, Gregorio Fernández-Ballester, Antonio Ferrer-Montiel, Asia Fernández-Carvajal, Rosario González-Muñiz

SUPPLEMENTARY INFORMATION

Synthetic procedures

General. All reactions were monitored by TLC silica gel plates Merck 60 F254. The chromatographic separations were carried out in column using Merck 60 (230-400) silica gel. ^{1}H NMR spectra were registered in a Varian INOVA-300 (300 MHz), Bruker 300 (300 MHz) and Varian INOVA-400 (400 MHz) spectrometers, employing TMS as internal standard. ^{13}C NMR spectra were registered in a Varian INOVA-300 (75 MHz) and in a Bruker 300 (75 MHz). Chemical shifts are expressed in ppm, the coupling constants are expressed in Hz, and the ^{13}C assignments were carried out by comparison with related analogues. Mass spectra (Electrospray, positive mode) were registered in a Hewlett-Packard 1100SD or in a HPLC-MS Waters spectrometer. Analytic HPLC was carried out in an Agilent Technologies 1120 Compact LC, using an Eclipse C18 (4.6 x 150 mm, 5µm) reversed-phase column. The mobile phase, which is referred as A:B, corresponds to a mixture of CH_3CN(A)/H_2O(0.05% TFA)(B). In all cases the flux was 1.5 mL/min and the detection was carried out by UV at 254 nm.

Preparation of synthetic intermediates

1. Synthesis of Ns-L-Xaa-OR^1 derivatives

Triethylamine (TEA) (17.15 mmol, 2.4 mL) was added to a solution of H-L-Xaa-OR^1 (17.15 mmol) in CH_2Cl_2 (115 mL). The mixture was stirred for 20 min. Then, TEA (22.25 mmol, 3.1 mL) and 2-nitrobenzenesulfonyl chloride (NsCl) (22.25 mmol, 4.9 g) were added to the reaction mixture at 0ºC. Stirring was continued at rt. When the reaction was completed (tlc), the solvent was removed and the residue was extracted with AcOEt and washed with citric acid (10%), $NaHCO_3$ (10%) and brine, successively. Finally, the organic phase was dried over dry Na_2SO_4, filtered, and concentrated. The residue was purified by flash chromatography on silica gel.

Ns-L-Phe-OBn (3)

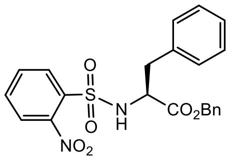

Syrup. Yield: 89%. Eluent: AcOEt:Hexane (3:1). HPLC: t_R=15.33 min (gradient of 5% to 100% of A, in 20 min). ^{1}H NMR (300 MHz, $CDCl_3$): δ 8.00-6.91 (m, 14H, Ar), 6.07 (d, 1H, *J*=9.0 Hz, NH), 4.92 (d, 1H, *J*=12.1 Hz, OCH_2), 4.87 (d, 1H, *J*=12.1 Hz, OCH_2), 4.52 (m, 1H, α-Phe), 3.13 (m, 2H, β-Phe). ^{13}C NMR (75 MMHz, $CDCl_3$): δ 171.5

(COO), 147.2, 136.6, 136.1, 135.1, 134.4, 132.8, 128.9, 128.6, 128.2, 127.7, 127.6, 127.1, 125.9, 124.2 (Ar), 66.4 (OCH_2), 58.7 (Cα), 35.9 (Cβ). MS $(ES)^+$: 441.21 $[M+H]^+$.

Ns-D-Phe-OMe (16)

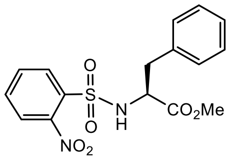

Solid. Mp: 83-85 ºC. Yied: 80%. Eluent: AcOEt:Hexane (3:1). HPLC: t_R=8.27 min (gradient of 15% to 95% of A, in 10 min). ^{1}H RMN (400 MHz, $CDCl_3$): δ 7.99 – 7.94 (m, 1H, Ar), 7.88 – 7.82 (m, 1H, Ar), 7.72 – 7.63 (m, 2H, Ar), 7.24 – 7.17 (m, 3H, Ar), 7.14 – 7.07 (m, 2H, Ar), 5.99 (d, *J* = 8.7 Hz, 1H, NH), 4.46 (ddd, *J* = 8.7, 6.9, 5.7 Hz, 1H, H_α), 3.16 (dd, *J* = 13.9, 5.7 Hz, 1H, H_β), 3.08 (dd, *J* = 13.9, 6.9 Hz, 1H, H_β). ^{13}C RMN (75 MHz, $CDCl_3$): δ 170.89 (CO), 147.57, 134.92, 134.31, 133.58, 133.00, 130.43, 129.38, 128.81, 127.57, 125.70 (Ar), 57.95 (C_α), 52.57(OCH_3), 39.35(C_β). MS $(ES)^+$: 382.08 $[M+H]^+$, 382.11 $[M+NH_4]^+$, 387.06 $[M+Na]^+$

Ns-L-Ala-OBn (33)

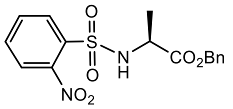

Syrup. Yield: 62% . Eluent: AcOEt:Hexano (2:1). HPLC: t_R=13.04 min (gradiente de 5% to 100% of A, en 20 min). ^{1}H RMN (400 MHz, $CDCl_3$): δ 8.00 (dd, 1H, *J*=7.7, 1.6 Hz, Ar), 7.81 (dd, 1H, *J*=7.7, 1.6 Hz, Ar), 7.34-7.31 (m, 3H, Ar), 7.20-7.17 (m, 2H, Ar), 6.18 (d, 1H, *J*=8.6 Hz, NH), 4.96 (d, 1H, *J*=12.2 Hz, OCH_2), 4.91 (d, 1H, *J*=12.1 Hz, OCH_2), 4.31 (m, 1H, α-H), 1.50 (d, 3H, *J*=7.2 Hz, CH_3). ^{13}C RMN (75 MHz, $CDCl_3$): 171.4 (COO), 147.6, 134.8, 134.1, 133.7, 132.9, 130.4, 128.7, 128.6, 128.3, 125.7 (C, Ar), 67.4 (OCH_2), 52.6 (Cα-H), 19.8(CH_3). MS $(ES)^+$: 365.09 $[M+H]^+$.

2. Synthesis of *N*-Ns-*N*-alkyl- Xaa derivatives

The corresponding alcohol derivative (3.8 mmol) and PPh_3 (3.8 mmol, 1 g) was added into a solution of Ns-L-Xaa-OR^1 (3.8 mmol) in dry THF (33 mL). The reaction mixture, under Ar atmosphere, was treated with diisopropyl azodicarboxylate (DIAD) (3.8 mmol, 0.75 mL). The reaction mixture was stirred overnight at rt. Then, the solvent was removed and the residue was purified by flash chromatography on silica gel, using the eluent indicated in each case.

***N*-[(2*S*-Benzyloxycarbonylamino-3-phenyl)prop-1-yl]-Ns-L-Phe-OBn (6)**

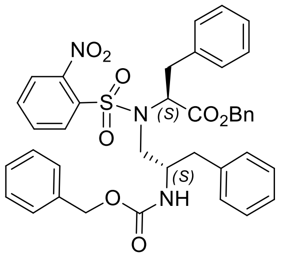

Syrup. Yield: 51% [from Ns-L-Phe-OBn (**3**) and Z-L-Phenylalaninol]. Eluent: AcOEt:Hexane (1:3). HPLC: t_R=18.52 min (gradient of 5% to 100% of A, in 20 min). ^{1}H NMR (400 MHz, $CDCl_3$): δ 7.79-7.02 (m, 24H, Ar), 5.42 (d, 1H, *J*=7.4 Hz, 2-NH), 5.10 (d, 1H, *J*=12.6 Hz, OCH_2), 5.00 (d, 1H, *J*=12.6 Hz, OCH_2), 4.84 (s, 2H, OCH_2), 4.82 (dd, 1H, *J*=8.4, 6.6 Hz, α-Phe), 4.14 (m, 1H, 2-H), 3.55 (dd, 1H, *J*=15.5, 10.1 Hz, 1-H), 3.43 (dd, 1H, *J*=15.5, 4.9 Hz, 1-H), 3.24 (dd, 1H, *J*=13.9, 8.5, β-Phe), 3.11 (dd, 1H, *J*=13.9, 6.6 Hz, β-Phe), 2.89 (dd, 1H, *J*=14.0, 6.0 Hz, 3-H), 2.81 (dd, 1H, *J*=14.0, 7.2 Hz, 3-H). ^{13}C NMR (75 MHz, $CDCl_3$): δ 170.0 (COO), 156.0 (CON), 147.8, 137.3, 136.1, 134.7, 133.6, 133.0, 131.9, 131.1, 129.5, 129.4, 128.6, 128.7, 128.66, 128.65, 128.63, 128.62, 128.5, 128.1, 127.9, 127.1, 126.8, 124.4 (Ar), 67.5, 66.5 (OCH_2), 62.0 (Cα-Phe), 52.2 (C2), 48.7 (C1), 39.6 (Cβ-Phe), 37.3 (C3). MS (ES)$^+$: 708.43 $[M+H]^+$.

***N*-[(2*R*-Benzyloxycarbonylamino-3-phenyl)prop-1-yl]-Ns-L-Phe-OBn (7)**

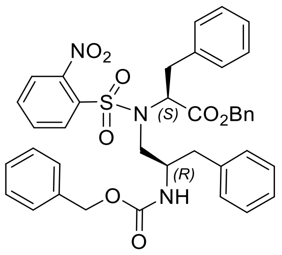

Syrup. Yield: 57% [from Ns-L-Phe-OBn (**3**) and Z-D-Phenylalaninol]. Eluent: AcOEt:Hexane (1:3). HPLC: t_R=18.64 min (gradient of 5% to 100% of A, in 20 min). ^{1}H NMR (400 MHz, $CDCl_3$): δ 7.69-6.88 (m, 24H, Ar), 5.22 (d, 1H, *J*=7.4 Hz, NH, Z), 5.01 (d, 1H, *J*=12.2 Hz, OCH_2), 5.00 (s, 2H, OCH_2), 4.97 (d, 1H, *J*=12.2 Hz, OCH_2), 4.83 (t, 1H, *J*=7.6 Hz, α-Phe), 4.06 (m, 1H, 2-H), 3.64 (dd, 1H, *J*=15.8, 9.3 Hz, 1-H), 3.45 (dd, 1H, *J*=15.8, 4.5 Hz, 1-H), 3.23 (dd, 1H, *J*=14.5, 7.6 Hz, β-Phe), 2.99 (dd, 1H, *J*=13.9, 6.9 Hz, 3-H), 2.79 (m, 2H, β-Phe, 3-H). ^{13}C NMR (75 MHz, $CDCl_3$): δ 170.1 (COO), 156.1 (CON), 148.0, 137.8, 135.8, 135.1, 133.7, 132.6, 131.6, 131.1, 129.4, 129.0, 128.75, 128.7, 128.6, 128.5, 128.05, 127.0, 126.8, 124.2 (Ar), 67.7, 66.5 (OCH_2), 61.0 (Cα-Phe), 52.4 (C2), 48.4 (C1), 39.2 (C3), 35.6 (Cβ-Phe). MS (ES)$^+$: 708.42 $[M+H]^+$.

N-[(2S-Benzyloxycarbonylamino-3-phenyl)prop-1-yl]-Ns-D-Phe-OMe (17)

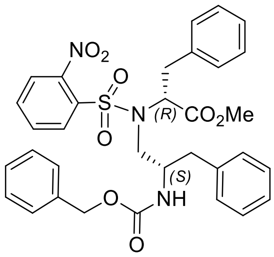

Syrup. Yield: 57% [from Ns-D-Phe-OMe (**16**) and Z-L-Phenylalaninol]. Eluent: AcOEt:Hexane (1:1). HPLC: t_R=17.11 min (gradient of 5% to 100% of A, in 20 min). ^{1}H NMR (400 MHz, $CDCl_3$): δ 7.65-6.82 (m, 19H, Ar), 5.35 (br s, 1H, NH, Z), 5.07 (d, 1H, *J*=12.6 Hz, OCH_2), 5.04 (d, 1H, *J*=12.6 Hz, OCH_2), 4.78 (t, 1H, *J*=6.9 Hz, α-Phe), 4.02 (m, 1H, 2-H), 3.61 (m, 1H, 1-H), 3.56 (s, 3H, OMe), 3.41 (m, 1H, 1-H), 3.22 (dd, 1H, *J*=14.7, 6.8 Hz, β-Phe), 3.05 (dd, 1H, *J*=13.7, 6.4 Hz, 3-H), 2.75 (dd, 1H, *J*=13.7, 7.7 Hz, 3-H), 2.70 (dd, 1H, *J*=14.7, 6.8 Hz, β-Phe). ^{13}C NMR (75 MHz, $CDCl_3$): δ 170.8 (COO), 156.1 (CON), 148.1, 137.8, 136.7, 135.7, 133.7, 132.6, 131.7, 130.9, 129.4, 128.8, 128.75, 128.7, 128.5, 128.0, 127.02, 126.8, 124.3 (Ar), 66.5 (OCH_2), 60.7 (Cα-Phe), 52.8 (OMe), 52.3 (C2), 48.2 (C1), 39.3 (C3), 35.2 (Cβ-Phe). MS $(ES)^+$: 632.40 $[M+H]^+$.

N-[(2R-Benzyloxycarbonylamino-3-phenyl)prop-1-yl]-Ns-L-Ala-OBn (34)

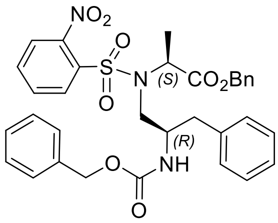

Syrup. Yield: 57% [from Ns-L-Ala-OBn (**33**) and Z-D-Phenylalaninol]. Eluent: AcOEt:Hexane (1:3). HPLC: t_R=16.91 min (gradient of 5% to 100% de A, in 20 min). ^{1}H NMR (400 MHz, $CDCl_3$): δ 7.76 (d, 1H, *J*=7.9 Hz, Ar), 7.58-7.14 (m, 13H, Ar), 5.15 (d, 1H, *J*=7.4 Hz NH, Z), 5.00 (m, 4H, OCH_2), 4.69 (q, 1H, *J*=7.3 Hz, α-Ala), 3.99 (m, 2H, 2-H), 3.46 (m, 2H, 1-H), 2.97 (dd, 1H, *J*=13.8, 6.3 Hz, 3-H), 2.89 (dd, 1H, *J*=13.8, 7.4 Hz, 3-H), 1.36 (d, 1H, *J*=7.3 Hz, CH_3). ^{13}C NMR (75 MHz, $CDCl_3$): δ 171.1 (COO), 156.0 (CON), 148.0, 137.7, 135.2, 133.7, 131.7, 131.2, 129.4, 129.3, 128.7, 128.6, 128.5, 128.45, 128.4, 128.3, 128.1, 128.0, 126.7, 124.2 (Ar), 67.5, 66.6 (OCH_2), 56.4 (Cα-Phe), 52.7 (C2), 48.2 (C1), 38.7 (C3), 16.01 (CH_3). MS $(ES)^+$: 632.33 $[M+H]^+$.

3. Removal of the Ns group

K_2CO_3 (3.3 mmol, 0.455 g) was added to a solution of the corresponding N-Ns-N-alkyl Xaa derivative (1.1 mmol) in CH_3CN (20 mL). Thiophenol (2.2 mmol, 0.22 mL) was added to the mixture and the reaction was stirred overnight. Then, the solvent was removed and the residue was extracted with AcOEt and washed with H_2O and a saturated solution of NaCl, successively. Finally, the organic phase was dried over dry Na_2SO_4, filtered, and concentrated. The residue was purified by flash chromatography on silica gel, using the eluent indicated in each case.

***N*-[(2*S*-Benzyloxycarbonylamino-3-phenyl)prop-1-yl]-L-Phe-OBn (8)**

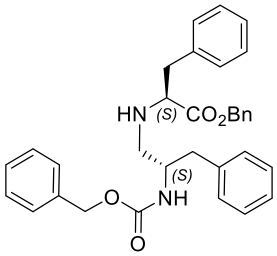

Syrup. Yield: 99% (from **6**). Eluent: AcOEt:Hexane (1:1). HPLC: t_R=15.41 min (gradient of 5% to 100% of A, in 20 min). ^{1}H NMR (400 MHz, $CDCl_3$): δ 7.39-7.00 (m, 20H, Ar), 5.09 (s, 4H, OCH_2), 5.08 (m, 1H, NH, Z), 3.87 (m, 1H, 2-H), 3.49 (dd, 1H, *J*=7.9, 6.0 Hz, α-Phe), 2.99 (dd, 1H, *J*=13.5, 6.0 Hz, 3-H), 2.86 (dd, 1H, *J*=13.5, 7.9 Hz, 3-H), 2.79 (m, 1H, β-Phe), 2.70 (m, 2H, β-Phe, 1-H), 2.40 (dd, 1H, *J*=12.3, 5.6 Hz, 1-H), 1.50 (br s, 1H, NH). ^{13}C NMR (75 MHz, $CDCl_3$): δ 173.7 (COO), 156.2 (CON), 137.7, 137.0, 136.7, 135.5, 129.4, 128.7, 128.6, 128.55, 128.5, 128.1, 127.0, 126.5 (Ar), 66.9, 66.7 (OCH_2), 63.1 (Cα-Phe), 52.2 (C2), 49.8 (C1), 39.2 (C3), 37.5 (Cβ-Phe). MS $(ES)^+$: 523.28 $[M+H]^+$.

***N*-[(2*R*-Benzyloxycarbonylamino-3-phenyl)prop-1-yl]-L-Phe-OBn (9)**

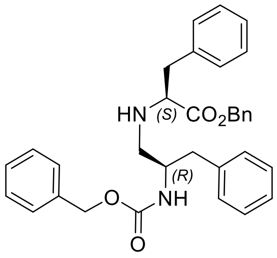

Syrup. Yield: 93% (from **7**). Eluent: AcOEt:Hexane (1:3). HPLC: t_R=15.53 min (gradient of 5% to 100% de A, in 20 min). ^{1}H NMR (400 MHz, $CDCl_3$): δ 7.37-7.09 (m, 20H, Ar), 5.06 (s, 2H, OCH_2), 5.04 (s, 2H, OCH_2), 4.99 (br s, 1H, 2-NH), 3.87 (m, 1H, 2-H), 3.45 (t, 1H, *J*=6.9 Hz, α-Phe), 2.92 (dd, 1H, *J*=13.5, 6.6 Hz, 3-H), 2.85 (m, 2H, 3-H, β-Phe), 2.64 (m, 2H, 1-H, β-Phe), 2.40 (dd, 1H, *J*=12.3, 4.7 Hz, 1-H), 1.59 (br s, 1H,

α-NH). ^{13}C NMR (75 MHz, $CDCl_3$): δ 174.5 (COO), 156.0 (CON), 137.8, 137.3, 136.8, 135.6, 129.5, 129.4, 128.7, 128.6, 128.55, 128.5, 128.1, 126.8, 126.5 (Ar), 66.7, 66.65 (OCH_2), 63.4 (Cα-Phe), 52.1 (C2), 49.8 (C1), 40.0 (C3), 39.0 (Cβ-Phe). MS (ES)$^+$: 523.05 $[M+H]^+$.

***N*-[(2*S*-Benzyloxycarbonylamino-3-phenyl)prop-1-yl]-D-Phe-OMe (18)**

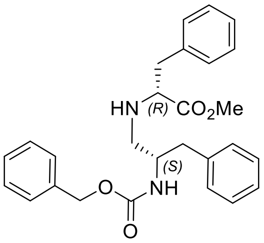

Syrup. Yield: 99% (from **17**). Eluent: AcOEt:Hexane (1:1). HPLC: t_R=10.17 min (gradient of 5% to 100% of A, in 20 min). ^{1}H NMR (400 MHz, $CDCl_3$): δ 7.37-7.10 (m, 15H, Ar), 5.07 (s, 2H, OCH_2), 3.87 (m, 1H, 2-H), 3.62 (s, 3H, OMe), 3.41 (dd, 1H, *J*=7.6, 6.2 Hz, α-Phe), 2.94 (dd, 1H, *J*=13.5, 6.1 Hz, 3-H), 2.85 (m, 1H, 3-H), 2.68 (m, 1H, 1-H), 2.64 (m, 2H, β-Phe), 2.40 (dd, 1H, *J*=12.2, 4.8 Hz, 1-H), 1.60 (br s, 1H, α-NH). ^{13}C NMR (75 MHz, $CDCl_3$): δ 175.1 (COO), 156.0 (CON), 137.8, 137.4, 129.5, 129.4, 129.3, 128.6, 128.5, 128.4, 128.1, 126.8, 126.5 (Ar), 66.2 (OCH_2), 63.3 (Cα-Phe), 52.0 (C2), 51.8 (OMe), 49.7 (C1), 39.9 (C3), 38.9 (Cβ-Phe). MS (ES)$^+$: 447.20 $[M+H]^+$.

***N*-[(2*R*-Benzyloxycarbonylamino-3-phenyl)prop-1-yl]-L-Ala-OBn (35)**

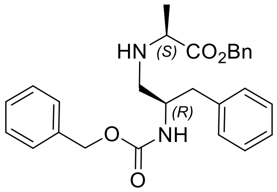

Syrup. Yield: 90% (from **34**). Eluent: AcOEt:Hexane (1:3). HPLC: t_R=10.25 min (gradient of 5% to 100% of A, in 20 min). ^{1}H NMR (400 MHz, $CDCl_3$): δ 7.36-7.16 (m, 15H, Ar), 5.15 (d, 1H, *J*=12.3 Hz, OCH_2), 5.11 (m, 3H, NH, Z, OCH_2), 5.10 (d, 1H, *J*=12.3 Hz, OCH_2), 3.92 (m, 1H, 2-H), 3.30 (q, 1H, *J*=7.0 Hz, α-Ala), 2.89 (dd, 1H, *J*=13.9, 5.6 Hz, 3-H), 2.70 (dd, 1H, *J*=13.9, 7.8 Hz, 3-H), 2.82 (dd, 1H, *J*=12.0, 6.1 Hz, 1-H), 2.70 (dd, 1H, *J*=12.0, 4.5 Hz, 1-H), 1.27 (d, 3H, *J*=6.9 Hz, CH_3 Ala). ^{13}C NMR (75 MHz, $CDCl_3$): δ 175.6 (COO), 156.1 (CON), 137.8, 136.7, 135.8, 129.5, 128.9, 128.7, 128.65, 128.6, 128.5, 128.3, 128.2, 126.6 (Ar), 66.7, 66.6 (2C, OCH_2), 57.0 (Cα-Ala), 52.2 (C2), 49.9 (C1), 39.2 (C3), 19.4 (CH_3 Ala). MS (ES)$^+$: 447.36 $[M+H]^+$.

4. Synthesis of *N*-alkyl-*N*-chloroacethyl-Xaa derivatives

Propylene oxyde (34.3 mmol, 2.4 mL) was added to a solution of the corresponding secondary amine (2.28 mmol) in dry THF (9 mL). Chloroacethyl chloride (2.75 mmol, 0.27 g) was added to the reaction mixture at 0ºC. The reaction mixture was stirred overnight. The solvent was removed and the residue was purified by flash chromatography on silica gel, using the eluent indicated in each case.

***N*-Chloroacethyl-*N*-[(2*S*-benzyloxycarbonylamino-3-phenyl)prop-1-yl]-L-Phe-OBn (10)**

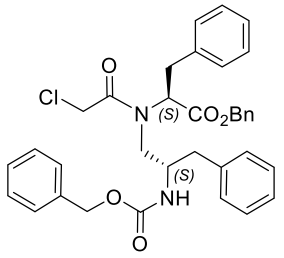

Syrup. Yield: 86% (from **8**). Eluent: AcOEt:Hexane (1:2). HPLC: t_R= 22.50 min (gradient of 30% to 95% d A, en 30 min). Mixture of rotamers M,m =16:1. ^{1}H NMR (400 MHz, DMSO-d_6, major rotamer): δ 7.33-6.97 (m, 20H, Ar), 5.10 (d, 1H, *J*=12.6 Hz, OCH_2), 5.08 (br s, 1H, 2-NH), 5.02 (d, 1H, *J*=12.6 Hz, OCH_2), 4.87 (d, 1H, *J*=12.6 Hz, OCH_2), 4.78 (d, 1H, *J*=12.6 Hz, OCH_2), 4.50 (d, 1H, *J*=13.2 Hz, CH_2Cl), 4.33 (d, 1H, *J*=13.2 Hz, CH_2Cl), 4.30 (dd, 1H, J = 9.4, 5.3 Hz, α-Phe), 3.72 (m, 1H, 2-H), 3.27 (dd, 1H, *J*=13.8, 5.2 Hz, β-Phe), 3.19 (dd, 1H, *J*=15.5, 6.4 Hz, 1-H), 3.10 (dd, 1H, *J*=13.8, 9.4 Hz, β-Phe), 2.84 (dd, 1H, *J*=13.5, 4.7 Hz, 3-H), 2.46 (m, 1H, 1-H), 2.40 (dd, 1H, *J*=13.4, 6.8 Hz, 3-H). ^{13}C NMR (75 MHz, DMSO-d_6, major rotamer): δ 169.4 (COO), 165.5 (CON), 155.7 (OCON), 138.2, 137.6, 136.9, 136.0, 129.3, 128.9, 128.5, 128.4, 128.3, 128.1, 127.8, 127.7, 127.6, 127.45, 126.4, 126.0 (Ar), 66.1, 65.2 (OCH_2), 63.3 (Cα-Phe), 53.5 (C1), 52.8 (C2), 41.3 (CH_2Cl), 37.4 (C3), 34.0 (Cβ-Phe). MS (ES)$^+$: 599.24 $[M+H]^+$.

***N*-Chloroacethyl-*N*-[(2*R*-benzyloxycarbonylamino-3-phenyl)prop-1-yl]-L-Phe-OBn (11)**

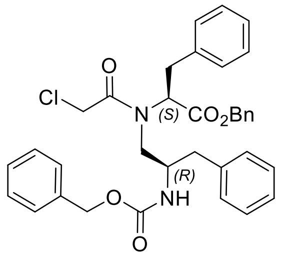

Syrup. Yield: 83% (from **9**). Eluent: AcOEt:Hexane (1:2). HPLC: t_R=22.71 min (gradient of 5% to 100% de A, in 20 min). Mixture of rotamers M,m =5:1. ^{1}H NMR (400 MHz, DMSO-d_6, major rotamer): δ 7.32-7.06 (m, 20H, Ar), 5.11 (br s, 1H, 2-NH), 5.11 (d, 1H, *J*=12.6 Hz, OCH_2), 5.07 (d, 1H, *J*=12.6 Hz, OCH_2), 4.88 (s, 2H, OCH_2), 4.37 (m, 1H, α-Phe), 4.28 (s, 2H, CH_2Cl), 3.85 (m, 1H, 2-H), 3.39 (m, 1H, β-Phe), 3.30 (m, 1H, 1-H), 3.07 (dd, 1H, *J*=13.8, 7.7 Hz, β-Phe), 2.80 (dd, 1H, *J*=13.7, 4.5 Hz, 3-H), 2.75 (m, 1H, 1-H), 2.54 (m, 1H, 3-H). ^{13}C NMR (75 MHz, DMSO-d_6, major rotamer): δ 169.5 (COO), 166.1 (CON), 155.6 (OCON), 138.4, 138.1, 137.1, 135.9, 129.4, 129.0, 128.3, 128.2, 128.0, 127.8, 127.6, 127.35, 126.3, 126.0 (Ar), 66.1, 64.9 (OCH_2), 62.3 (Cα-Phe), 53.5 (C1), 51.7 (C2), 41.5 (CH_2Cl), 37.3 (C3), 34.5 (Cβ-Phe). MS (ES)$^+$: 599.15 [M+H]$^+$.

***N*-Chloroacethyl-*N*-[(2*S*-benzyloxicarbonylamino-3-fenil)prop-1-yl]-D-Phe-OMe (19)**

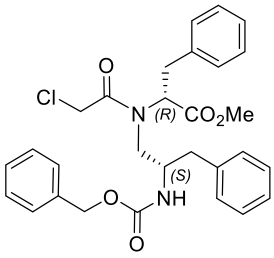

Syrup. Yield: 83% (from **18**). Eluent: AcOEt:Hexane (1:1). HPLC: t_R= 17.29 min (gradient of 30% to 95% of A, in 30 min). Mixture of rotamers M,m =1:0.10. ^{1}H NMR (400 MHz, DMSO-d_6, major rotamer): δ 7.32-7.15 (m, 15H, Ar), 4.91 (s, 2H, OCH_2), 4.88 (br s, 1H, NH, Z), 4.29 (m, 1H, α-Phe), 4.27 (d, 1H, *J*=13.2 Hz, CH_2Cl), 4.23 (d, 1H, *J*=13.2 Hz, CH_2Cl), 3.87 (m, 1H, 2-H), 3.55 (s, 3H, OMe), 3.40 (m, 1H, β-Phe), 3.30 (dd, 1H, *J*=14.5, 7.0 Hz, 1-H), 3.04 (dd, 1H, *J*=14.9, 8.7 Hz, β-Phe), 2.81 (dd, 1H, *J*=13.4, 4.3 Hz, 3-H), 2.66 (dd, 1H, *J*=14.5, 7.9 Hz, 1-H), 2.54 (m, 1H, 3-H). ^{13}C NMR (75 MHz, DMSO-d_6, major rotamer): δ 170.1 (COO), 166.0 (CON), 155.6 (CON), 138.4, 138.0, 137.1, 129.4, 129.0, 128.3, 128.2, 128.1, 127.6, 127.3, 126.4, 126.1 (Ar), 65.0 (OCH_2), 62.5 (Cα-Phe), 53.3 (C1), 52.1 (C2), 51.9 (OMe), 41.6 (CH_2Cl), 37.6 (C3), 34.3 (Cβ-Phe). MS (ES)$^+$: 523.35 [M+H]$^+$.

***N*-Chloroacethyl-*N*-[(2*R*-benzyloxycarbonylamino-3-phenyl)prop-1-yl]-L-Ala-OBn (36)**

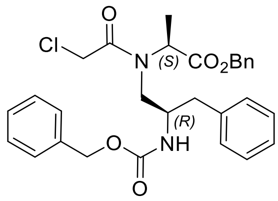

Syrup. Yield: 86% (from **35**). Eluent: AcOEt:Hexane (1:3). HPLC: t_R=15.50 min (gradient of 5% to 100% of A, in 20 min). Mixture of rotamers M,m =6:1. ^{1}H NMR (400 MHz, DMSO-d_6, major rotamer): δ 7.35- 7.12 (m, 15H, Ar), 5.14 (br s, 1H, 2-NH), 5.09 (d, 1H, *J*=12.2 Hz, OCH_2), 5.06 (d, 1H, *J*=12.2 Hz, OCH_2), 4.92 (d, 1H, *J*=12.9 Hz, OCH_2), 4.87 (d, 1H, *J*=12.9 Hz, OCH_2), 4.45 (d, 1H, *J*=13.4 Hz, CH_2Cl), 4.28 (d, 1H, *J*=13.4 Hz, CH_2Cl), 4.20 (q, 1H, *J*=6.8 Hz, α-Ala), 3.95 (m, 1H, 2-H), 3.48 (dd, 1H, *J*=15.1, 9.2 Hz, 1-H), 3.40 (dd, 1H, *J*=15.1, 5.0 Hz, 1-H), 2.82 (dd, 1H, *J*=13.5, 4.2 Hz, 3-H), 2.58 (dd, 1H, *J*=13.5, 10.3 Hz, 3-H), 1.38 (d, 3H, *J*=6.8 Hz, CH_3). ^{13}C NMR (75 MHz, DMSO-d_6, major rotamer): δ 171.0 (COO), 166.4 (CON), 156.3 (OCON), 138.6, 137.5, 136.3, 129.5, 128.75, 128.7, 128.5, 128.3, 128.0, 127.9, 127.4, 126.6 (Ar), 66.3, 65.3 (OCH_2), 57.0 (Cα-Ala), 53.5 (C1), 51.8 (C2), 42.3 (CH_2Cl), 38.1 (C3), 14.5 (CH_3). MS $(ES)^+$: 523.28 $[M+H]^+$.

5. Synthesis of *N*-alkyl-*N*-chloropropionyl-Xaa derivatives

PPh_3 (0.513 g, 1.96 mmol) was added to a solution of (*R*)- or (*S*)-2-chloropropionic acid (0.126 mL, 1.47 mmol) and Cl_3CCN (0.19 mL, 1.96 mmol) in THF (8 mL) at 0ºC, and the reaction mixture was stirred for 30 min. Then, a solution of the corresponding secondary amine (0.98 mmol) and propylene oxyde (1 mL, 14.7 mmol) in THF (2 mL) was added dropwise to the reaction mixture. After stirring for 48 h, the solvent was evaporated, the resulting residue was dissolved in Et_2O, filtered over celite and concentrated under vacuum. The residue was purified by flash chromatography on silica gel, using the eluent indicated in each case.

N-(2’*S*-Chloropropanoyl)-*N*-[(2*S*-benzyloxycarbonylamino-3-phenyl)prop-1-yl]-L-Phe-OBn (22)

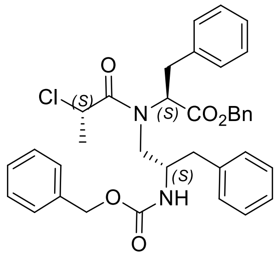

Syrup. Yield: 51% (from **8**). Eluent: AcOEt:Hexane (1:4). HPLC: t_R=16.54 min (gradient of 5% to 100% de A, in 20 min). $[\alpha]_D$ = -36.14 (c 1, $CHCl_3$). Mixture of

rotamers M,m =10:1. ^{1}H NMR (400 MHz, DMSO-d_6, major rotamer): δ 7.33-6.96 (m, 20H, Ar), 5.10 (d, 1H, *J*=12.6 Hz, OCH_2), 5.04 (d, 1H, *J*=12.6 Hz, OCH_2), 5.01 (q, 1H, *J*=6.3 Hz, 1'-H), 4.87 (d, 1H, *J*=12.6 Hz, OCH_2), 4.81 (d, 1H, *J*=12.6 Hz, OCH_2), 4.39 (dd, 1H, *J*=9.0, 5.8 Hz, α-Phe), 3.74 (m, 1H, 2-H), 3.33 (m, 1H, β-Phe), 3.13 (m, 2H, β-Phe, 1-H), 2.90 (dd, 1H, *J*=13.5, 4.5 Hz, 3-H), 2.64 (m, 1H, 1-H), 2.50 (m, 1H, 3-H), 1.51 (d, 3H, *J*=6.4 Hz, 2'-H). ^{13}C NMR (75 MHz, DMSO-d_6, major rotamer): δ 169.4 (COO, CON), 155.7 (OCON), 138.3, 137.1, 136.8, 135.7, 129.2, 128.9, 128.3, 128.2, 128.1, 128.0, 128.0, 127.9, 127.7, 127.45, 126.4, 126.1 (Ar), 66.2, 65.2 (OCH_2), 63.6 (Cα-Phe), 53.6 (C1), 52.9 (C2), 50.3 (CHCl), 36.8 (C3), 34.2 (Cβ-Phe), 21.7 (C2'). MS $(ES)^+$: 613.29 $[M+H]^+$.

N-(2'*R*-Chloropropanoyl)-*N*-[(2*S*-benzyloxycarbonylamino-3-phenyl)prop-1-yl]-L-Phe-OBn (23)

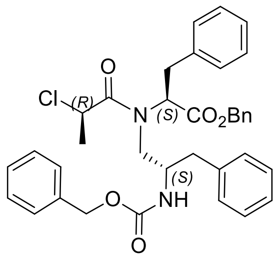

Syrup. Yield: 50% (from **8**). Eluent: AcOEt:Hexane (1:4). HPLC: t_R=17.39 min (gradient of 30% a 95% de A, in 20 min). $[\alpha]_D$ = -64.09 (c 1, $CHCl_3$). Mixture of rotamers M,m =5:1. ^{1}H NMR (400 MHz, DMSO-d_6, major rotamer): δ 7.29-6.94 (m, 20H, Ar), 5.18 (d, 1H, *J*=12.5 Hz, OCH_2), 5.07 (q, 1H, *J*=6.3 Hz, 1'-H), 4.91 (d, 1H, *J*=12.5 Hz, OCH_2), 4.84 (d, 1H, *J*=12.6 Hz, OCH_2), 4.74 (d, 1H, *J*=12.6 Hz, OCH_2), 4.21 (dd, 1H, *J*=10.1, 4.7 Hz, α-Phe), 3.66 (m, 1H, 2-H), 3.23 (m, 2H, β-Phe, 1-H), 3.10 (dd, 1H, *J*=13.6, 10.1 Hz, β-Phe), 2.75 (dd, 1H, *J*=13.5, 5.1 Hz, 3-H), 2.48 (m, 1H, 3-H), 2.22 (dd, 1H, *J*=15.5, 5.9 Hz,1-H), 1.42 (d, 3H, *J*=6.3 Hz, 2'-H). ^{13}C NMR (75 MHz, DMSO-d_6, major rotamer): δ 169.2 (COO), 168.7 (CON), 155.7 (OCON), 138.1, 137.4, 136.9, 136.1, 129.5, 128.9, 128.3, 128.2, 128.1, 127.9, 127.7, 127.4, 126.4, 126.1 (Ar), 65.9, 65.2 (OCH_2), 63.2 (Cα-Phe), 53.2 (C1), 53.0 (C2), 40.0 (C1'), 37.6 (C3), 33.9 (Cβ-Phe), 20.7 (C2'). MS $(ES)^+$: 613.36 $[M+H]^+$.

N-(2'*S*-chloropropanoyl)-*N*-[(2*R*-benzyloxycarbonylamino-3-phenyl)prop-1-yl]-L-Phe-OBn (27)

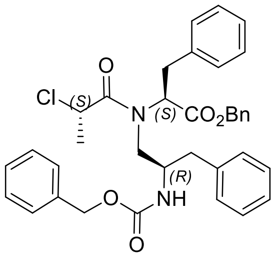

Syrup. Yield: 50% (from **9**). Eluent: AcOEt:Hexane (1:4). HPLC: t_R=16.65 min (gradient of 5% to 100% of A, in 20 min). $[\alpha]_D$ = -44.34 (c 1, $CHCl_3$). Mixture of rotamers M,m =10:1. ^{1}H NMR (400 MHz, DMSO-d_6, major rotamer): δ 7.31-7.09 (m, 20H, Ar), 5.07 (s, 2H, OCH_2), 4.97 (q, 1H, *J*=6.4 Hz, 1'-H), 4.90 (d, 1H, *J*=12.6 Hz, OCH_2), 4.86 (d, 1H, *J*=12.6 Hz, OCH_2), 4.40 (dd, 1H, *J*=7.7, 5.9 Hz, α-Phe), 3.93 (m, 1H, 2-H), 3.44 (m, 2H, β-Phe, 1-H), 3.13 (dd, 1H, *J*=15.2, 8.4 Hz, 1-H), 3.00 (dd, 1H, *J*=14.0, 5.9 Hz, β-Phe), 2.84 (dd, 1H, *J*=13.6, 4.2 Hz, 3-H), 2.56 (m, 1H, 3-H), 1.43 (d, 3H, *J*=6.3 Hz, 2'-H). ^{13}C NMR (75 MHz, DMSO-d_6, major rotamer): δ 169.7 (COO), 168.7 (CON), 155.7 (OCON), 138.6, 138.4, 137.1, 135.8, 129.4, 128.9, 128.3, 128.2, 128.1, 127.9, 127.8, 127.7, 127.35, 127.3, 126.2, 126.1 (Ar), 66.1, 65.0 (OCH_2), 62.2 (Cα-Phe), 53.5 (C1), 51.8 (C2), 49.7 (C1'), 37.5 (C3), 35.0 (Cβ-Phe), 21.3 (C2'). MS $(ES)^+$: 613.43 $[M+H]^+$.

N-(2'*R*-Chloropropanoyl)-*N*-[(2*R*-Benzyloxycarbonylamino-3-phenyl)prop-1-yl]-L-Phe-OBn (28)

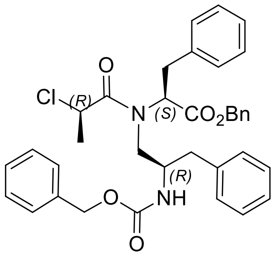

Syrup. Yield: 50% (from **9**). Eluent: AcOEt:Hexane +-(1:3). HPLC: t_R=17.23 min (gradient of 30% to 95% of A, in 20 min). $[\alpha]_D$ = -102.87 (c 1, $CHCl_3$). Mixture of rotamers M,m =10:1. ^{1}H NMR (400 MHz, DMSO-d_6, major rotamer): δ 7.32-7.01 (m, 20H, Ar), 5.21 (d, 1H, *J*=12.6 Hz, OCH_2), 4.97 (d, 1H, *J*=12.6 Hz, OCH_2), 4.88 (s, 2H, OCH_2), 4.54 (q, 1H, *J*=6.4 Hz, 1'-H), 4.35 (dd, 1H, *J*=9.5, 5.1 Hz, α-Phe), 3.75 (m, 1H, 2-H), 3.34 (m, 3H, β-Phe, 1-H), 3.14 (m, 1H, β-Phe), 2.66 (m, 1H, 3-H), 2.44 (dd, 1H, *J*=15.9, 6.4 Hz, 3-H), 1.36 (d, 3H, *J*=6.3 Hz, 2'-H). ^{13}C NMR (75 MHz, DMSO-d_6, major rotamer): δ 169.37 (COO), 168.34 (CON), 155.5 (OCON), 138.3, 137.6, 137.1, 135.9, 129.6, 129.0, 128.3, 128.2, 128.1, 128.0, 127.9, 127.7, 127.4, 126.5, 126.1 (Ar),

66.1, 65.1 (OCH_2), 62.6 (Cα-Phe), 52.9 (C1), 52.2 (C2), 49.5 (C1'), 37.1 (C3), 34.05 (Cβ-Phe), 20.7 (C2'). MS $(ES)^+$: 613.29 $[M+H]^+$.

***N*-(2'*S*-Chloropropanoyl)-*N*-[(2*R*-benzyloxycarbonylamino-3-phenyl)prop-1-yl]- L-Ala-OBn (37)**

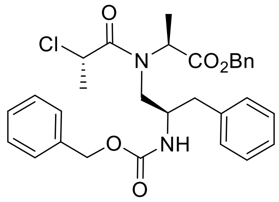

Syrup. Yield: 55% (from **35**). Eluent: AcOEt:Hexane (1:4). HPLC: t_R=15.50 min (gradient of 5% to 100% ofA, in 20 min). Two rotamers M,m =10:1. ^{1}H NMR (400 MHz, $CDCl_3$): δ 7.35- 7.10 (m, 15H, Ar), 5.10 (d, 1H, *J*=12.8 Hz, OCH_2), 5.03 (d, 1H, *J*=12.8 Hz, OCH_2), 5.06 (br s, 1H, 2-NH), 5.01 (q, 1H, J = 6.3 Hz, 1'-H), 4.90 (d, 1H, *J*=12.9 Hz, OCH_2), 4.85 (d, 1H, *J*=12.9 Hz, OCH_2), 4.20 (q, 1H, *J*=6.7 Hz, α-Ala), 3.97 (m, 1H, 2-H), 3.64 (dd, 1H, *J*=14.9, 10.5 Hz, 1-H), 3.40 (m, 1H, 1-H), 3.36 (s, 3H, OMe), 2.83 (dd, 1H, *J*=13.6, 4.0 Hz, 3-H), 2.58 (dd, 1H, *J*=13.6, 10.5 Hz, 3-H), 1.40 (d, 3H, *J*=6.9 Hz, 2'-H), 1.37 (d, 3H, *J*=6.3 Hz, CH_3). ^{13}C NMR (75 MHz, $CDCl_3$): δ 170.6 (COO), 168.1 (CON), 155.8 (OCON), 138.3, 137.2, 136.0, 129.1, 128.2, 128.1, 127.8, 127.6, 127.2, 126.1 (Ar), 65.8, 64.8 (OCH_2), 56.8 (Cα-Ala), 53.8 (C1), 51.2 (C2), 49.5 (C1'), 37.9 (C3), 20.8 (CH_3) 14.5 (C2'). MS $(ES)^+$: 537.05 $[M+H]^+$.

6. Characterization of final compounds.

4*R*,*S*-Benzyl-4-benzyloxycarbonyl-1-[(2'*S*-benzyloxycarbonylamino-3'-phenyl)prop-1'-yl]-2-oxoazetidine (12ab).

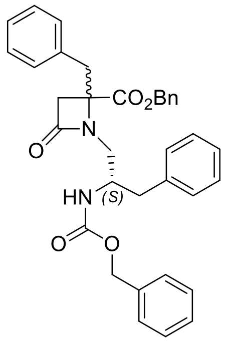

Syrup. Yield: 11% (from 10, B: Cs_2CO_3). Eluent: EtOAc:Hexane (1:3). HPLC: t_R = 20.74 min (gradient of 30% to 95% of A, in 30 min). Isomers ratio M(4*S*,2'*S*):m(4*R*,2'*S*) = 83:17. ^{1}H NMR (500 MHz, $CDCl_3$, major isomer): δ 7.34-6.98

(m, 20H, Ar), 5.48 (d, 1H, *J*=8.5 Hz, 2-NH), 5.15 (d, 1H, *J*=12.0 Hz, OCH_2), 5.09 (d, 1H, *J*=12.0 Hz, OCH_2), 5.06 (d, 1H, *J*=12.5 Hz, OCH_2), 5.02 (d, 1H, *J*=12.5 Hz, OCH_2), 4.15 (m, 1H, 2'-H), 3.37 (dd, 1H, *J*=14.5, 8.1 Hz, 1'-H), 3.27 (d, 1H, *J*=14.0 Hz, 4-CH_2), 3.22 (d, 1H, *J*=14.9 Hz, 3-H), 3.18 (dd, 1H, *J*=14.5, 4.3 Hz, 1'-H), 2.98 (d, 1H, *J*=14.0 Hz, 4-CH_2), 2.90 (d, 1H, *J*=14.9 Hz, 3-H), 2.85 (dd, 1H, *J*=14.1, 7.6 Hz, 3'-H), 2.76 (dd, 1H, *J*=14.1, 6.8 Hz, 3'-H). ^{13}C NMR (125 MHz, $CDCl_3$, major isomer): δ 170.8 (COO), 167.4 (C2), 156.1 (OCON), 137.4, 134.9, 134.2, 129.7, 129.4, 128.9, 128.8, 128.7, 128.6, 128.1, 128.0, 127.6, 126.8 (Ar), 67.9, 67.8 (OCH_2), 63.2 (C4), 51.6 (C2'), 45.4 (C3), 45.2 (C1'), 39.7 (4-CH_2), 39.1 (C3'). MS (ES)$^+$: 563.46 [M+H]$^+$. Exact Mass calculated for $C_{35}H_{34}N_2O_5$: 562.24677; found: 562.24804.

4*R,S*-Benzyl-4-benzyloxycarbonyl-1-[(2'*R*-benzyloxycarbonylamino-3'-phenyl)prop-1'-yl]-2-oxoazetidine (14ab).

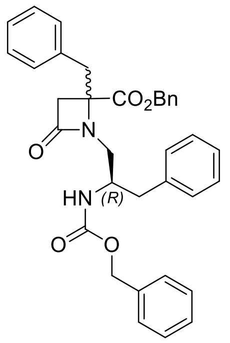

Syrup. Yield: 11% (from 11, B: Cs_2CO_3). Eluent: EtOAc:Hexane (1:3). HPLC: t_R = 20.70 min (gradient of 30% to 95% of A, in 30 min). Isomers ratio M(4*S*,2'*R*):m(4*R*,2'*R*) = 88:12. ^{1}H NMR (400 MHz, $CDCl_3$, major isomer): δ 7.34-6.99 (m, 20H, Ar), 5.54 (d, 1H, *J*=8.5 Hz, 2-NH), 5.14 (d, 1H, *J*=12.0 Hz, OCH_2), 5.06 (s, 2H, OCH_2), 5.04 (d, 1H, *J*=12.0 Hz, OCH_2), 4.16 (m, 1H, 2'-H), 3.29 (d, 1H, *J*=14.0 Hz, 4-CH_2), 3.22 (m, 3H, 3-H, 1'-H), 3.15 (d, 1H, *J*=14.0 Hz, 4-CH_2), 2.89 (d, 1H, *J*=14.9 Hz, 3-H), 2.78 (dd, 1H, *J*=14.0, 5.5 Hz, 3'-H), 2.72 (dd, 1H, *J*=14.0, 6.8 Hz, 3'-H). ^{13}C NMR (75 MHz, $CDCl_3$, major isomer): δ 170.9 (COO), 167.1 (C2), 156.2 (OCON), 137.2, 134.8, 134.5, 129.6, 129.4, 128.9, 128.85, 128.8, 128.6, 128.5, 128.1, 127.5, 126.7 (Ar), 67.8, 66.6 (OCH_2), 63.3 (C4), 51.5 (C2'), 45.8 (C3), 45.7 (C1'), 40.3 (4-CH_2), 39.1 (C3'). MS (ES)$^+$: 563.46 [M+H]$^+$. Exact Mass calculated for $C_{35}H_{34}N_2O_5$: 562.24677; found: 562.24834.

4*R*-Benzyl-4-benzyloxycarbonyl-3*R,S*-methyl-1-[(2'*S*-benzyloxycarbonylamino-3'-phenyl]prop-1'-yl]-2-oxoazetidine (25ab).

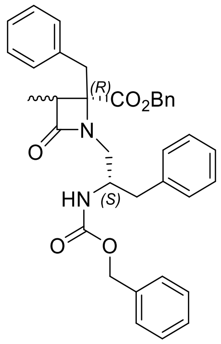

Syrup. Yield: 10% (from 23, B: BTPP). Eluent: EtOAc:Hexane (1:3). HPLC: tR = 16.36 min (gradient of 30% to 95% of A, in 20 min). Isomers ratio M(3*R*,4*R,2'S*):m(3*S*,4*R,2'S*)= 85:15. ^{1}H NMR (400 MHz, $CDCl_3$, major isomer): δ 7.37-6.99 (m, 20H, Ar), 5.83 (d, 1H, *J*=8.1 Hz, 2-NH), 5.27 (d, 1H, *J*=12.0 Hz, OCH_2), 5.17 (d, 1H, *J*=12.0 Hz, OCH_2), 5.05 (s, 2H, OCH_2), 4.07 (m, 1H, 2'-H), 3.56 (d, 1H, *J*=14.5 Hz, 4-CH_2), 3.43 (q, 1H, *J*=7.6 Hz, 3-H), 3.03 (d, 1H, *J*=14.5 Hz, 4-CH_2), 2.99 (m, 2H, 1'-H), 2.71 (dd, 1H, *J*=13.5, 7.4 Hz, 3'-H), 2.64 (dd, 1H, *J*=13.4, 6.6 Hz, 3'-H), 1.08 (d, 3H, *J*=7.5 Hz, 3-CH_3). ^{13}C NMR (100 MHz, $CDCl_3$, major isomer): δ 171.3 (COO), 170.3 (C2), 156.3 (OCON), 137.5, 135.1, 134.8, 129.7, 129.5, 129.2, 129.0, 128.9, 128.8, 128.7, 128.5, 128.4, 127.9, 127.5, 126.5 (Ar), 69.0 (C4), 67.8, 66.3 (OCH_2), 54.1 (C3), 51.6 (C2'), 47.0 (C1'), 41.2 (4-CH_2). 39.3 (C3'), 10.6 (3-CH_3). MS $(ES)^+$: 577.3 $[M+H]^+$. Exact Mass calculated for $C_{36}H_{36}N_2O_5$: 576.26242; found: 576.26124.

4*R*-Benzyl-4-benzyloxycarbonyl-3*R*,*S*-methyl-1-[(2'*R*-benzyloxycarbonylamino-3'-phenyl)prop-1'-yl]-2-oxoazetidine (31ab).

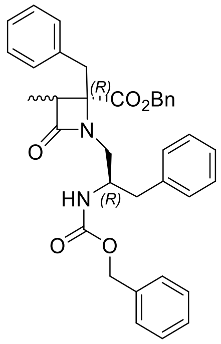

Syrup. Yield: 44% (from 28, B: BTPP). Eluent: EtOAc:Hexane (1:3). HPLC: tR = 16.20 min (gradient of 30% to 95% of A, in 20 min). Isomers ratio M(3*R*,4*R,2'R*):m(3*S*,4*R,2'R*) = 77:23. ^{1}H NMR (400 MHz, $CDCl_3$, major isomer): δ 7.35-6.96 (m, 20H, Ar), 5.75 (d, 1H, *J*=7.4 Hz, 2-NH), 5.24 (d, 1H, *J*=12.0 Hz, OCH_2), 5.14 (d, 1H, *J*=12.0 Hz, OCH_2), 5.05 (s, 2H, OCH_2), 4.17 (m, 1H, 2'-H), 3.43 (d, 1H, *J*=14.5 Hz, 4-CH_2), 3.23 (dd, 1H, *J*=14.5, 8.0, 1'-H), 3.11 (q, 1H, *J*=7.6 Hz, 3-H), 3.05 (d, 1H, *J*=14.5 Hz, 4-CH_2), 3.00 (dd, 1H, *J*=14.5, 4.0 Hz, 1'-H), 2.86 (dd, 1H, *J*=14.0,

8.0 Hz, 3'-H), 2.72 (dd, 1H, *J*=14.0, 7.0 Hz, 3'-H), 1.08 (d, 3H, *J*=7.5 Hz, 3-CH_3).^{13}C NMR (75 MHz, $CDCl_3$, major isomer): δ171.0 (COO), 170.6 (C2), 156.0 (OCON), 137.8, 137.0, 134.9, 134.8, 129.7, 129.2, 128.9, 128.85, 128.8, 128.7, 128.5, 128.45, 127.9, 127.8, 127.5, 126.5 (Ar), 68.7 (C4), 67. 7, 66.3 (OCH_2), 53.8 (C3), 51. 9 (C2'), 46.1 (C1'), 40.7 (4-CH_2). 39.1 (C3'), 10.6 (3-CH_3). MS $(ES)^+$: 577.18 $[M+H]^+$. Exact Mass calculated for $C_{36}H_{36}N_2O_5$: 576.26242; found:576.26364.

4*S*-Benzyloxycarbonyl-3*S*,4*S*-dimethyl-1-[(2'*R*-benzyloxycarbonylamino-3'-phenyl)prop-1'-yl]-2-oxoazetidine (39a).

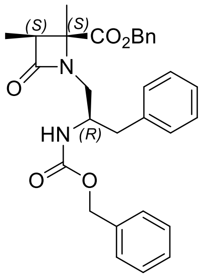

Syrup. Yield: 30% (from 37, B: BTPP). Eluent: EtOAc:Hexane (1:2). HPLC: tR = 17.49 min (gradient of 5% to 100% of A, in 20 min). $[\alpha]_D$ = +13.49 (c 1, $CHCl_3$). ^{1}H NMR (400 MHz, $CDCl_3$): δ 7.35- 7.15 (m, 15H, Ar), 5.36 (d, 1H, *J*=8.4 Hz, 2-NH), 5.18 (d, 1H, *J*=12.1 Hz, OCH_2), 5.12 (d, 1H, *J*=12.1 Hz, OCH_2), 5.03 (s, 2H, OCH_2), 4.10 (m, 1H, 2'-H), 3.32 (dd, 1H, *J*=14.6, 8.1 Hz, 1'-H), 3.22 (dd, 1H, *J*=14.6, 5.1 Hz, 1'-H), 2.96 (q, 1H, *J*=7.5 Hz, 3-H), 2.87 (dd, 1H, *J*=13.8, 6.2 Hz, 3'-H), 2.81 (m, 1H, 3'-H), 1.58 (s, 3H, CH_3), 1.06 (d, 3H, *J*=7.5 Hz, CH_3). ^{13}C NMR (75 MHz, $CDCl_3$): δ 171.5 (COO), 170.0 (C2), 156.2 (OCON), 137.3, 136.8, 135.1, 129.5, 128.85, 128.8, 128.7, 128.5, 128.1, 126.7 (Ar), 67.5, 66.6 (OCH_2), 64.8 (C4), 56.1 (C3), 51.7 (C2'), 44.6 (C1'), 39.2 (C3'), 20.8 (4-CH_3), 10.4 (CH_3). MS $(ES)^+$: 501.4 $[M+H]^+$. Exact Mass calculated for $C_{30}H_{32}N_2O_5$: 500.23112; found: 500.23117.

5*S*-Benzyl-4-benzyloxycarbonyl-1-[(1'*R*,*S*-benzyloxycarbonyl-2'-phenyl)et-1'-yl]-2-oxopiperazine (13ab).

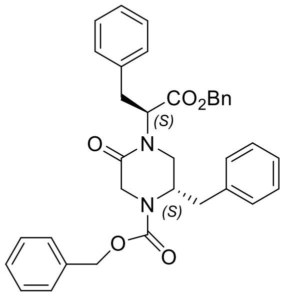

Syrup. Yield: 77% (from **10**, B: BTPP). Eluent: EtOAc:Hexane (1:3). HPLC: tR = 21.56 min (gradient of 30% to 95% of A, in 30 min). Isomers ratio

M(5*S,1'S*):m(5*S,1'R*)= 80:20. ^{1}H NMR (400 MHz, DMSO-d_6, 90 °C, major isomer): δ 7.35-7.02 (m, 20H, Ar), 5.18 (s, 2H, OCH_2), 5.13 (m, 1H, 1'-H), 4.93 (s, 2H, OCH_2), 4.34 (m, 1H, 5-H), 4.08 (d, 1H, *J*=18.0 Hz,, 3-H), 3.74 (d, 1H, *J*=18.0 Hz, 3-H), 3.53 (dd, 1H, *J*=12.6, 4.9 Hz, 2'-H), 3.31 (dd, 1H, *J*=14.4, 6.1 Hz, 6-H), 3.17 (m, 2H, 2'-H, 6-H), 2.65 (dd, 1H, *J*=14.2, 8.1 Hz, 5-CH_2), 2.50 (dd, 1H, *J*=14.2, 8.1 Hz, 5-CH_2). ^{13}C NMR (100 MHz, DMSO-d_6, 90 °C, major isomer): δ 169.4 (COO), 165.0 (CON), 153.4 (OCON), 137.2, 136.7, 136.02, 135.3, 128.5, 128.4, 128.0, 127.95, 127.9, 127.8, 127.7, 127.6, 127.3, 127.0, 126.1, 125.9 (Ar), 66.2, 65.6 (OCH_2), 58.1 (C1'), 50.6 (C5), 47.6 (C6), 44.2 (C3), 35.3 (5-CH_2), 33.6 (C2'). MS (ES)$^+$: 563.31[M+H]$^+$. Exact Mass calculated for $C_{36}H_{36}N_2O_5$: 562.24677; found: 562.24814.

5*R*-Benzyl-4-benzyloxycarbonyl-1-[(1'*R,S*-benzyloxycarbonyl-2'-phenyl)et-1'-yl]-2-oxopiperazine (15ab).

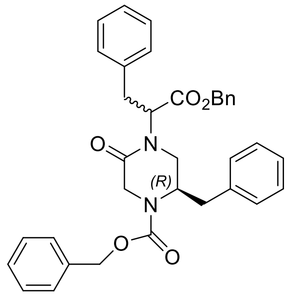

Syrup. Yield: 77% (from **11**, B: BTPP). Eluent: EtOAc:Hexane (1:3). HPLC: tR = 21.47 min (gradient of 30% to 95% of A, in 30 min). Isomers ratio M(5*R,1'S*):m(5*R,1'R*)= 81:19. ^{1}H NMR (400 MHz, DMSO-d_6, 90 °C, major isomer): δ 7.35-6.98 (m, 20H, Ar), 5.17 (s, 2H, OCH_2), 4.98 (d, 1H, *J*= 12.6 Hz, OCH_2), 4.91 (d, 1H, *J*= 12.6 Hz, OCH_2), 4.85 (dd, 1H, *J*= 9.9, 5.8 Hz, 1'-H), 4.23 (m, 1H, 5-H), 4.19 (d, 1H, *J*=18.0 Hz, 3-H), 3.70 (d, 1H, *J*=18.0 Hz, 3-H), 3.29 (dd, 1H, *J*=14.1, 6.2 Hz, 2'-H), 3.16 (m, 2H, β-Phe, 6-H), 2.99 (dd, 1H, *J*=12.7, 4.7 Hz, 6-H), 2.63 (m, 2H, 5-CH_2). ^{13}C NMR (100 MHz, DMSO-d_6, 90 °C, major isomer): δ 169.0 (COO), 164.7 (CON), 153.4 (OCON), 137.0, 136.8, 136.1, 135.4, 128.5, 128.4, 127.95, 127.9, 127.8, 127.7, 127.6, 127.5, 127.4, 127.0, 126.1, 125.9 (Ar), 66.2, 66.0 (OCH_2), 59.7 (C1'), 50.7 (C5), 48.3 (C6), 44.4 (C3), 35.1 (5-CH_2), 33.3 (C2'). MS (ES)$^+$: 563.32 [M+H]$^+$. Exact Mass calculated for $C_{35}H_{34}N_2O_5$: 562.24677; found: 562.24725.

5*S*-Benzyl-4-benzyloxycarbonyl-1-[(1'*R,S*-metoxycarbonyl-2'-phenyl)et-1'-yl]-2-oxopiperazine (20ab).

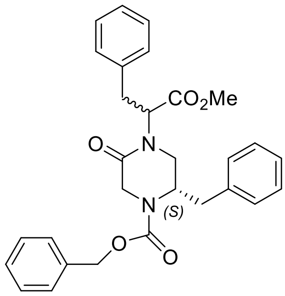

Syrup. Yield: 60% (from **19**, B: Cs_2CO_3). Eluent: EtOAc:Hexane (1:3). HPLC: tR = 16.99 min (gradient of 30% a 95% de A, en 30 min). Isomers ratio M(5*S,1'R*):m(5*S,1'S*)=96:4. ^{1}H NMR (400 MHz, DMSO-d_6, 90 °C, major isomer): δ 7.36-7.08 (m, 15H, Ar), 5.01 (d, 1H, *J*= 12.6 Hz, OCH_2), 4.93 (d, 1H, *J*= 12.6 Hz, OCH_2), 4.86 (dd, 1H, *J*= 9.9, 5.8 Hz, 1'-H), 4.27 (m, 1H, 5-H), 4.20 (d, 1H, *J*=18.0 Hz, 3-H), 3.72 (d, 1H, *J*=18.0 Hz, 3-H), 3.68 (s, 3H, OMe), 3.27 (dd, 1H, *J*=14.5, 5.7 Hz, 2'-H), 3.15 (m, 3H, 2'-H, 6-H), 2.71 (m, 1H, 5-CH_2), 2.50 (dd, 1H, *J*=14.2, 8.1 Hz, 5-CH_2). ^{13}C NMR (100 MHz, DMSO-d_6, 90 °C, major isomer): δ 169.6 (COO), 164.8 (CON), 153.4 (OCON), 137.1, 136.8, 136.1, 128.45, 128.4, 127.9, 127.85, 127.8, 127.4, 127.0, 126.1, 125.9 (Ar), 66.2 (OCH_2), 59.2 (C1'), 51.5 (OMe), 50.8 (C5), 48.0 (C6), 44.5 (C3), 35.2 (5-CH_2), 33.3 (C2'). MS (ES)$^+$: 487.31[M+H]$^+$. Exact Mass calculated for $C_{29}H_{30}N_2O_5$: 486.21547; found: 486.21684.

5*S*-Benzyl-4-benzyloxycarbonyl-1-[(1'*R*,*S*-benzyloxycarbonyl-2'-phenyl)et-1'-yl]-2-oxopiperazine (21ab). Benzyl alcohol (5.36 mmol, 570 µL) was added to a solution of ketopiperazine derivative 20ab (0.032mmol, 16mg) in HCl / dioxane 4M (1 mL) at room temperature for 48 hours. After this time, the solvent was evaporated to dryness and the obtained reaction crude is purified on silica gel column using as eluents EtOAc/Hexane (1: 3) to give 10 mg (55%) of the compound described below, as a syrup.

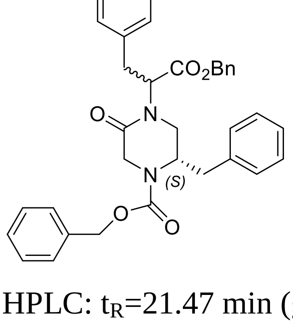

HPLC: t_R=21.47 min (gradient of 30% to 95% of A, in 30 min). Isomers ratio M(1'*R*,5*S*)/m(1'*S*,5*S*)=90:10. ^{1}H NMR (400 MHz, DMSO-d_6, 90 °C, major isomer): δ 7.35-6.98 (m, 20H, Ar), 5.17 (s, 2H, OCH_2), 4.98 (d, 1H, *J*= 12.6 Hz, OCH_2), 4.91 (d,

1H, *J*= 12.6 Hz, OCH_2), 4.85 (dd, 1H, *J*= 9.9, 5.8 Hz, 1'–H), 4.23 (m, 1H, 5-H), 4.19 (d, 1H, *J*=18.0 Hz, 3-H), 3.70 (d, 1H, *J*=18.0 Hz, 3-H), 3.29 (dd, 1H, *J*=14.1, 6.2 Hz, 2'–H), 3.16 (m, 2H, β-Phe, 6-H), 2.99 (dd, 1H, *J*=12.7, 4.7 Hz, 6-H), 2.63 (m, 2H, 5-CH_2). ^{13}C NMR (100 MHz, DMSO-d_6, 90 °C, major isomer): δ 169.0 (COO), 164.7 (CON), 153.4 (OCON), 137.0, 136.8, 136.1, 135.4, 128.5, 128.4, 127.95, 127.9, 127.8, 127.7, 127.6, 127.5, 127.4, 127.0, 126.1, 125.9 (Ar), 66.2, 66.0 (OCH_2), 59.7 (C1'), 50.7 (C5), 48.3 (C6), 44.4 (C3), 35.1 (5-CH_2), 33.3 (C2'). MS (ES)$^+$: 563.31[M+H]$^+$. Exact Mass calculated for $C_{35}H_{34}N_2O_5$: 562.24677; found: 562.24735.

5*S*-Benzyl-4-benzyloxycarbonyl-1-[(1'*R*,*S*-Benzyloxycarbonyl-2'-phenyl)et-1'-yl]-3*S*-methyl-2-oxopiperazine (26ab).

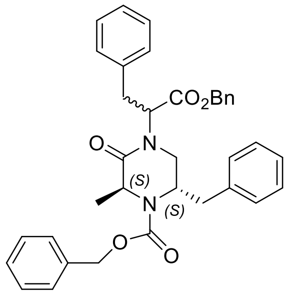

Syrup. Yield: 63% (from 23, B: BTPP). Eluent: EtOAc:Hexane (1:4). HPLC: tR = 17.38 min (gradient of 30% to 95% of A, in 20 min). Isomers ratio M(3*S*,5*S*,*1'S*):m(3*S*,5*S*,*1'R*) = 81:19. ^{1}H NMR (400 MHz, DMSO-d_6, 90 °C, major isomer): δ 7.37-6.98 (m, 20H, Ar), 5.31 (dd, 1H, *J*=10.7, 5.6 Hz, 1'-H), 5.24 (d, 1H, *J*=12.5 Hz, OCH_2), 5.17 (d, 1H, *J*=12.5 Hz, OCH_2), 4.98 (s, 2H, OCH_2), 4.15 (q, 1H, *J*=6.8 Hz, 3-H), 4.14 (m, 1H, 5-H), 3.51 (dd, 1H, *J*=13.2, 3.8 Hz, 6-H), 3.37 (dd, 1H, *J*=14.6, 5.6 Hz, 2'-H), 3.28 (dd, 1H, *J*=13.2, 2.0 Hz, 6-H), 3.18 (dd, 1H, *J*=14.6, 10.7 Hz, 2'-H), 2.61 (d, 2H, *J*=6.7 Hz, 5-CH_2), 0.92 (d, 3H, *J*=6.8 Hz, 3-CH_3). ^{13}C NMR (100 MHz, DMSO-d_6, 90 °C, major isomer): δ 169.5 (COO), 169.0 (CON), 153.2 (OCON), 137.9, 136.4, 135.9, 135.1, 128.75, 128.55, 128.65, 127.95, 127.9, 127.8, 127.7, 127.5, 127.45, 127.4, 126.2, 125.7 (Ar), 66.2, 66.1 (OCH_2), 57.8 (C1'), 52.7 (C5), 52.5 (C3), 44.7 (C6), 36.9 (5-CH_2), 34.1 (C2'), 18.4 (CH_3). MS (ES)$^+$: 577.25 [M+H]$^+$. Exact Mass calculated for $C_{36}H_{36}N_2O_5$: 576.26242; found: 576.26220.

5*R*-Benzyl-4-benzyloxycarbonyl-1-[(1'*R*,*S*-Benzyloxycarbonyl-2'-phenyl)et-1'-yl]-3*R*-methyl-2-oxopiperazine (30ab).

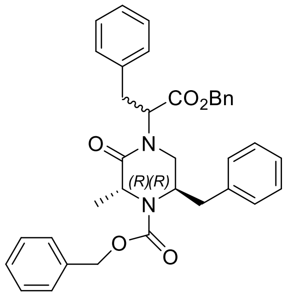

Syrup. Yield: 32% (from 27, B: BTPP). Eluent: EtOAc:Hexane (1:3). HPLC: tR = 17.64 min (gradient of 30% to 95% of A, in 20 min). Isomers ratio M(3*R*, 5*R*, *1'S*):m(3*R*,5*R,1'R*)= 82:18. ^{1}H NMR (400 MHz, DMSO-d_6, 90 °C, major isomer): δ 7.37-6.99 (m, 20H, Ar), 5.20 (s, 2H, OCH_2), 5.02 (s, 2H, OCH_2), 4.63 (dd, 1H, *J*=9.8, 5.8 Hz, 1'-H), 4.17 (q, 1H, *J*=6.9 Hz, 3-H), 3.99 (m, 1H, 5-H), 3.36 (dd, 1H, *J*=14.6, 5.6 Hz, 2'-H), 3.29 (m, 1H, 2'-H), 2.92 (m, 2H, 6-H), 2.79 (dd, 1H, *J*=13.9, 9.7 Hz, 5-CH_2), 2.60 (m, 1H, 5-CH_2), 1.22 (d, 3H, *J*=6.8 Hz, 3-CH_3). ^{13}C NMR (100 MHz, DMSO-d_6, 90 °C, major isomer): δ 168.8 (COO), 168.1 (CON), 153.0 (OCON), 137.6, 136.9, 136.0, 135.3, 128.8, 128.6, 128.5, 128.0, 127.95, 127.9, 127.8, 127.7, 127.5, 127.4, 126.2, 125.8 (Ar), 66.3, 66.1 (OCH_2), 61.9 (C1'), 53.0 (C5), 52.9 (C3), 47.3 (C6), 36.1 (5-CH_2), 33.7 (C2'), 18.8 (CH_3). MS $(ES)^+$: 577.25 $[M+H]^+$. Exact Mass calculated for $C_{36}H_{36}N_2O_5$: 576.26242; found: 576.26415.

5*R*-Benzyl-4-benzyloxycarbonyl-1-[(1'*R*,*S*-Benzyloxycarbonyl-2'-phenyl)et-1'-yl]-3*S*-methyl-2-oxopiperazine (32ab).

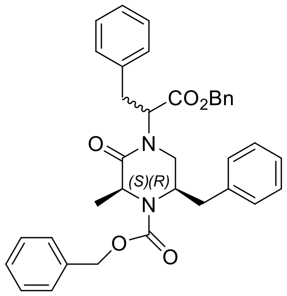

Syrup. Yield: 21% (from 28, B: BTPP). Eluent: EtOAc:Hexane (1:3). HPLC: tR = 17.22 min (gradient of 30% to 95% of A, in 20 min). Isomers ratio M(3*S*,5*R*, *1'S*):m(3*S*, 5*R*, *1'R*)= 85:15. ^{1}H NMR (400 MHz, DMSO-d_6, 90 °C, major isomer): δ 7.40-6.97 (m, 20H, Ar), 5.20 (d, 1H, *J*=12.4 Hz, OCH_2), 5.13 (d, 1H, *J*=12.4 Hz, OCH_2), 5.09 (s, 2H, OCH_2), 4.78 (dd, 1H, *J*=10.2, 5.6 Hz, 1'-H), 4.39 (q, 1H, *J*=7.1 Hz, 3-H), 4.11 (m, 1H, 5-H), 3.27 (dd, 1H, *J*=14.2, 5.6 Hz, 2´-H), 3.14 (m, 2H, 2'-H, 6-H), 2.94 (dd, 1H, *J*=13.0, 5.0 Hz, 6-H), 2.73 (dd, 1H, *J*=13.6, 8.3 Hz, 5-CH_2), 2.66 (m, 1H, 5-CH_2), 1.11

(d, 3H, *J*=7.2 Hz, 3-CH_3). MS $(ES)^+$: 577.25 $[M+H]^+$. Exact Mass calculated for $C_{36}H_{36}N_2O_5$: 576.26242; found: 576.26352.

5*R*-Benzyl-1-[(1'*R,S*-benzyloxycarbonyl)et-1'-yl]-4-benzyloxycarbonyl-2-oxopiperazine (38ab).

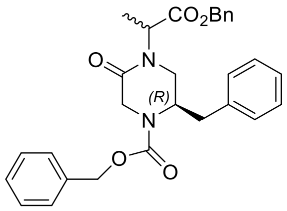

Syrup. Yield: 81% (from 36, B:BTPP). Eluent: EtOAc:Hexane (1:3). HPLC: tR = 15.57 min (gradient of 5% to 100% de A, in 20 min). Isomers ratioM(5*R,1'S*)/m(5*R,1'R*)= 86:14. ^{1}H NMR (400 MHz, DMSO-d_6, 90 °C, major isomer): δ 7.37-7.13 (m, 15H, Ar), 5.14 (s, 2H, OCH_2), 5.03 (d, 1H, *J*=12.6 Hz, OCH_2), 4.95 (d, 1H, *J*=12.6 Hz, OCH_2), 4.84 (q, 1H, *J*=7.1 Hz, 1'-H), 4.49 (m, 1H, 5-H), 4.22 (d, 1H, *J*=18.0 Hz, 3-H), 3.83 (d, 1H, *J*=18.0 Hz, 3-H), 3.53 (dd, 1H, *J*=12.7, 4.5 Hz, 6-H), 3.23 (dd, 1H, *J*=12.7, 2.8 Hz, 6-H), 2.85 (d, 2H, *J*=7.4 Hz, 5-CH_2), 1.36 (d, 3H, *J*=7.2 Hz, 2'-H). ^{13}C NMR (100 MHz, DMSO-d_6, 90 °C, major isomer): δ 170.0 (COO), 164.4 (CON), 153.4 (OCON), 137.2, 136.1, 135.5, 128.5, 128.0, 127.9, 127.6, 127.8, 127.4, 127.3, 127.1, 126.0 (Ar), 66.2, 65.9 (OCH_2), 52.7 (C1'), 50.8 (C5), 45.7 (C6), 44.5 (C3), 35.3 (5-CH_2), 13.4 (C2'). MS $(ES)^+$: 487.31 $[M+H]^+$. Exact Mass calculated for $C_{29}H_{30}N_2O_5$: 486.21547; found: 486.21515.

5*R*-Benzyl-4-benzyloxycarbonyl-1-[(1'*R,S*-benzyloxycarbonyl)et-1'-yl]-3*R*-methyl-2-oxopiperazine (40ab).

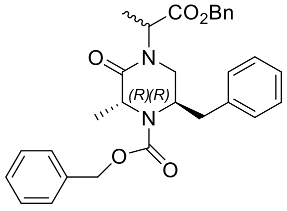

Syrup. Yield: 41% (from 37, B: BTPP). Eluent: EtOAc:Hexane (1:3). HPLC: tR = 19.22 min (gradient of 30% a 95% de A, en 300 min). Isomers ratio M(3*R*,5*R,1'S*):m(3*R*,5*R,1'R*)= 58:42. ^{1}H NMR (400 MHz, DMSO-d_6, 90 °C, major isomer): δ 7.38-7.11 (m, 15H, Ar), 5.19 (d, 1H, *J*=12.7 Hz, OCH_2), 5.14 (s, 2H, OCH_2), 5.08 (d, 1H, *J*=12.6 Hz, OCH_2), 4.82 (q, 1H, *J*=7.2 Hz, 1'-H), 4.24 (m, 2H, 5-H, 3-H), 3.57 (dd, 1H, *J*=13.1, 3.6 Hz, 6-H), 3.13 (dd, 1H, *J*=13.1, 2.1 Hz, 6-H), 2.79 (m, 2H, 5-CH_2), 1.33 (d, 3H, *J*=6.9 Hz, 3-CH_3), 1.30 (d, 3H, *J*=7.2 Hz, 2'-H). ^{13}C NMR (100

MHz, DMSO-d_6, 90 °C, major isomer): δ 169.9 (COO), 168.3 (CON), 153.2 (OCON), 137.4, 136.1, 135.3, 128.5, 128.0, 127.95, 127.9, 127.55, 127.5, 127.45, 127.4, 125.9 (Ar), 66.3, 66.0 (OCH_2), 54.2 (C1'), 52.8 (C5), 44.8 (C6), 42.9 (C3), 36.8 (5-CH_2), 18.7 (3-CH_3), 13.4 (C2'). MS $(ES)^+$: 501.30 $[M+H]^+$. Exact Mass calculated for $C_{30}H_{32}N_2O_5$: 500.23112; found: 500.23234.

Scheme S1. Preparation of 2-cloroalkanoyl phenylalaninol-Ala conjugates and their cyclization

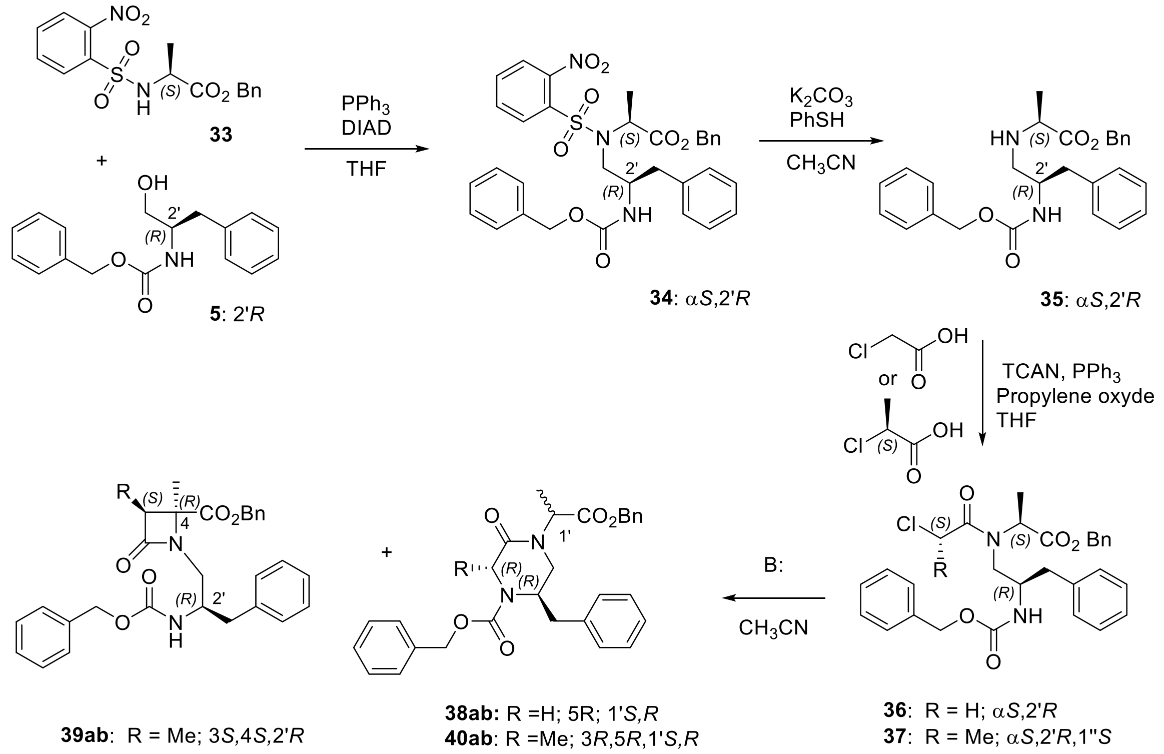

Removal of *C*-terminal alkyl groups from compound 13ab and synthesis of dipeptide derivatives 41.

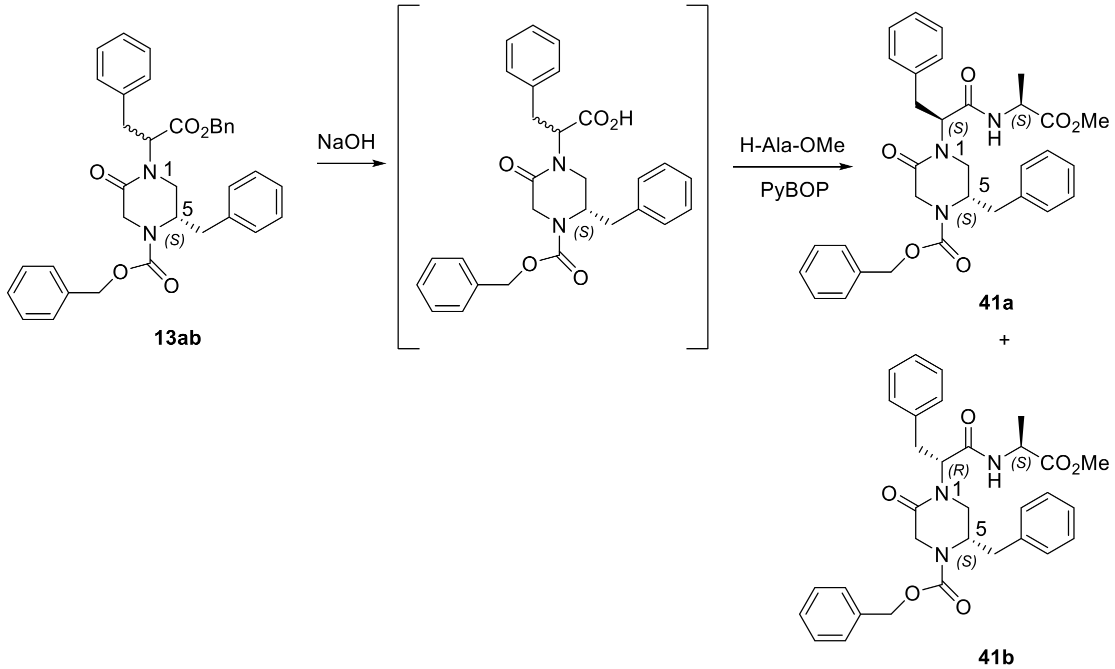

(5*S*,1'*S*,1''*S*)- and (5*S*,1'*R*,1''*S*)-5-Benzyl-4-benzyloxycarbonyl-1-[1'-((1''-metoxycarbonyl)ethyl)carbamoyl-2'-phenyl)et-1'-yl]-2-oxopiperazine (41ab)

A solution of the corresponding 4-benzyloxycarbonyl derivative **13ab** (0.24 mmol) in MeOH (3 mL) was treated with 2 N NaOH (0.36 mmol, 0.18 mL), and the mixture was stirred overnight at room temperature. After evaporation of the MeOH the remaining aqueous mixture was diluted with H_2O (5 mL), acidified with 1 N HCl to pH 3, and extracted with EtOAc. The extract was dried (Na_2SO_4) and evaporated. A solution of the corresponding 4-carboxy derivative (0.33 mmol) and Ala-OMe.HCl (0.33 mmol, 46 mg), in dry THF (4 mL) was successively treated with PyBOP (0.33 mmol, 0.172 mg) and TEA (92 µl, 0.66 mmol) at room temperature. The stirring was continued until complete disappearance of the starting material (2 days). The isomers ratio was determined by HPLC on the crude reaction mixtures. To characterize the obtained dipeptide derivatives, the solvent was evaporated, and the residue was dissolved in EtOAc and washed with citric acid (10%), $NaHCO_3$ (10%), and brine. The organic layer was dried (Na_2SO_4) and evaporated, leaving a residue which was purified on a silica gel column using as eluents MeOH :CH_2Cl_2 (1:30) to give 62 mg (68%) of the compound described below , as a syrup. Isomers ratio M(5*S,1'S*)/m(5*S,1'R*)= 75:25. HPLC (major isomer): t_R= 6.94 min (gradient of 30% to 95% of A, in 10 min).^{1}H NMR (400 MHz, DMSO-d$_6$, 90 °C, major isomer): δ 8.29 (d, 1H, *J*=7.3 Hz, NHCO), 7.34-7.06 (m, 15H,

Ar), 5.36 (m, 1H, 1'-H), 5.01-4.82 (m, 2H, OCH_2), 4.36 (q, 1H, *J*=7.3 Hz, 1''-H), 4.26 (m, 1H, 5-H), 4.05 (d, 1H, *J*=17.7 Hz, 3-H), 3.73 (d, 1H, *J*=17.6 Hz, 3-H), 3.63 (s, 3H, OMe), 3.47 (m, 2H, 2'H), 3.23 (dd, 1H, J= 14.9, 5.7, 6-H), 2.91 (dd, 1H, J= 14.8, 9.9, 6-H), 2.86 (dd, 1H, *J*=13.9, 7.7 Hz, 5-CH_2), 2.78 (dd, 1H, *J*=13.7, 6.6 Hz, 5-CH_2), 1.32 (d, 3H, *J*=7.2 Hz, CH_3). HPLC (minor isomer): t_R=7.12 min (gradient of 30% to 95% of A, in 10 min).^{1}H NMR (400 MHz, DMSO-d_6, 90 °C, minor isomer): δ 8.22 (d, 1H, *J*=7.3 Hz, NHCO), 7.34-7.06 (m, 15H, Ar), 5.36 (m, 1H, 1'-H), 5.01-4.82 (m, 2H, OCH_2), 4.42 (m, 1H, 5-H), 4.34 (q, 1H, *J*=7.2 Hz, 1''-H), 4.23 (d, 1H, *J*=17.9 Hz, 3-H), 3.68 (d, 1H, *J*=18.0 Hz, 3-H), 3.59 (s, 3H, OMe), 3.55 (dd, 1H, J = 13.0, 4.9, 2'-H), 3.47 (m, 1H, 2'-H), 3.29 (dd, 1H, J = 12.5, 3.7, 6-H), 2.88 (m, 1H, 6-H), 2.63 (dd, 1H, *J*=14.0, 6.7 Hz, 5-CH_2), 2.56 (dd, 1H, *J*=14.0, 7.9 Hz, 5-CH_2), 1.24 (d, 3H, *J*=7.2 Hz, CH_3). MS (ES)$^+$: 558.41[M+H]$^+$.

^{1}H-NMR spectrum (400 MHz, DMSO-*d*$_6$) 90 °C (a:b, *S,S,S/R,S,S*)=75:25 (41ab)

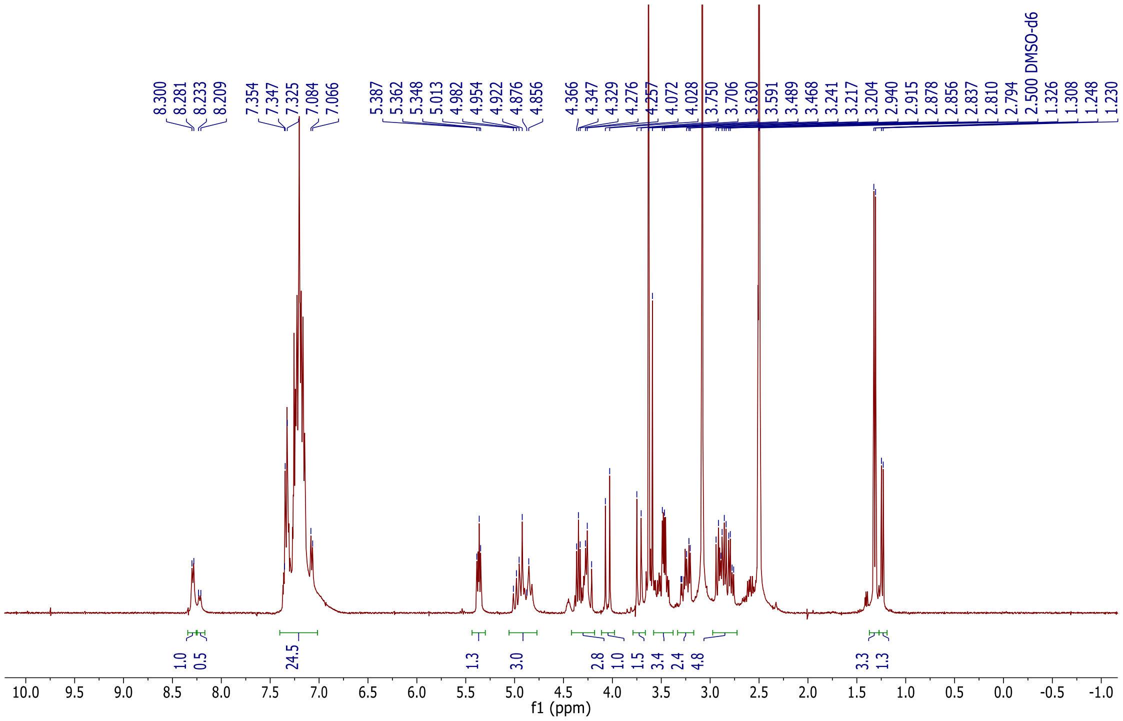

Epimerization studies

To explain the unexpected formation of diastereoisomeric β-lactams, three possible situations could be contemplated: i) the epimerization at position 1'' in the precursor 2-chloropropanoyl derivative in the reaction media, ii) the cyclization through a SN1 mechanism of the enantiopure 2-chloropropanoyl intermediates, or iii) the epimerization at position 3 of the β–lactam once formed. Among them, the first possibility was discarded because cyclization of α*S*,2'*R*,1''*R* intermediate **28** should give the 3*R*,4*R*, 2'*R*–lactam **31a** plus its 3*S*,4*S*,2'*R* isomer, enantiomers of **24a** and **25a**, respectively. However, while the ^{1}H NMR of the major isomer **31a** is identical to that of the *SSS* enantiomer **24a**, the chemical shifts of the minor diastereoisomer did not correspond to that of **25a**. The second option appeared less probable since four diastereoisomers could be expected. The third scenario seemed the most plausible, because the cyclization of **27** with BTTP (for 5 h) afforded pure β-lactam **29a**, while reaction with Cs_2CO_3 (for 168 h) led to a mixture of diastereoisomers **29ab** (**a**:**b**, 73:27). In addition, β-lactams **25** and **29** were configurationally unstable in the presence of base (Figures S1 and S2). Thus, a pure sample of compounds **29a** led to a mixture of **29ab** (3*S*,4*S*,2'*R*/3*R*,4*S*,2'*R*, 88:12) after treatment with Cs_2CO_3 (1 equiv) for 6 days (Figure S2). More dramatically, a similar treatment of a mixture of the β–lactam **25ab** (3*R*,4*R*,2'*S*/3*S*,4*R*,2'*S*, 72:28) was converted into a 40:60 mixture after 6 days, and total conversion to the 3*S*,4*R*,2'*S* isomer **25b** (0:100) was observed after 20 days (Figures S1).

Treatment of isolated β–lactams 25ab and 29a with Cs_2CO_3.

The indicated compounds (0.014 mmol) in ACN (1 mL) were treated with Cs_2CO_3 (0,021 mmol) and stirred at room temperature for 20 days. Evolution of these compounds was followed and quantified by analytical HPLC.

Compound **25ab**

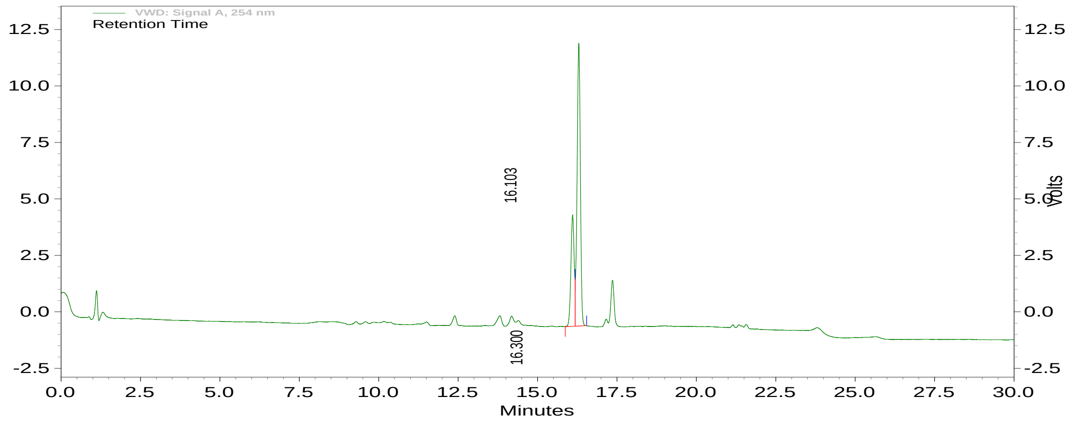

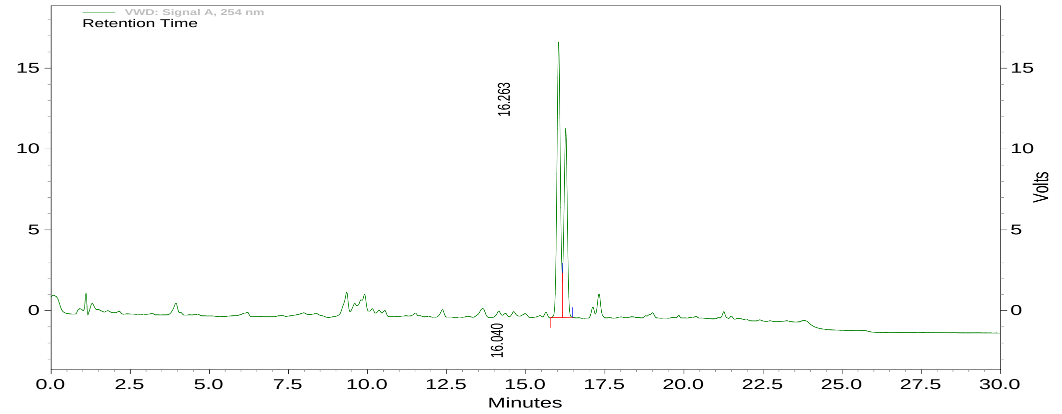

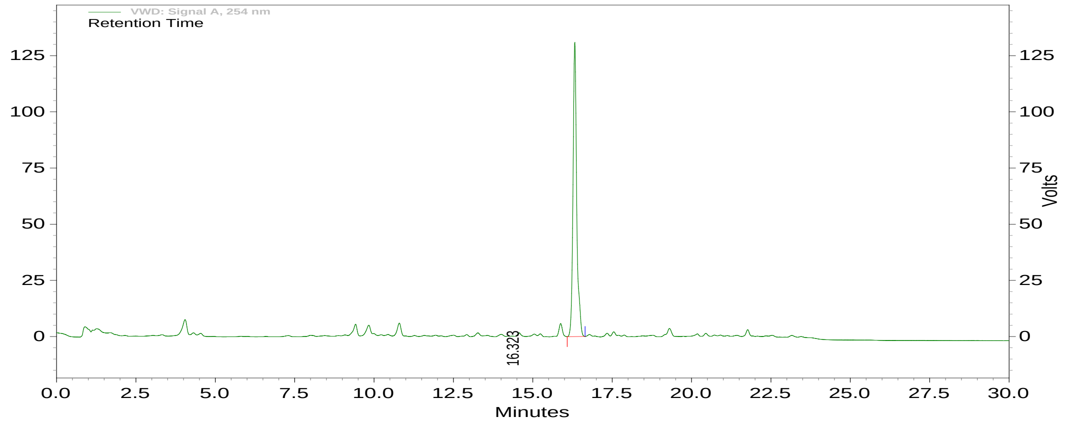

Figure S1. Follow up of the treatment of compound **25ab** with Cs_2CO (HPLC). Initial mixture (up). After 6 days (center). After 20 days (bottom).

Compound **29a**

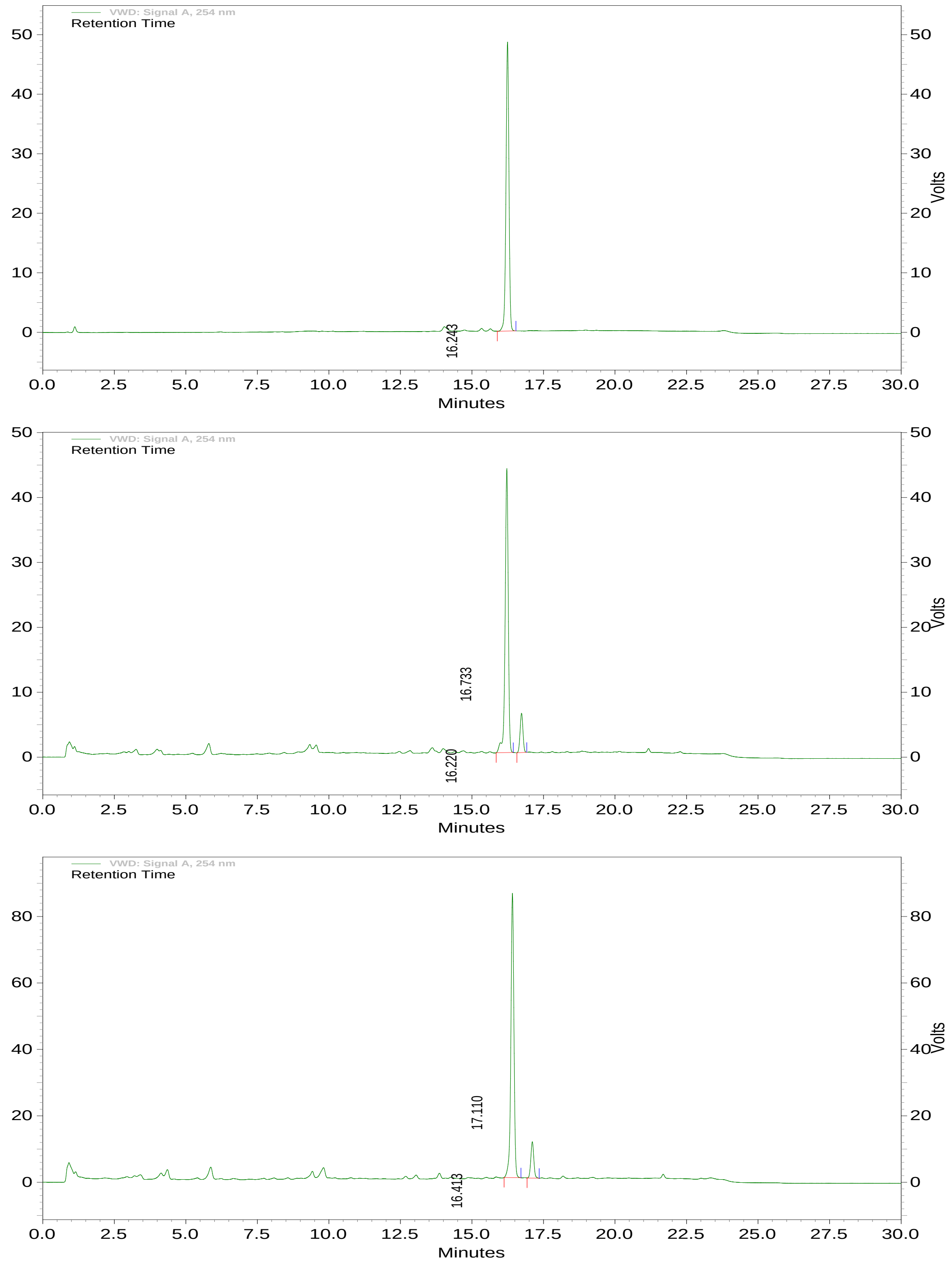

Figure S2. Follow up of the treatment of compound **29a** with Cs_2CO (HPLC). Initial pure compound (up). After 6 days (center). After 20 days (bottom).

Biological Activity

Table S1. Activity at hTRPV1 of β–lactams derived from phenylalaninol conjugates

Compd.	Isomer	Isomer ratio	**TRPV1 Blockade (%)**	
			**50 µM**	**5 µM**
**12ab**	4*S*,2'*S*/4*R*,2'*S*	83:17	15.3±2.8	9.2±1.4
**14ab**	4*S*,2'*R*/4*R*,2'*R*	88:12	8.7±3.5	4.1±2.4
**24a**	3*S*,4*S*,2'*S*	–	11.8±3.2	9.7±1.1
**25ab**	3*R*,4*R*,2'*S*/3*S*,4*R*,2'*S*	85:15	4.3±1.1	3.4±1.7
**29a**	3*S*,4*S*,2'*R*	–	16.2±4.7	8.2±2.1
**31ab**	3*R*,4*R*,2'*R*/3*S*,4*R*,2'*R*	77:23	12.1±1.3	5.7±2.3
**39a**	3*S*,4*S*,2'*R*	–	3.2±2.777	2.6±0.9

Table S2. Activity at hTRPV1 of 2-ketopiperazines derived from phenylalaninol conjugates

Compd.	Isomer	Isomer ratio	**TRPV1 Blockade (%)**	
			**50 µM**	**5 µM**
**13ab**	5*S*,1'*S*/5*S*,1'*R*	80:20	8.1±3.4	7.2±3.8
**15ab**	5*R*,1'*S*/5*R*,1'*R*	81:19	7.8±4.1	6.3±2.1
**20ab**	5*S*,1'*S*/5*S*,1'*R*	4:96	19.3±1.2	12.2±3.1
**21ab**	5*S*,1'*S*/5*S*,1'*R*	10:90	11.8±1.8	6.2±3.1
**26ab**	3*S*,5*S*,1'*S*/3*R*,5*S*,1'*R*	81:19	7.3±3.2	6.2±1.8
**30ab**	3*R*,5*R*,1'*S*/3*R*,5*R*,1'*R*	82:18	13.9±4.8	9.2±2.1
**32ab**	3*S*,5*R*,1'*S*/3*S*,5*R*,1'*R*	96:4	6.3±2.3	4.1±2.3

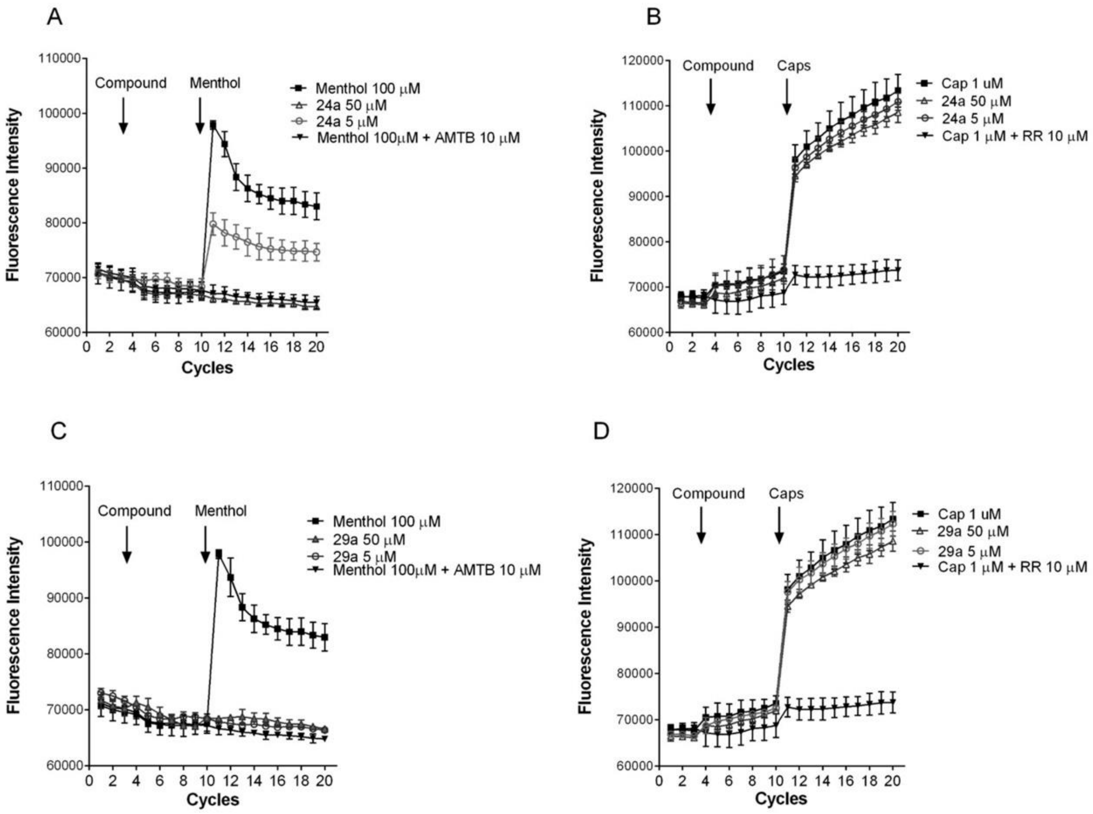

Figure S3. Effect of compounds on TRP activity. Representative fluorescence registers for compound **24a** at 50 and 5 µM on HEK-rTRPM8 (A) and HEK-hTRPV1 cells (B**)**. Representative fluorescence registers for compound **29a** at 50 and 5 µM on HEK-rTRPM8 (C) and HEK-hTRPV1 cells (D**)**.

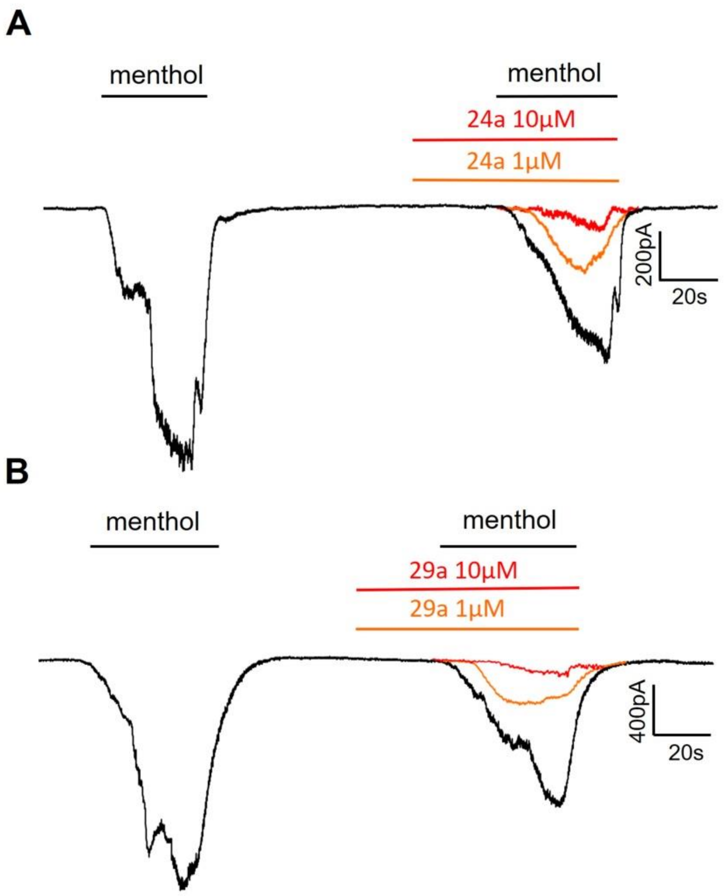

Figure S4. Effect of compounds 24a and 29a on TRPM8 activity. Representative menthol-evoked rTRPM8 inward current recorded at a holding potential of -60 mV for control condition with two pulses of 100 µM menthol (black traces), after 30 s application of compound at 10 µM (red traces) or at 1 µM (orange traces) before the second menthol pulse.

Molecular Modeling

Table S3. Main sites found for compounds **13a**, **24a** and **29a**

Compd	Site 1			Site 2		
	E (Kcal/mol)	% solutions	First cluster	E(Kcal/mol)	% solutions	First cluster
**13a**	9.81	62	1	9.59	14	2
**24a**	8.68	54	3	9.02	10	1
**29a**	9.00	54	1	8.58	12	2

% Clusters = Refers to the percentage of solutions that dock in a similar region (it does not necessarily have to be the same links, but it moves in a very similar area)
First Cluster = It is the position in which this solution appears for the first time.

Site location
Site 1: Inner Pore (contacts with S5 and S6 from two different subunits)
Site 2: Internal pore (bottom part of the four subunits)

Other sites
Site 3: Internal pore (in between sites 1 and 2)
Site 4: External pore
Site 5: S1, S4, and S6 from one subunit and S5 from other subunit
Site 6: S2 and TRP domain

>80% solutions indicated the pore zone as the interaction point for all compounds

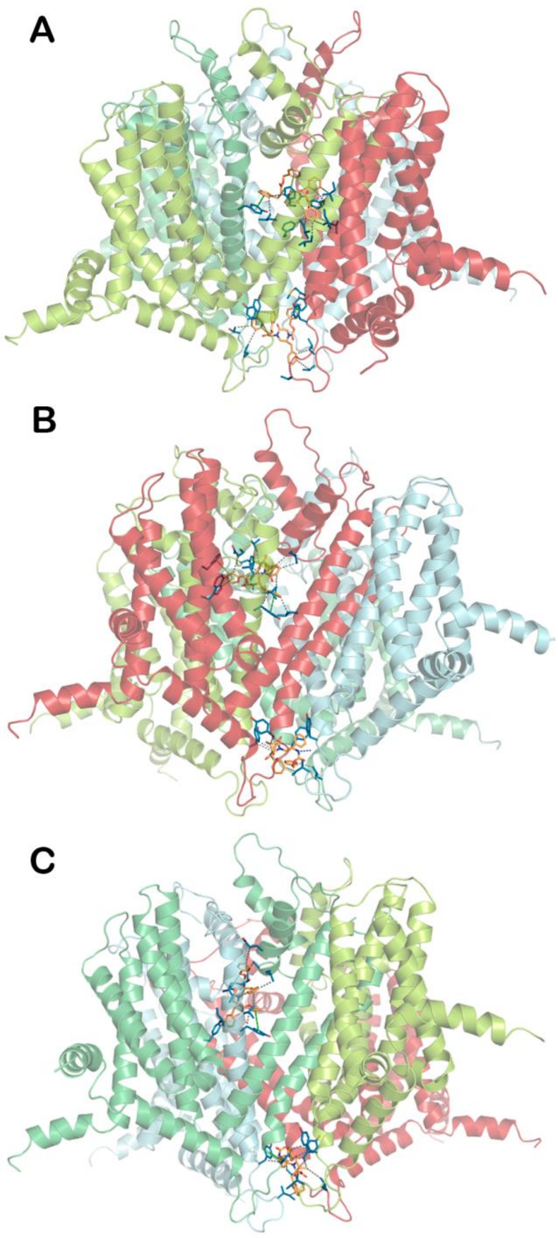

Figure S5. Global overview of the binding of compounds **13a** (A), **24a** (B) and **29a** (C) to TRPM8. Site 1 (up) and Site 2 (down) in each image.

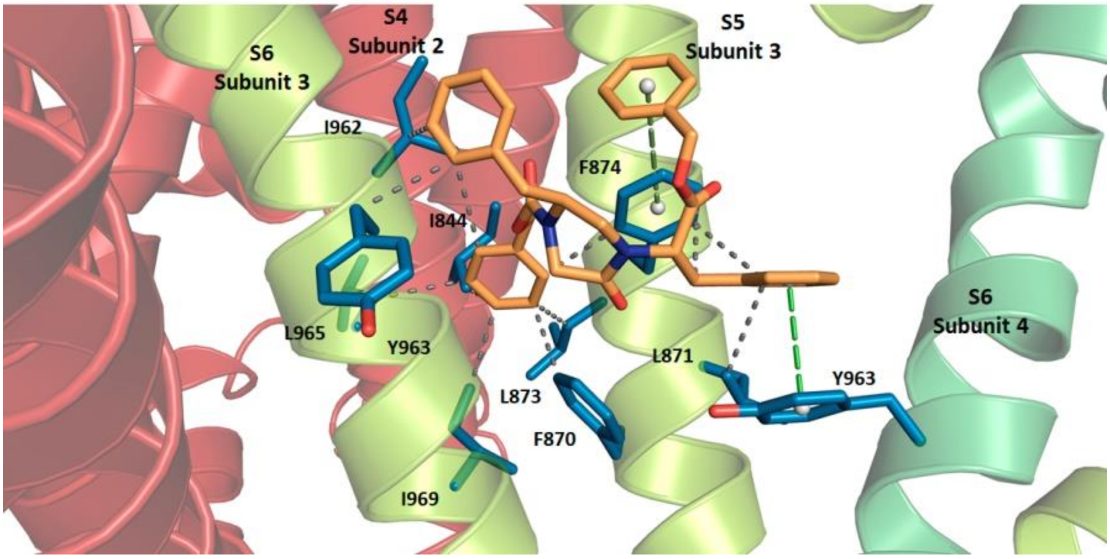

Hydrophobic Interactions

Index	Residue	AA	Distance	Ligand Atom	Protein Atom
1	844C -Sub2	ILE	3.43	21022	12612
2	870B -Sub3	PHE	3.43	21023	7821
3	871B -Sub3	LEU	3.86	21006	7842
4	873B -Sub3	LEU	3.91	21023	7872
5	874B -Sub3	PHE	3.51	21024	7895
6	874B -Sub3	PHE	3.90	21006	7897
7	874B -Sub3	PHE	3.96	21000	7901
8	962B -Sub3	ILE	3.77	21020	9323
9	962B -Sub3	ILE	3.80	21016	9318
10	963B -Sub3	TYR	3.40	21015	9337
11	965B -Sub3	LEU	3.41	21021	9375
12	969B -Sub3	ILE	3.21	21022	9442

π-Stacking

Index	Residue	AA	Distance	Angle	Offset	Type	Ligand Atoms
1	874B-Sub3	PHE	5.00	88.52	0.57	T	21028, 21029, 21030, 21031, 21032, 21033
2	963A-Sub4	TYR	3.99	16.97	0.38	P	21001, 21002, 21003, 21004, 21005, 21006

Figure S6. Detailed view of the binding of compounds **13a** to TRPM8 Site 1.

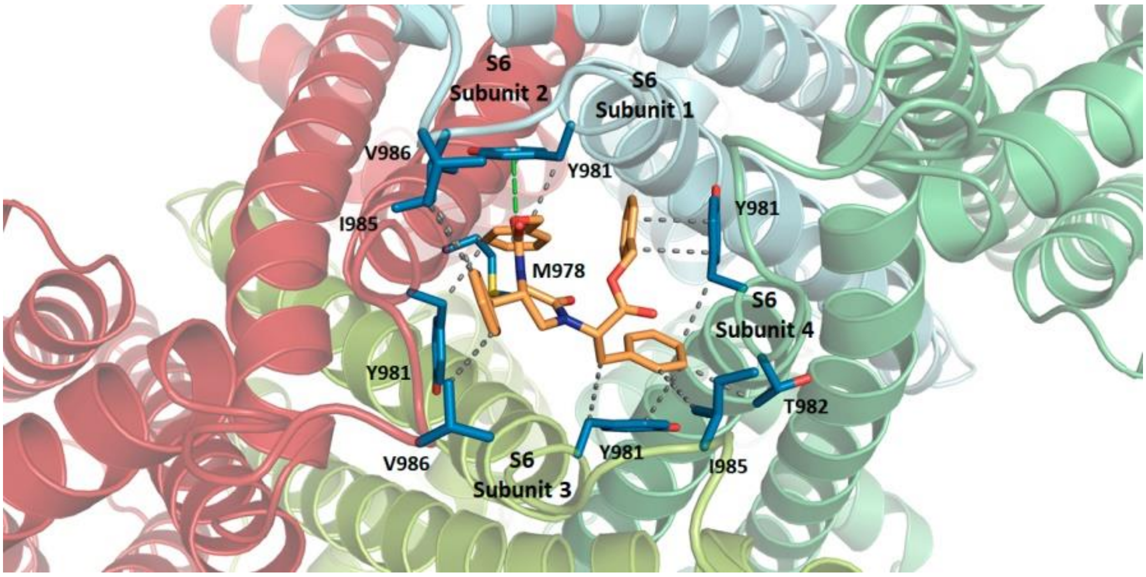

Hydrophobic Interactions

Index	Residue	AA	Distance	Ligand Atom	Protein Atom
1	978C Sub2	MET	3.78	21022	14832
2	981A Sub4	TYR	3.34	21004	4380
3	981A Sub4	TYR	4.00	21032	4383
4	981A Sub4	TYR	3.75	21031	4386
5	981B Sub3	TYR	3.82	21000	9628
6	981B Sub3	TYR	3.71	21005	9634
7	981C Sub2	TYR	3.50	21023	14876
8	981D Sub1	TYR	3.54	21013	20124
9	982A Sub4	THR	3.57	21005	4405
10	985B Sub3	ILE	3.86	21006	9691
11	985D Sub1	ILE	3.47	21019	20184
12	986C Sub2	VAL	3.70	21016	14959
13	986D Sub1	VAL	3.88	21018	20203

π-Stacking

Index	Residue	AA	Distance	Angle	Offset	Type	Ligand Atoms
1	981D Sub1	TYR	4.13	24.84	0.41	P	21013, 21020, 21021, 21022, 21023, 21024

Figure S7. Detailed view of the binding of compounds **13a** to TRPM8 Site 2.

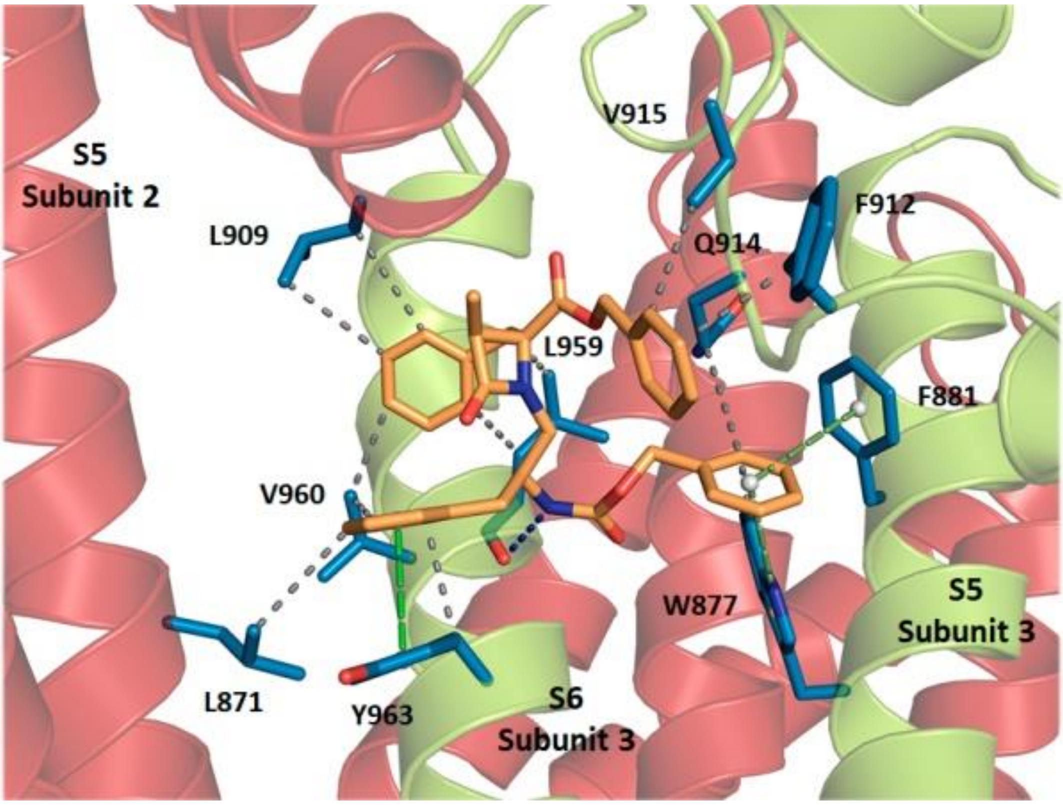

Hydrogen Bonds

Index	Residue	AA	Distance H-A		Distance D-A		Donor Angle	Protein donor?	Sidechain
1	959-Sub3	LEU	2.16	3.18	176.31			21010 [Nam]	9271 [O2]

π-Stacking

Index	Residue	AA	Distance	Angle	Offset	Type	Ligand Atoms
1	877-Sub3	TRP	4.81	70.19	1.64	T	21015, 21031, 21032, 21033, 21034, 21035
2	881-Sub3	PHE	5.34	85.91	0.65	T	21015, 21031, 21032, 21033, 21034, 21035
3	963-Sub3	TYR	4.05	11.53	1.61	P	21009, 21026, 21027, 21028, 21029, 21030

Hydrophobic Interactions

Index	Residue	AA	Distance	Ligand Atom	Protein Atom
1	871-Sub2	LEU	3.81	21028	13090
2	-Sub3	TRP	3.20	21035	7949
3	909-Sub2	LEU	3.71	21020	13754
4	909-Sub2	LEU	3.66	21019	13763
5	912-Sub3	PHE	3.46	21024	8552
6	914-Sub3	GLN	3.99	21035	8582
7	915-Sub3	VAL	3.63	21025	8602
8	959-Sub3	LEU	3.31	21016	9272
9	959-Sub3	LEU	3.66	20997	9277
10	960-Sub3	VAL	3.10	21018	9297
11	960-Sub3	VAL	3.36	21029	9297
12	963-Sub3	TYR	3.49	21030	9337

Figure S8. Detailed view of the binding of compounds **24a** to TRPM8 Site 1.

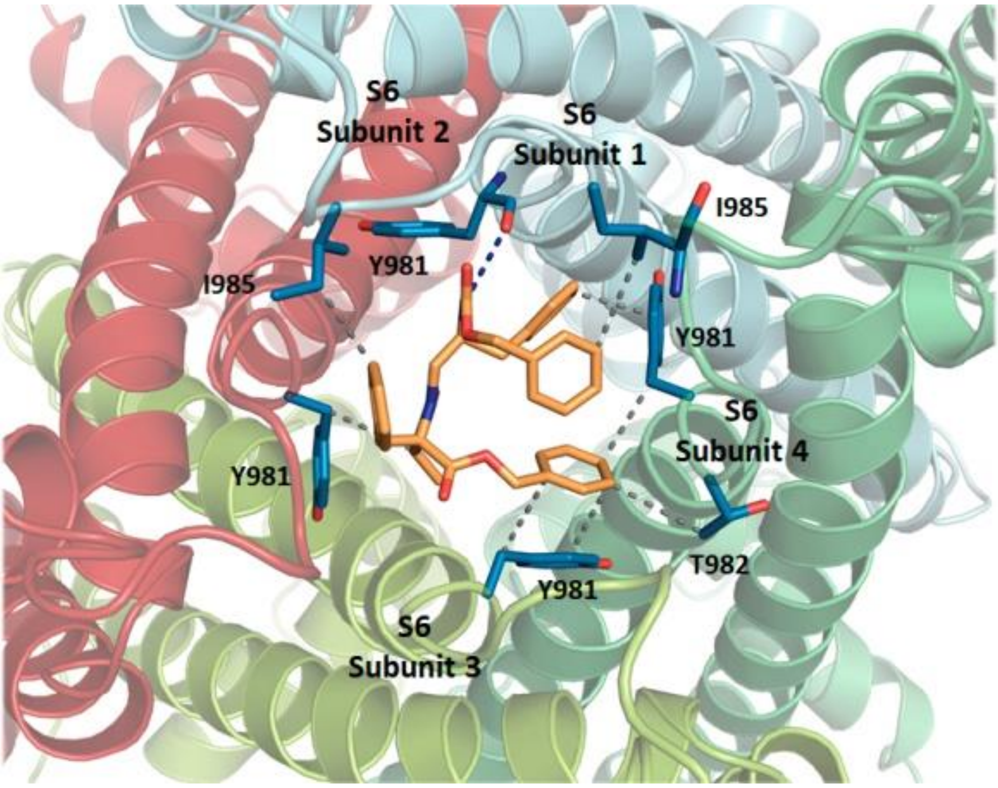

Hydrophobic Interactions

Index	Residue	AA	Distance	Ligand Atom	Protein Atom
1	981-Sub4	TYR	3.45	21023	4380
2	981-Sub4	TYR	3.12	21028	4386
3	981-Sub3	TYR	3.74	21007	9628
4	981-Sub3	TYR	3.59	21022	9634
5	981-Sub2	TYR	3.68	20993	14876
6	982-Sub4	THR	3.81	21022	4405
7	985-Sub4	ILE	3.64	21032	4443
8	985-Sub1	ILE	3.47	21017	20184

Hydrogen Bonds

Index	Residue	AA	Distance H-A	Distance D-A	Donor Angle	Protein donor?	Sidechain	Donor Atom	Acceptor Atom
1	981-Sub1	TYR	2.18	2.95	131.29			21010 [Nam]	20123 [O2]

Figure S9. Detailed view of the binding of compounds **24a** to TRPM8 Site 2.

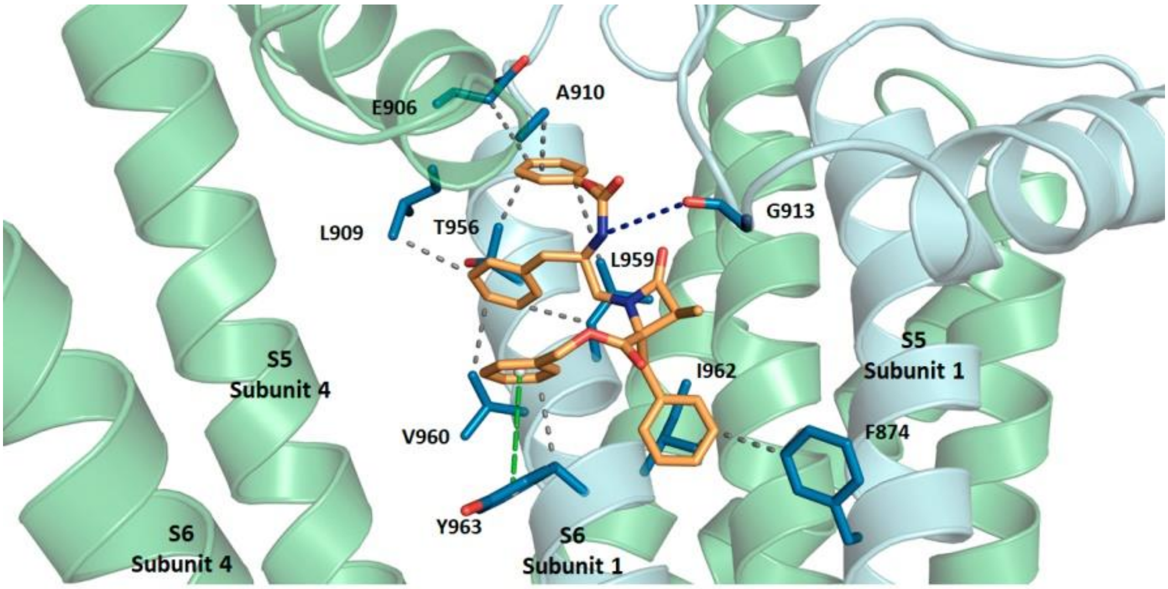

Hydrophobic Interactions

Index	Residue	AA	Distance	Ligand Atom	Protein Atom
1	874-Sub1	PHE	3.25	21017	18395
2	906-Sub4	GLU	3.99	21033	3211
3	909-Sub4	LEU	3.50	21027	3267
4	910-Sub4	ALA	3.20	21035	3277
5	956-Sub1	THR	3.12	21033	19725
6	959-Sub1	LEU	3.19	21029	19768
7	959-Sub1	LEU	3.68	21015	19773
8	960-Sub1	VAL	3.28	21028	19793
9	962-Sub1	ILE	3.21	21017	19819
10	963-Sub1	TYR	3.49	21024	19833

Hydrogen Bonds

Index	Residue	AA	Distance H-A		Distance D-A	Donor Angle	Protein donor?	Sidechain
1	913-Sub4	GLY	3.25	3.82	116.56		21010 [Nam]	19068 [O2]

π-Stacking

Index	Residue	AA	Distance	Angle	Offset	Type	Ligand Atoms
1	963-Sub4	TYR	4.70	28.08	0.87	P	21007, 21021, 21022, 21023, 21024, 21025

Figure S10. Detailed view of the binding of compounds **29a** to TRPM8 Site 1.

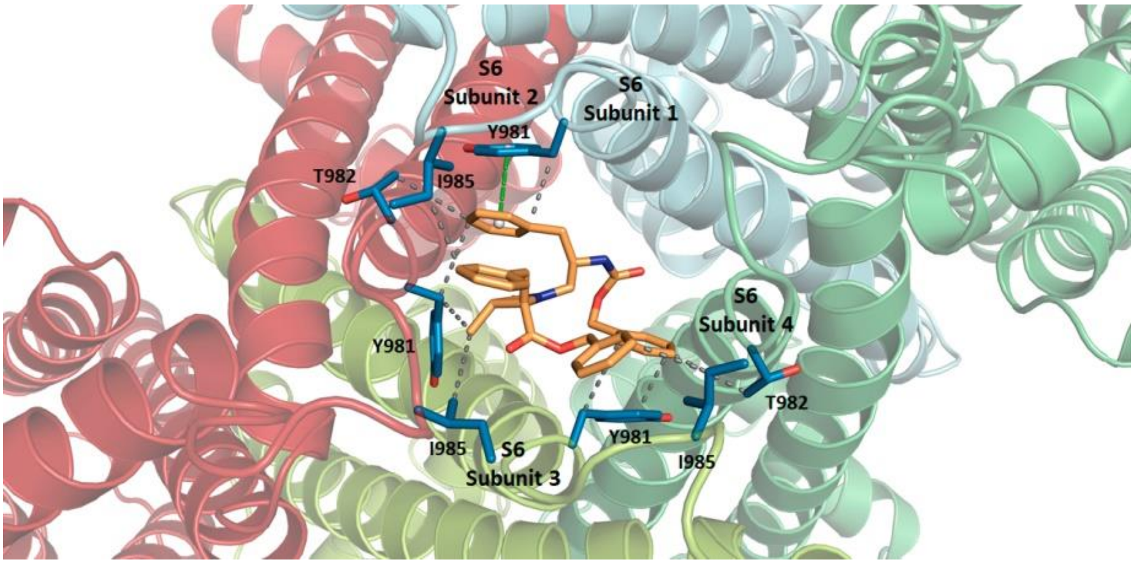

Hydrophobic Interactions

Index	Residue	AA	Distance	Ligand Atom	Protein Atom
1	981-Sub3	TYR	3.87	21031	9628
2	981-Sub3	TYR	3.48	21034	9634
3	981-Sub2	TYR	3.42	20998	14876
4	981-Sub2	TYR	3.76	21028	14876
5	981-Sub1	TYR	3.56	21009	20124
6	982-Sub4	THR	3.74	21033	4405
7	982-Sub2	THR	3.92	21028	14901
8	985-Sub3	ILE	3.36	21022	9688
9	985-Sub2	ILE	3.92	20998	14939
10	985-Sub1	ILE	3.25	21017	20184

π-Stacking

Index	Residue	AA	Distance	Angle	Offset	Type	Ligand Atoms
1	981-Sub1	TYR	4.32	25.61	1.68	P	21009, 21026, 21027, 21028, 21029, 21030

Figure S11. Detailed view of the binding of compounds **29a** to TRPM8 Site 2.

```
tr|U3JD03|U3JD03_FICAL      DEVRQWYMNGSKYFSDLWNVMDTLAIFYFIAGIVFRLHSD-ESSWYSGRVIFCLDYIVFT
sp|Q7Z2W7|TRPM8_HUMAN       DEVRQWYVNGVNYFTDLWNVMDTLGLFYFIAGIVFRLHSSNKSSLYSGRVIFCLDYIIFT
sp|Q8R455|TRPM8_RAT         DEVRQWYMNGVNYFTDLWNVMDTLGLFYFIAGIVFRLHSSNKSSLYSGRVIFCLDYIIFT
                            *******:** :**:*********.:*************   ** ************:**
                           841       850       860       870       880       890       900
                            |         |         |         |         |         |         |
tr|U3JD03|U3JD03_FICAL      LRLIHIFTVSRNLGPKIIMLQRMMIDVFFFLFLFAVWMVAFGVARQGILRKNEHRWEWIF
sp|Q7Z2W7|TRPM8_HUMAN       LRLIHIFTVSRNLGPKIIMLQRMLIDVFFFLFLFAVWMVAFGVARQGILRQNEQRWRWIF
sp|Q8R455|TRPM8_RAT         LRLIHIFTVSRNLGPKIIMLQRMLIDVFFFLFLFAVWMVAFGVARQGILRQNEQRWRWIF
                            ***********************:************************      **.***

                           901     910       920       930       940       950       960
                            |       |         |         |         |         |         |
tr|U3JD03|U3JD03_FICAL      RSVIYEPYLAMFGQYPDDIDGTTYNFDHCTFSGNESKPLCVELDANNQPRFPEWITIPLV
sp|Q7Z2W7|TRPM8_HUMAN       RSVIYEPYLAMFGQVPSDVDGTTYDFAHCTFTGNESKPLCVELDEHNLPRFPEWITIPLV
sp|Q8R455|TRPM8_RAT         RSVIYEPYLAMFGQVPSDVDSTTYDFSHCTFSGNESKPLCVELDEYNLPRFPEWITIPLV
                            ***********                                   * ************

                           961     970       980       990      1000      1010      1020
                            |       |         |         |         |         |         |
tr|U3JD03|U3JD03_FICAL      CIYMLSTNILLVNLLVAMFGYTVGSVQENNDQVWKFQRFFLVQEYCSRLTIPFPFVIFAY
sp|Q7Z2W7|TRPM8_HUMAN       CIYMLSTNILLVNLLVAMFGYTVGTVQENNDQVWKFQRYFLVQEYCSRLNIPFPFIVFAY
sp|Q8R455|TRPM8_RAT         CIYMLSTNILLVNLLVAMFGYTVGIVQENNDQVWKFQRYFLVQEYCNRLNIPFPFVVFAY
                            *****************             ********:*******.**.
```

Figure S12. Alignment of *Fidecula albicolis*, human and rat TRPM8 channels, with identification of residues involved in the interaction with the heterocyclic compounds described. Conserved residues (green), non-conserved residues (red).

In vitro bioassays for the detection of antitumor activity

The aim of this assay is to evaluate the *in vitro* cytostatic (ability to delay or arrest tumor cell growth) or cytotoxic (ability to kill tumor cells) activity of the samples being tested.

CELL LINES

Name	Nº ATCC	Species	Tissue	Characteristics
A549	CCL-185	human	lung	lung carcinoma (NSCLC)
HT29	HTB-38	human	colon	colorectal adenocarcinoma
MDA-MB-231	HTB-26	human	breast	breast adenocarcinoma
PSN1	CRM-CRL-3211	human	pancreas	pancreas adenocarcinoma

EVALUATION OF CYTOTOXIC ACTIVITY USING THE SBR COLORIMETRIC ASSAY

A colorimetric assay, using Sulforhodamine B (SRB) reaction has been adapted to provide a quantitative measurement of cell growth and viability (following the technique described by Skehan et al. *J. Natl. Cancer Inst.* 1990, 82, 1107-1112 (DOI:10.1093/jnci/82.13.1107).

This form of assay employs 96-well cell culture microplates following the standards of the American National Standards Institute and the Society for Laboratory Automation and Screening (ANSI SLAS 1-2004 (R2012) 10/12/2011). All the cell lines used in this study were obtained from the American Type Culture Collection (ATCC) and derive from different types of human cancer.

A549, HT29, MDA-MB-231 and PSN1 cells were maintained in Dulbecco's Modified Eagle Medium (DMEM) supplemented with 10% Fetal Bovine Serum (FBS), 2mM L-glutamine, 100 U/mL penicillin, and 100 U/mL streptomycin at 37 ºC, 5% CO_2 and 98% humidity. For the experiments, cells were harvested from subconfluent cultures using trypsinization and resuspended in fresh medium before counting and plating.

A549, HT29, MDA-MB-231 and PSN1 cells were seeded in 96 well microtiter plates, at 5000 cells per well in aliquots of 150 μL, and allowed to attach to the plate surface for 18 hours (overnight) in drug free medium. After that, one control (untreated) plate of each cell line was fixed (as described below) and used for time zero reference value. Culture plates were then treated with test compounds (50 μL aliquots of 4X stock

solutions in complete culture medium plus 4% DMSO) using ten 2/5 serial dilutions (concentrations ranging from 10 to 0.003 μg/mL) and triplicate cultures (1% final concentration in DMSO). After 72 hours treatment, the antitumor effect was measured by using the SRB methodology: Briefly, cells were washed twice with PBS, fixed for 15 min in 1% glutaraldehyde solution at room temperature, rinsed twice in PBS, and stained in 0.4% SRB solution for 30 min at room temperature. Cells were then rinsed several times with 1% acetic acid solution and air-dried at room temperature. SRB was then extracted in 10 mM trizma base solution and the absorbance measured in an automated spectrophotometric plate reader at 490 nm.

Effects on cell growth and survival were estimated by applying the NCI algorithm (Boyd MR and Paull KD. *Drug Dev. Res.* 1995, 34, 91-109, DOI:10.1002/ddr.430340203). The values obtained in triplicate cultures were fitted by nonlinear regression to a four-parameters logistic curve by nonlinear regression analysis. . Using the absorbance measurements [time zero, (Tz), control growth, (C), and test growth in the presence of drug at the ten concentration levels (Ti)], the percentage growth is calculated at each of the drug concentrations levels. Percentage growth inhibition is calculated as:

[(Ti-Tz)/(C-Tz)] x 100 for concentrations for which Ti >= Tz

[(Ti-Tz)/Tz] x 100 for concentrations for which Ti < Tz

Three dose response parameters are calculated for each experimental agent (Table S4). Growth inhibition of 50 % (GI_{50}) is calculated from [(Ti-Tz)/(C-Tz)] x 100 = 50, which is the drug concentration resulting in a 50% reduction in the net protein increase (as measured by SRB staining) in control cells during the drug incubation. The drug concentration resulting in total growth inhibition (TGI) is calculated from Ti = Tz. The LC_{50} (concentration of drug resulting in a 50% reduction in the measured protein at the end of the drug treatment as compared to that at the beginning) indicating a net loss of cells following treatment is calculated from [(Ti-Tz)/Tz] x 100 = -50.

Table S4. Anti-tumoral screening parameters

Compd.	Family	Parameter	Lung-NSCLC	Colon	Breast	Pancreas
			A549	HT29	MDA-MB-231	PSN1
**24a**	β-L	GI_{50}	3.29	4.16	>17.3	5.55
		TGI[a]	>17.3	>17.3	>17.3	>17.3
		LC_{50}	>17.3	>17.3	>17.3	>17.3
**29a**	β-L	GI_{50}	5.90	7.11	12.7	6.42
		TGI	>17.3	>17.3	>17.3	>17.3
		LC_{50}	>17.3	>17.3	>17.3	>17.3
**13ab**	2-KP	GI_{50}	>17.8	>17.8	>17.8	>17.8
		TGI	>17.8	>17.8	>17.8	>17.8
		LC_{50}	>17.8	>17.8	>17.8	>17.8
**15ab**	2-KP	GI_{50}	>17.8	>17.8	>17.8	6.4
		TGI	>17.8	>17.8	>17.8	>17.8
		LC_{50}	>17.8	>17.8	>17.8	>17.8
**30ab**	2-KP	GI_{50}	12.7	>17.3	>17.3	8.3
		TGI	>17.3	>17.3	>17.3	>17.3
		LC_{50}	>17.3	>17.3	>17.3	>17.3
**Doxorubicin. HCl**	–	GI_{50}	0.24	0.19	0.17	0.17
		TGI	0.64	0.64	0.41	0.48
		LC_{50}	2.93	>17.2	0.98	1.62

[a] When tested compounds do not reach the value of GI50, TGI and LC50 at the highest concentration tested (10 mg/mL), the values are expressed as > M [M maximum concentration of tested compound].
